# Global and regional climate change impacted Paleogene mammal faunas in Central Asia

**DOI:** 10.1038/s41467-026-77374-7

**Published:** 2026-09-08

**Authors:** Gemma Louise Benevento, Niels Meijer, Julia Brugger, Andreas Mulch, Thomas Hickler, Susanne A. Fritz

**Affiliations:** 1 Senckenberg Biodiversity and Climate Research Centre (SBiK-F), Frankfurt am Main, Germany; 2 German Centre for Integrative Biodiversity Research (iDiv) Halle-Jena-Leipzig, Leipzig, Germany; 3 Institute of Biodiversity, Ecology and Evolution, Friedrich Schiller University Jena, Jena, Germany; 4 Department of Geosciences, University of Tübingen, Tübingen, Germany; 5 Institute of Geosciences, Goethe University Frankfurt, Frankfurt am Main, Germany; 6 Cluster of Excellence (EXC 3121): TERRA – Terrestrial Geo-Biosphere Interactions in a Changing World, University of Tübingen, Tübingen, Germany; 7 Institute for Physical Geography, Goethe University Frankfurt, Frankfurt am Main, Germany

**Keywords:** Palaeontology, Palaeoclimate, Environmental impact, Evolutionary ecology

## Abstract

Global climate change has been linked to faunal turnover throughout geological history, but the effects of regional environmental change remain comparatively less understood. Central Asian environments changed repeatedly throughout the Paleogene (ca. 66 to 23 Ma) due to topographic reshaping and global climate change. Additionally, recently published geological records indicate that intense, regional aridification occurred during the mid-Eocene. We test the signatures of regional versus global climate change events in the fossil record, reconstructing Paleogene sampling-corrected taxonomic richness, faunal turnover, and body size change for all Central Asian mammals. We find three macroevolutionary transitions in mammal assemblages. One associated with regional Asian mid-Eocene aridification and two with global events — the Paleocene-Eocene Thermal Maximum (short-lived extreme warming 56 Ma) and the Eocene-Oligocene Transition (prolonged cooling 34 Ma). Our results indicate that both global and regional climate were associated with mammal evolution in the Paleogene of Asia. We also find that a previously described Eocene/Oligocene transition from Perissodactyla-dominated to Glires-dominated faunas — thought to be associated with cooling at the Eocene-Oligocene Transition – may be a sampling bias artifact.

## Abstract (web summary)

Here, the authors compare global and regional environmental change with mammalian turnover through the Paleogene of Central Asia. They identify three macroevolutionary transitions, one associated with regional aridification and two associated with global temperature changes.

## Introduction

Changes in the environment, particularly in climate, shape local and regional occurrences of species in space and time^1^. The fossil record provides a unique opportunity to examine the interactions between biodiversity and climate. In particular, it allows us to investigate biotic responses to past drastic changes in global climate^2^. Continental- to global-scale faunal changes in the fossil record are often attributed to major climatic events^3,4^, but linking long-term faunal diversity dynamics to global climate trajectories has proven difficult. While regional studies conducted at large spatial and temporal scales exist (e.g., entire continents over 10 million years or longer), these studies often compare regional fossil diversity dynamics to changes in global climate^3,5–8^ (but see refs. ^9–12^ for some notable exceptions). However, terrestrial assemblages commonly experience regional climatic conditions that are much more heterogeneous than global climate parameters would suggest. Consequently, regional habitat transitions should also be considered when studying terrestrial faunal diversity and composition^13,14^.

Here, we present a study on how regional mammalian biodiversity changed during the Paleogene, a key period in mammal evolution that lasted more than 40 million years, and compare these diversity dynamics to both global and regional climate records. In particular, we aim to address whether global and regional climatic events are associated with comparable degrees of biodiversity change in Asia. The fossil record is the only source of direct observations for species extinctions and assemblage turnover during extreme and protracted climate transitions. Therefore, resolving this question from the perspective of the fossil record is crucial for informing our understanding of how ongoing global climate change might affect future species distributions in specific regions, and in turn shift biodiversity patterns.

Two global climate change events within the Paleogene have been shown to correlate well with trends in mammal evolution across many parts of the globe, including Asia: the Paleocene-Eocene Thermal Maximum (PETM; 56.0 million years ago [Ma]) and the Eocene-Oligocene Transition (EOT; 33.9 Ma). The Paleocene-Eocene Thermal Maximum was a geologically rapid ( ~ 100 thousand years [kyr]) hyperthermal event concurrent with the Paleocene/Eocene boundary, recognized globally by a pronounced, negative carbon isotope (δ^13^C) excursion in the ocean-atmosphere system^15–17^. The PETM was characterized by global warming of ~ 5−10 °C due to a massive perturbation of the global carbon cycle^16–18^. Changes to mammal faunas and decreases in mammal body sizes with increasing temperatures have been recorded at the local level across multiple continents^19–21^. In Asia, pollen records show a greening of the continental interior deserts^22^, and fossil mammal assemblages suggest a substantial reduction in the proportion of ‘archaic’ eutherian mammals and the subsequent diversification of modern eutherian clades across the Paleocene/Eocene boundary^23^. Additionally, mammal dispersal events out of Asia or the Indian subcontinent into novel Holarctic regions are recognized from this time period^24–26^.

The Eocene-Oligocene Transition was a prolonged global cooling event ( ~ 400 kyr) close to the Eocene/Oligocene boundary, which was accompanied by growth of the Antarctic ice sheet, sea-level drop and continental aridification^27–30^. The EOT has also been widely linked to faunal turnovers across the globe^5,31,32^, including in Asia, where significant changes in mammal faunas across the Eocene/Oligocene boundary have been reported^5,23,33^. Previous studies have suggested a transition from mammal assemblages dominated by large-bodied Perissodactyla (odd-toed ungulates such as horses, tapirs, rhinoceroses and extinct relatives) to those dominated by smaller-bodied Glires (Rodentia, Lagomorpha—such as rabbits, hares and pikas—and extinct relatives), with Glires thought to have been better adapted to a drier Oligocene environment with fewer resources. This proposed turnover in mammal assemblage composition coinciding with the EOT, primarily recognized from Mongolia and the Inner Mongolia regions, has been coined the ‘Mongolian Remodeling’^5^. This event has also been temporally correlated to extinction and immigration events in Europe, collectively known as the ‘Grande Coupure’^5,33^.

Despite the prevailing theory that major aridification occurred at the EOT, recent paleoenvironmental records indicate that drying had already occurred in Central Asia during the middle Eocene, because of the modification of atmospheric moisture transport due to the growing Tibetan Plateau and the retreating inland Paratethys Sea^34^. A suite of geological records including increases in windblown dust and steppe-desert pollen^35,36^, increasing salinity in lakes^36^ and a decrease in weathering intensity^37^ indicate that this drying occurred on a regional scale, and was likely confined to a region of Central Asia above 30° N. At lower latitudes there is evidence for the expansion of swamps and freshwater lakes indicating a wetter climate due to intensified summer monsoons^34,38^.

**Fig. 1:**
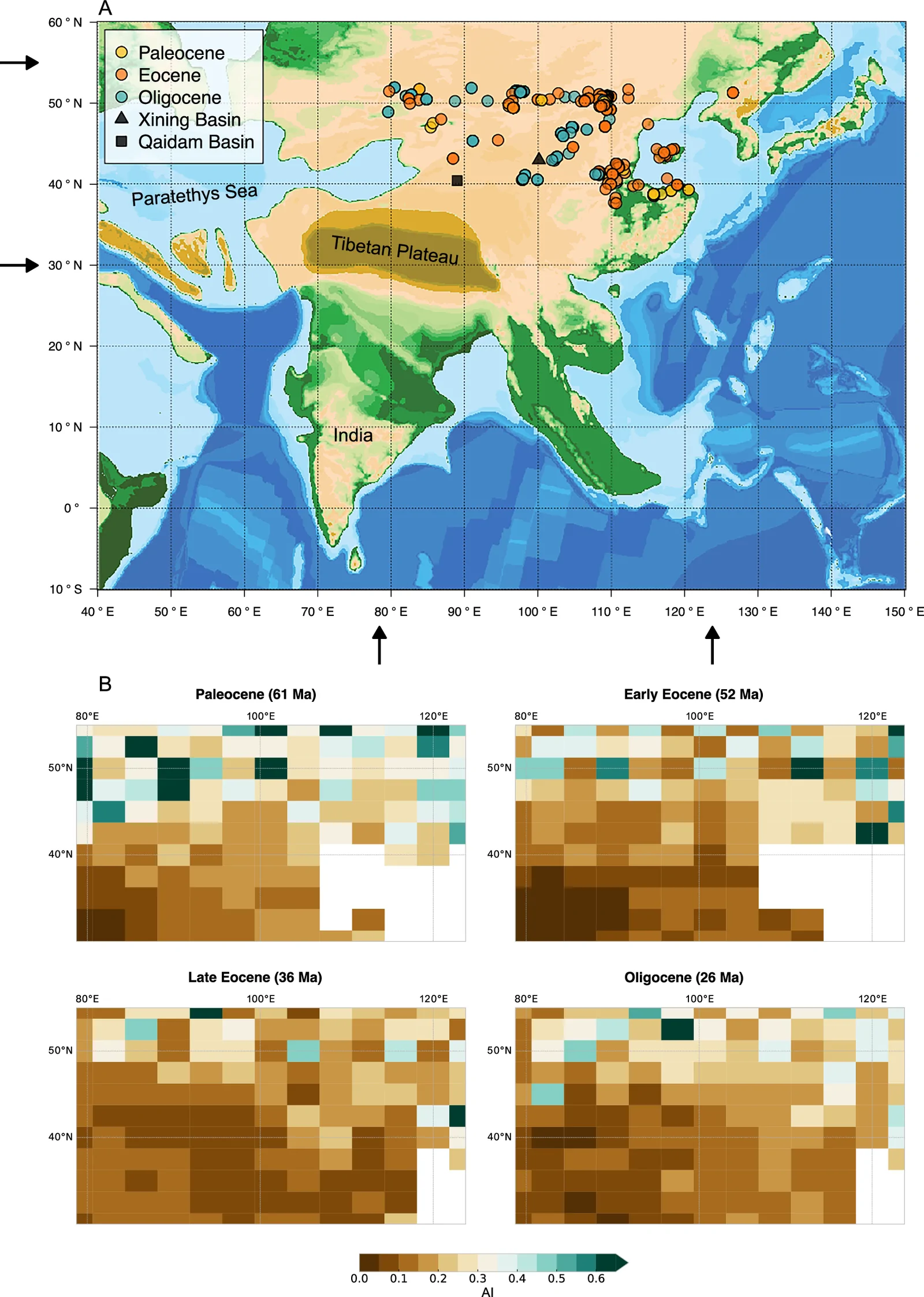
An Eocene palaeomap showing fossil localities and maps of four selected Cenozoic time periods showing the aridity index (AI) as calculated from climate model simulations. **A** Palaeomap of the Asian continent for the middle Eocene (ca. 40 Ma). Circle points indicate fossil sites included in this study, colored by geological period (yellow; Paleocene, orange; Eocene, blue; Oligoene). The black triangle and square indicate the Xining and Qaidam Basins, respectively, where data on pollen (Fig. 4D) and weathering (Fig. 4C) were collected^35,37^. The basis for the palaeomap (reproduced with permission) and the reconstructed palaeocordinates for our fossil sites and the basin records were extracted from https://map.paleoenvironment.eu^76,77^ (palaeomap re-plotted in R). **B** Maps of the simulated aridity index for ‘CO_2_ Scenario 1’^43^ for the Paleocene (61 Ma), early Eocene (52 Ma), late Eocene (36 Ma) and Oligocene (26 Ma)^42^. For reference, the aridity index defines drylands as regions with AI < 0.65, including specifically dry-subhumid regions (0.5 ≤ AI < 0.65), semi-arid regions (0.2 ≤ AI < 0.5), arid regions (0.05 ≤ AI < 0.2), and hyper-arid regions (AI < 0.05). The arrows on **A** denote the relative palaeolatitudinal and palaeolongitudinal extent of these climate maps.

Given this recent recognition of pre-EOT regional aridification, we hypothesize that changes in mammal biodiversity in Central Asia may have already been evident by the middle Eocene, and that mammal faunas responded to these changing regional climatic conditions at magnitudes comparable to the global climatic events that occurred at the start (PETM) and the end (EOT) of the Eocene. We investigated the entire Paleogene (66-23 Ma) and focused on Central Asia, defined here as Mongolia and China north of 30° N (Fig. 1), broadly following Barbolini et al.^39^. We estimated sampling-corrected standing richness (number of genera and species within geological time intervals) and compositional change (turnover in genera and species among subsequent time intervals), as major changes in biodiversity should encompass shifts in both number and identity of taxa. In addition, we investigated changes in the body size frequency distribution of the region through time, because mammalian body size is closely linked to climate conditions through physiological and ecological requirements, and shifts in assemblage-level body size patterns have been linked to past climatic transitions^14,40^. Finally, we compared our data with a re-analysis of existing climate model simulations (Fig. 1B) and published palaeoenvironmental records, to test the driving mechanisms of faunal change. We detected profound macroevolutionary changes among mammals coinciding with the PETM, the regional middle Eocene aridification event, and the EOT, suggesting that climate change was associated with Central Asian Paleogene mammal evolution. In addition, we found evidence that a moderate transition to faunas richer in Glires rather than in Perissodactyla in fact occurred concurrent with regional mid-Eocene aridification instead of during the EOT.

## Results

**Fig. 2:**
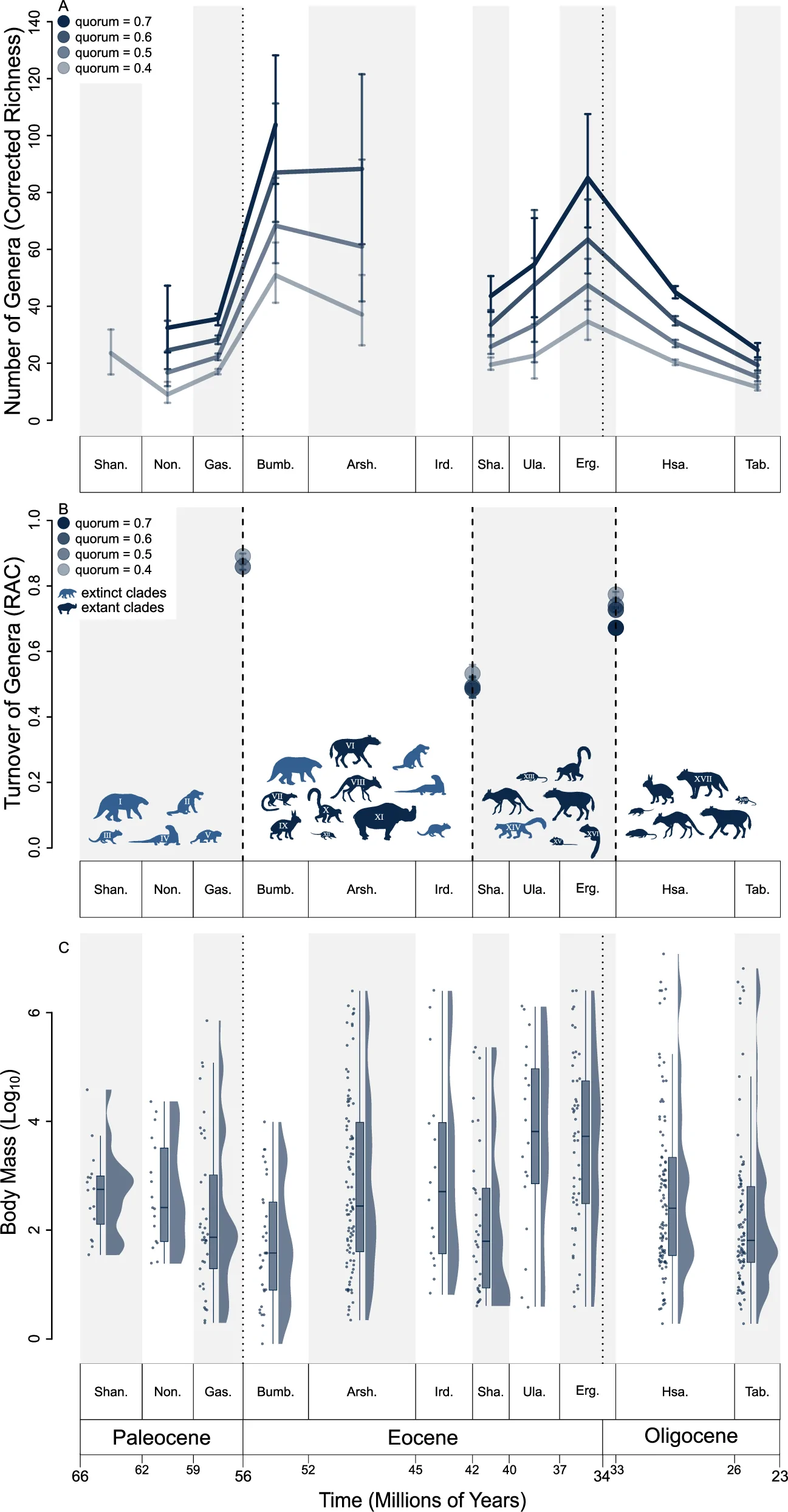
Sampling-corrected estimates for richness, turnover, and body mass for total Mammalia, spanning the Paleocene, Eocene, and Oligocene Epochs and corresponding Asian Land Mammal Ages (ALMAs). **A** Sampling-corrected richness in mammal genera for each ALMA (indicated by grey background shading) at quorums 0.4 - 0.7. Mean values are plotted as the center point derived from 500 technical bootstrap replicates. Error bars show 95% (percentile) confidence intervals also derived from 500 technical bootstrap replicates to assign fossils that span more than one ALMA to a randomized single ALMA within their age range. The absence of sampling-standardized richness results presented for the Irdinmanhan (Ird.) time interval (and for higher quorum values in a few other intervals) reflects a lack of reliably sampled data. Dotted vertical lines indicate epoch borders. **B** Faunal turnover based on mammal genera through time (pairwise bin comparisons using the relative abundance corrected [RAC] method) among four large time bins indicated by grey background shading and dotted vertical lines. Point color shading indicates tested quorum levels of 0.4–0.7. Center points represent a mean and error bars represent 5% and 95% percentile (quantile) confidence intervals calculated from 100 replicate data simulations (see Figs. S5 and S6A for turnover between individual ALMA intervals at lower quorums). Silhouettes indicate clade-level assemblage composition, depicting a selected typical mammal for each extinct vs. extant clade of mammals (status indicated by color shading). Silhouettes represent I, Pantodonta; II, 'Condylarthra'; III, Stem Glires; IV, Cimolesta; V, Multituberculata; VI, Perissodactyla A; VII, Rodentia A; VIII, Artiodactyla, IX, Lagomorpha; X, Primates; XI, Perissodactyla B; XII, Eulipotyphla; XIII, Rodentia B; XIV, Creodonta; XV, Rodentia C; XVI, Rodentia D; XVII, Carnivora; XVIII, Rodentia E. **C** Species-level body mass (log_10_-transformed) across ALMA intervals (indicated by grey background shading). Each raincloud plot shows individual species data points (left), box and whisker plots with a center showing the median, the bounds of the box showing the interquartile range and the wiskers showing the interval containing 90% of the data (middle), and a bisected violin plot, illustrating the approximated frequency distribution of individual values (right). Dotted vertical lines indicate epoch borders. Time interval abbreviations: Shan., Shanghuan; Non., Nongshanian; Gas., Gashatan; Bumb., Bumbanian; Arsh., Arshantan; Ird., Irdinmanhan; Sha., Sharamurunian; Ula., Ulangochuan; Erg., Ergilian; Has., Hsandagolian; Tab., Tabenbulakian. Silhouette images are by Dmitry Bogdanov (modified by T. Michael Keesey) (*Arctocyon*), Ferran Sayol (*Apodemus sylvaticus*, *Lemur catta*, *Nesomyidae*, *Rattus rattus*), Inessa Voet (*Solenodon paradoxus*), Margot Michaud (*Enhydrocyon stenocephalus*, *Pteronura brasiliensis*), Nobu Tamura (*Anagale*), Nobu Tamura (vectorized by T. Michael Keesey) (*Meniscoessus robustus*), Steven Traver (*Barylambda faberi*, *Embolotherium*, *Oryctolagus cuniculus*), T. Michael Keesey (after C. De Muizon) (*Diacodexis*), Xavier A. Jenkins (*Tamiops*), Zimices (*Paramys delicatus*), and others (*Plagiolophus*, *Vulpavus ovatus*). All silhouettes were recoloured. *Anagale, Arctocyon*, *Lemur catta*, *Nesomyidae*, *Solenodon paradoxus*, *Enhydrocyon stenocephalus*, *Meniscoessus robustus*, *Plagiolophus*, *Barylambda faberi*, *Embolotherium*, *Oryctolagus cuniculus* and *Diacodexis* silhouettes were flipped horizontally. CC BY-NC-SA 3.0 https://creativecommons.org/licenses/by-nc-sa/3.0/ (see also https://www.phylopic.org/collections/1af9e6c4-e693-0731-3ff4-93f8297ea573 and https://www.phylopic.org/permalinks/c34a59bd0b34d24b36c57275ebb831571cd27db96916f1378aeda35a0cbe441d for a permanent record of these silhouettes).

**Fig. 3:**
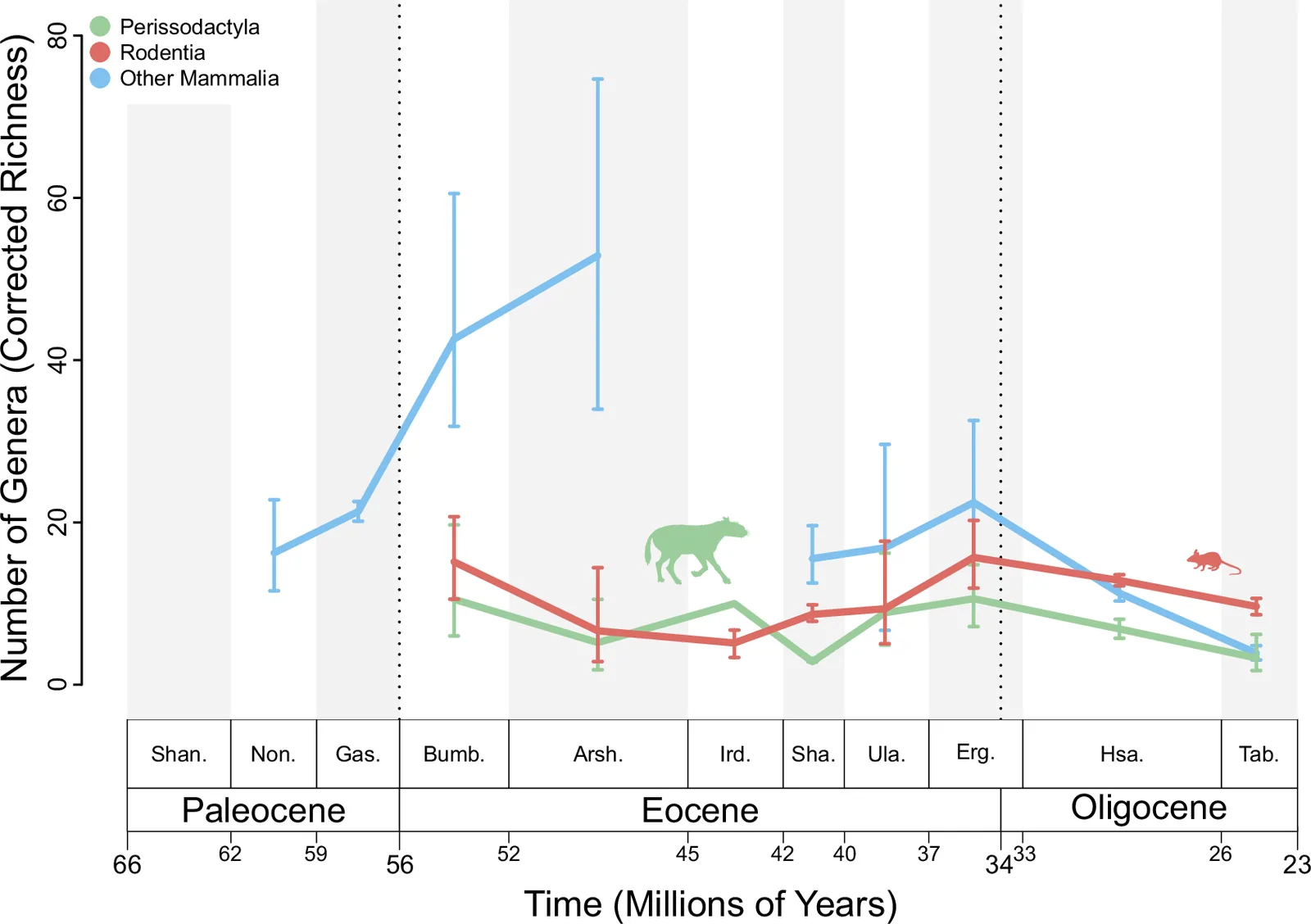
Sampling-corrected richness for mammal clades, spanning the Paleocene, Eocene, and Oligocene Epochs and corresponding Asian Land Mammal Ages (ALMAs). Richness curves include Rodentia (red), Perissodactyla (green) and ‘other Mammalia’ (all other mammal clades excluding Rodentia and Perissodactyla; blue). Richness estimates are based on genus-level data and shown for each ALMA (indicated by grey background shading) at quorum 0.5. Mean values are plotted as the center point and are derived from 500 technical bootstrap replicates. Error bars show 95% (percentile) confidence intervals also derived from 500 technical bootstrap replicates to assign randomized single ALMAs to fossils that span more than one ALMA. Dotted vertical lines indicate epoch borders. ALMA abbreviations are explained in Fig. 2. Silhouette images are by Ferran Sayol (*Nesomyidae*) and others (*Plagiolophus*). Silhouettes were recoloured and flipped horizontally (see also https://www.phylopic.org/collections/b1781bac-8e0b-05ce-3879-17df30271057 for a permanent record of these silhouettes).

We identified three pronounced shifts in Paleogene mammalian richness and turnover that each coincided with the timing of either enhanced global or regional climate change: (i) the Paleocene/Eocene boundary, (ii) the middle Eocene, and (iii) the Eocene/Oligocene boundary (Figs. 2 and 3). The results for sampling-corrected estimates of richness, turnover, and patterns of body size change across mammalian genera were all stable when a suite of sensitivity analyses were performed (Supplementary Note 1: Sensitivity Analyses). These sensitivity analyses included varying the taxonomic level of the analysis (species richness, Fig. S1), the handling of the temporal uncertainty of the fossil occurrences (Fig. S2A), the geographic extent of the analyzed region (Fig. S2B, C), and the methodologies for reconstructing faunal turnover (modified Forbes; Fig. S3A, Sørensen; Fig. S3B, C) and body size patterns (total; Fig. S4, subsampled; Fig. S5; see Table S1 and Supplementary Note 2: Body Size Patterns Through Time). In addition, results were consistent across different mammalian clade definitions and other settings for diversity reconstruction (Figs. S6, S7, S8, S9 and S10A, B) and were not biased by the geographic area covered by fossil locations for each individual time period and clade (Figs. S11 and S12, Table S2 and Supplementary Note 3: Geographic Area and Sampling-standardized Taxonomic Richness).

### Climate model re-analysis and summary of geo-environmental records

**Fig. 4:**
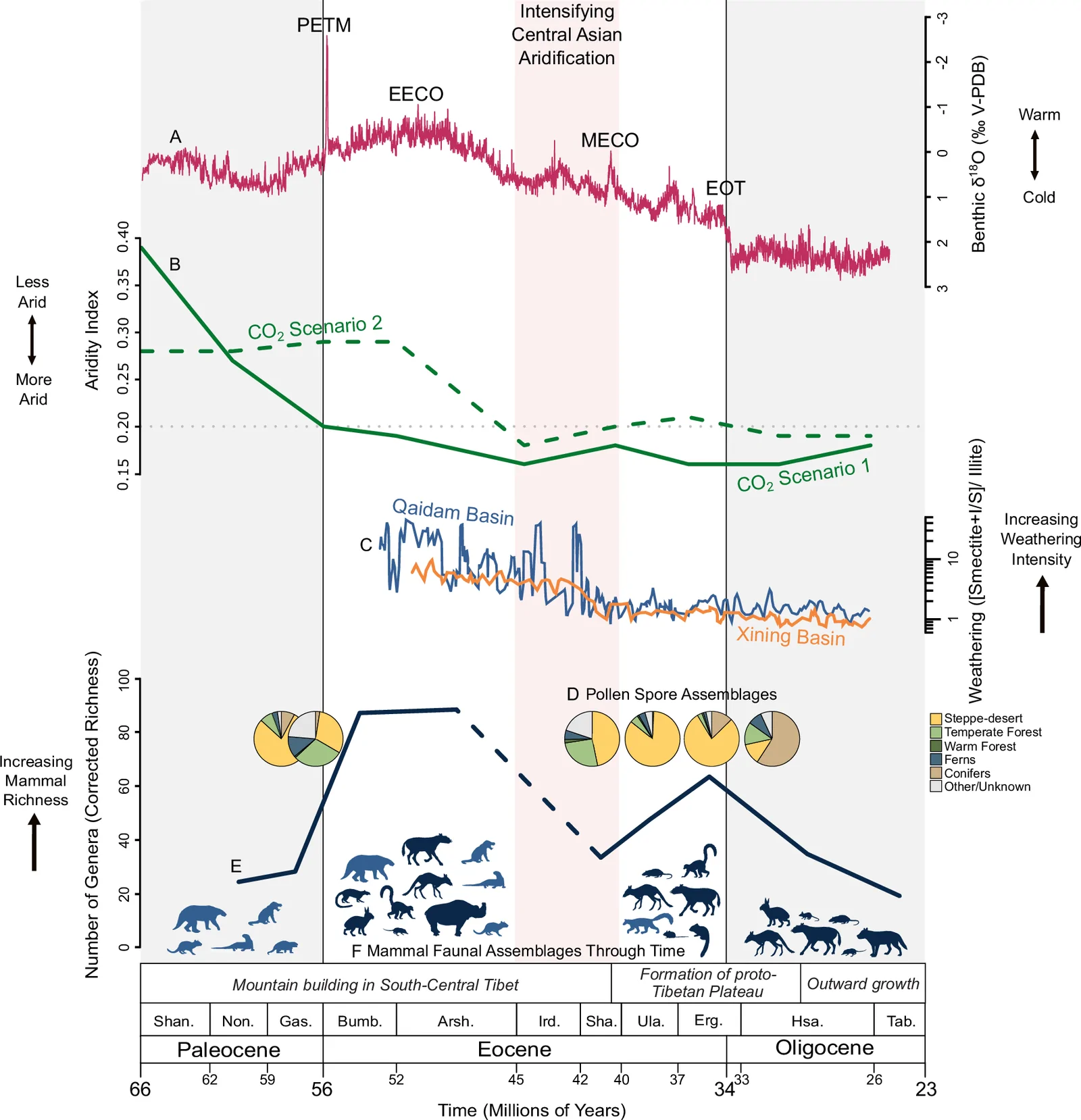
Summary figure of climatic and environmental changes alongside mammal diversity and turnover in Central Asia throughout the Paleogene, spanning the Paleocene, Eocene, and Oligocene Epochs. **A** Global benthic foraminifera δ^18^O record of Westerhold et al.^17^ as a proxy for global temperature, showing major palaeoclimatic events including the Paleocene-Eocene Thermal Maximum (PETM), Early Eocene Climatic Optimum (EECO), Middle Eocene Climatic Optimum (MECO) and the Eocene-Oligocene Transition (EOT). **B** Aridity index (AI = prec/*E*_p_) in Central Asia (averaged over 30° N − 55° N and 80° E − 120° E) simulated using the HadCM3 model^42^, calculated with two different *p*CO_2_ estimates (‘CO_2_ Scenario 1’^43^; solid green line and ‘CO_2_ Scenario 2’^42^; dashed green line). The horizontal dashed grey line represents an AI of 0.2, with environments below that classified as arid. **C** Weathering intensity (a palaeoenvironmental proxy for aridity) in the Qaidam Basin (blue) and Xining Basin (orange; Fang et al.^37^). Both basins are marked on our palaeomap (Fig. 1A).**D** Proportional percentage of pollen ecotypes through time in the Xining Basin (a palaeoenvironmental proxy for aridification from Meijer et al.^35^). **E** Sampling-corrected mammal richness through time in Central Asia (Mongolia and China above 30° N), summarizing results for genera at quorum 0.4 (Fig. 2A). The dashed section indicates a time bin that contains too few fossils for reliable diversity estimation. **F** Graphical representation of the main clades comprising mammal faunas through time, showing mammal faunal turnover between the Paleocene, early Eocene, late Eocene and Oligocene (see Fig. 2 for clade labels for the silhouettes). Light blue silhouettes represent mammals from extinct clades; dark blue silhouettes represent mammals from extant clades. The tectonic evolution of the Tibetan Plateau (from Ding et al.^41^) is also shown alongside geological time. The red vertical shaded bar indicates a period of more intense aridity in Central Asia. Vertical lines and grey shading indicate epoch borders. ALMA abbreviations are explained in Fig. 2. Silhouette images are by Dmitry Bogdanov (modified by T. Michael Keesey) (*Arctocyon*), Ferran Sayol (*Apodemus sylvaticus*, *Lemur catta*, *Nesomyidae*, *Rattus rattus*), Inessa Voet (*Solenodon paradoxus*), Margot Michaud (*Enhydrocyon stenocephalus*, *Pteronura brasiliensis*), Nobu Tamura (*Anagale*), Nobu Tamura (vectorized by T. Michael Keesey) (*Meniscoessus robustus*), Steven Traver (*Barylambda faberi*, *Embolotherium*, *Oryctolagus cuniculus*), T. Michael Keesey (after C. De Muizon) (*Diacodexis*), Xavier A. Jenkins (*Tamiops*), Zimices (*Paramys delicatus*), and others (*Plagiolophus*, *Vulpavus ovatus*). All silhouettes were recoloured. *Anagale, Arctocyon*, *Lemur catta*, *Nesomyidae*, *Solenodon paradoxus*, *Enhydrocyon stenocephalus*, *Meniscoessus robustus*, *Plagiolophus*, *Barylambda faberi*, *Embolotherium*, *Oryctolagus cuniculus* and *Diacodexis* silhouettes were flipped horizontally. CC BY-NC-SA 3.0 https://creativecommons.org/licenses/by-nc-sa/3.0/ (see also https://www.phylopic.org/collections/1af9e6c4-e693-0731-3ff4-93f8297ea573 and https://www.phylopic.org/permalinks/c34a59bd0b34d24b36c57275ebb831571cd27db96916f1378aeda35a0cbe441d for a permanent record of these silhouettes).

To explore the driving mechanisms of faunal changes in more detail, we compared the evolution of Central Asian mammals (Fig. 4E, F) with 1. global climate events identified in the marine benthic δ^18^O records (Fig. 4A;^17^); 2. the development of the Tibetan plateau^41^; 3. regional environmental changes revealed by climate model output (Figs. 1B and 4B); and 4. local weathering indices (Fig. 4C) and fossil pollen assemblages (Fig. 4D) in the Qaidam and Xining basins of Central Asia^35,37^. To compare the regional climatic conditions in Central Asia with the faunal changes, we used available climate model simulations that cover the entire Paleogene and incorporate the changing paleogeography and atmospheric CO_2_ concentrations (*p*CO_2_) at various time slices^42^. Based on the climate model output with a spatial resolution of 3.75° longitude and 2.5° latitude (see Fig. 1B), we calculated the aridity index (AI) as the quotient of precipitation and potential evaporation (see “Methods” for a detailed definition). This indicated semi-arid Paleocene climate conditions (0.2 ≤ AI < 0.5) in Central Asia that transitioned to an arid climate (0.05 ≤ AI < 0.2) in the early (‘CO_2_ Scenario 1’^43^) or middle Eocene (‘CO_2_ Scenario 2’^42^), and remained arid across the Eocene/Oligocene boundary (Figs. 1B and 4B; see Fig. S13 for more time intervals; for comparison, see a map of present-day AI in Zomer et al. ^44^). This suggests that desert-like conditions already existed in most of Central Asia before the EOT. However, we note that vegetation productivity and cover in a high-CO_2_ world, such as the Eocene, may have been somewhat higher than today for a given aridity index, potentially affecting the common interpretation of the aridity index as applied here, because plants have a higher water-use efficiency at high atmospheric *p*CO_2_^45^. Our re-analysis of these climatic model simulations showed consistently low average AI values from the mid-Eocene across different CO_2_ scenarios and subsections of our study region (Fig. S14), as well as decreased water availability (Fig. S15). The results corroborated previously published geological records^35,37^ that indicated significant Central Asian drying during the middle Eocene (Fig. 4C, D).

### Empirical results for mammals: Paleocene

Sampling-corrected Paleocene richness of genera and species (using coverage-based rarefaction and extrapolation^46^, comparable to shareholder quorum subsampling; SQS^47^) was relatively low among all Mammalia (Fig. 2A, Figs. S1 and S2A–C). Nevertheless, sampling-corrected turnover estimates showed that turnover in mammalian genera was high between all Paleocene Asian Land Mammal Age (ALMA) intervals (Fig. 2B and Fig. S3A–C). The first two Paleocene intervals (Shanghuan and Nongshanian) had the most restricted body size ranges of the entire Paleogene (Fig. 2C, Figs. S4 and S5). Mammals from these time intervals appear to occupy body sizes which fell within the mid-range of Paleogene mammal body sizes, meaning that mammals from the early Paleocene of Central Asia were neither particularly small nor large. There was a notable range expansion into smaller and larger body sizes during the final ALMA of the Paleocene (Gashatan; Fig. 2C, Figs. S4 and S5), however there was no statistically significant change in body size across this boundary (Welch’s two sample *t* test(44.6) = 1.27, *p* = 0.21, upper/lower 95% confidence intervals = 1.11/0.25, cohen’s d = 0.37; see Supplementary Data 1).

### Empirical results for mammals: Eocene

Across the Paleocene/Eocene boundary, sampling-corrected richness estimates of total Mammalia genera and species underwent an approximately threefold increase (Fig. 2A, Figs. S1 and S2A–C), and many of the extant clades became more prevalent in the fossil record (Figs. 2B and 3, Fig. S6). Sampling-corrected turnover in genera was also high across the Paleocene/Eocene boundary (Fig. 2B and Fig. S3A–C). Throughout the early Eocene (Bumbanian and Arshantan ALMAs), the number of genera remained at the highest levels of the Paleogene (Fig. 2A), while minimum, median and maximum body size increased gradually following a prior decrease across the Paleocene/Eocene boundary (Fig. 2C, Figs. S4 and S5), with a statistically significant change in body size distribution across the Bumbanian/Arshantan boundary (Welch’s two sample *t* test(79.45) = -4.32, *p* < 0.001, upper/lower 95% confidence intervals = -0.60/-1.63, cohen’s d = -0.89; see Supplementary Data 1). Early Eocene turnover remained low (although analyses for these bins were only possible at lower-than-optimal quorums; Fig. S3A, B).

By the middle Eocene (Sharamurunian), richness in genera and species had declined significantly to levels only slightly higher than those seen in the Paleocene (Fig. 2A, Figs. S1 and S2A–C). In addition, faunal turnover was also relatively high (Fig. 2B and Fig. S3A–C) and body size (minimum, median and maximum) appears to have decreased (Fig. 2C, Figs. S4 and S5; also for many subclades Figs. S7 and S8). Although the decrease in body size between the Irdinmanhan and Sharamurunian was non-significant (Welch’s two sample *t* test(25) = 1.63, *p* = 0.11, upper/lower 95% confidence intervals = 1.89/-0.22, cohen’s d = 0.5), the increase in median and maximum body size from the Sharamurunian to the Ulangochuan was significant (Welch’s two sample *t* test(30.1) = -3.14, *p* = 0.004, upper/lower 95% confidence intervals = -0.5/-2.5, cohen’s d = -0.92; see Supplementary Data 1). These mid-Eocene faunal changes mainly occurred between the Arshantan and Sharamurunian, but the exact timing remains unclear because the Irdinmanhan time interval, which lies between these two intervals, was the most poorly-sampled ALMA in our study, and therefore richness in this interval could not be estimated at our desired quorums. Analyzing Rodentia and Perissodactyla separately from all other mammals showed that the decline in number of genera was mostly due to a significant decline in the sum of the ‘other Mammalia’ clades (Fig. 3, Figs. S6 and S9). This resulted in Perissodactyla and Rodentia together comprising a much more substantial proportion of the fauna (approximately two thirds) compared to the early Eocene. Additionally, a moderate increase in Rodentia genera and a moderate decline in Perissodactyla genera resulted in a cross-over of their richness curves between the Irdinmanhan and Sharamurunian. From this point on, well before the EOT, Rodentia represented a slightly larger proportion of the mammalian assemblage compared to Perissodactyla (Fig. 3, Figs. S6 and S9).

Total Mammalia richness in genera and species recovered from the middle to late Eocene (Ulangochuan and Ergilian; Fig. 2A, Figs. S1 and S2A–C), although species richness may have taken longer to recover, remaining low in the Ulangochuan. Median and maximum body size also increased to their highest levels of the entire Paleogene during the late Eocene (Fig. 2C, Figs. S4 and S5). By the latest Eocene, all richness levels had rebounded to near early Eocene levels. Turnover in genera may have been reduced across the final stages of the Eocene as well; but the data were only sufficient to calculate sampling-corrected turnover at extremely low quorums (Fig. S3A, B).

### Empirical results for mammals: Oligocene

Sampling-corrected richness of total Mammalia genera and species declined substantially across the Eocene/Oligocene boundary and throughout the Oligocene to reach levels comparable with the Paleocene (Fig. 2A, Figs. S1 and S2A–C). Rodentia (and total Glires), Perissodactyla (and total ungulates) and ‘other Mammalia’ all showed gradual declines across the Eocene/Oligocene boundary and throughout the Oligocene (Fig. 3, Figs. S6, S9 and S10A, B). The more substantial decline among other Mammalia compared to Rodentia or Perissodactyla resulted in Rodentia comprising a slightly larger proportion of the Oligocene mammal faunas compared to other Mammalia, but there was no strong change in the proportion of Rodentia compared to Perissodactyla. Sampling-corrected turnover was high across the Eocene/Oligocene boundary (Fig. 2B and Fig. S3A–C). Median body size showed a significant decrease across the Eocene/Oligocene boundary (Ergilian/Hsandagolian; Welch’s two sample *t* test(98.28) = 3.29, *p* = 0.001, upper/lower 95% confidence intervals = 1.41/0.35, cohen’s d = 0.56; see Supplementary Data 1) and continued to decline (near-significant; Welch’s two sample *t* test(184.12) = 1.96, *p* = 0.051, upper/lower 95% confidence intervals = 0.87/0, cohen’s d = 0.29) throughout the Oligocene, but body size ranges remained stable (Fig. 2C, Figs. S4 and S5).

## Discussion

In contrast to previous reconstructions which have highlighted the importance of global climate change during the PETM and EOT for mammal evolution, we identify a third period of major mammal faunal change in Central Asia. This additional event occurred during the mid-Eocene and was distinct from the two global events. Comparison of these reconstructed evolutionary dynamics in Central Asian mammals with a climate model re-analysis and available geo-environmental records reveals that the mid-Eocene decline in mammal richness is coeval with a protracted phase of regional drying (Fig. 4). This drying is further linked to the reshaping of the Asian continent following the India-Asia collision. It is particularly noteworthy that this regional climate change had an apparently equal or greater impact on mammal faunas than the global climate events at the PETM and the EOT. Overall, we observe that global warming during the hothouse conditions of the PETM and early Eocene supported diverse mammal assemblages in Central Asia, whereas continental drying and global cooling resulted in lower richness and strong faunal turnover during the mid-Eocene and EOT. This may reflect a link between mammalian richness and primary productivity^13^, and shows that environmental changes resulting from both global and regional climate events were associated with Asian Paleogene mammal macroevolution.

The Paleocene was a period of general turnover for early mammal clades in Central Asia. The Paleocene faunal instability and high turnover of locally restricted taxa^23^ is likely a result of taxonomic and ecological diversification after mass extinction-related niche clearing at the Cretaceous/Paleogene (K/Pg) boundary^48,49^. The Paleocene saw a global radiation of many mammal orders, including numerous clades that did not survive to the present day. In Asia, these early placental mammals were common throughout the Paleocene and remained so until at least the mid-Eocene. We found that Asian mammals underwent their largest Paleogene radiation across the Paleocene/Eocene boundary; this differs from the more rapid increase seen directly after the K/Pg boundary in North America, where maximal Paleogene mammal richness was reached by the late Paleocene^50^. In Central Asia, we estimate that mammals underwent an approximate three-fold increase in taxonomic richness across the Paleocene/Eocene boundary, coupled with high faunal turnover, decreases in body size distributions and the evolution or arrival of numerous modern clades, possibly originating from southern Asia or the Indian subcontinent^51–53^. This dispersal and/or radiation event is tightly concurrent with the carbon isotope excursion marking the PETM, which may have catalyzed these longer-term faunal changes. Concurrent but distinct faunal changes in North America and Europe may indicate that shifting biomes as a result of global warming opened up intercontinental dispersal pathways at this time^25,26^. Detailed pollen records from Central Asia show an abrupt greening of the steppe-desert due to intensified monsoons during the PETM^22^, which may have played a role in driving the observed dispersal, faunal turnover and high richness in mammal assemblages. However, this peak in species richness persists until after the PETM and into the early Eocene, marking a period of faunal stability that can be linked to the long-term global warmth of the Early Eocene Climatic Optimum (EECO) and tectonic quiescence in Central Asia. A similar pattern is observed in flora and mammal fauna on the North American continent^4^.

The early Eocene peak in our richness estimates was followed by a mid-Eocene decline, high turnover and an overall body size decrease concurrent with regional aridification (i.e., a protracted phase of drying indicated by climate model simulations as well as regional weathering indices, culminating in a spread of steppe-desert vegetation at ~ 40 Ma^35,37^ [Fig. 4]). These environmental shifts have been linked to the growth of the Tibetan Plateau and retreat of the Paratethys Sea in Central Asia^35^, and according to our analyses are concurrent with the substantial mammal richness decreases and extinctions among (primarily) Paleocene and early Eocene clades, including Pantodonta, 'Condylarthra', Creodonta and Cimolesta. Previous analyses of Perissodactyla in this region found both the first occurrence of mesodont species and a decrease in raw (uncorrected) species richness immediately following this time interval and prior to the EOT, mirroring our results for total Mammalia species richness and further suggesting a protracted period of recovery^54^. Persistent dry environments which may have been unfavorable to many mammal clades could have inhibited the immediate replacement of these groups by other extant mammal clades. Alternatively or additionally, this aridification may have resulted in both the reduction of geological sections (e.g., the Irdinmanhan–Ulangochuian; ~ 45–37 Ma) and the contemporary richness decline and turnover of taxa, indicating environmental aridification as a common cause for rock record volume and fossil diversity^55,56^.

Subsequently, total mammal richness remained lower than it had been prior to the aridification event for the remainder of the Paleogene. Eocene members of extant clades did begin to increase in richness throughout the later stages of the Eocene (causing a steady increase in total Mammalia richness), presumably filling niches left vacant by their extinct predecessors, who mostly belonged to ‘archaic’ clades. This initial pattern of declining ‘archaic’ clades in the mid-Eocene seen here is mirrored on other continents^50,57,58^, meaning other global climatic factors such as long-term cooling throughout the Eocene or transient warming during the Middle Eocene Climatic Optimum (MECO; 40 Ma) may have played a role as well. However, the rather poorly-sampled Asian Irdinmanhan ( ~ 45.4–41.9 Ma) fossil record and dating uncertainties in land mammal ages across different continents inhibit a more accurate temporal correlation. Regardless, the concurrent mid-Eocene decreases in total Mammalia (and individual clade) body size and the delayed rebound of total Mammalia richness shown here for Asia strongly suggest a causal link with regional drying.

Concurrent with the EOT in Asia, the ‘Mongolian Remodeling’ has previously been described as a replacement of Perissodactyla-dominated faunas by Glires-dominated faunas, largely based on a literal reading of the raw fossil record of Mongolia and Inner Mongolia. Our sampling-corrected estimates revealed less dramatic clade turnover around the EOT than has previously been suggested for this region of Asia^5,23^. Although the raw fossil record shows a strong pattern of increases in abundance, uncorrected taxonomic diversity, and overall assemblage proportion for Rodentia and Lagomorpha after the EOT in Asia (Fig. S16)^5^, sampling-corrected data consistently reveal a different pattern (Fig. 3; also when restricting our data to Mongolia and Inner Mongolia to better replicate the study site of Meng & McKenna^5^; see Fig. S9). Our results suggest that the purported extreme increase in Rodentia and Lagomorpha fossil abundance after the EOT can at least partially be attributed to biases in the fossil record, such as an increased number of fossiliferous formations, and subsequently fossils, in the Oligocene compared to the late Eocene [Fig. S17], or possibly more intensive sampling of smaller species via collection techniques such as sieving. Under the common-cause hypothesis mentioned above^56^, a lower rock record volume in Asia during the mid-Eocene (which could be suggested by the observed lower number of fossiliferous locations in our dataset; see Table 1) and lower raw fossil diversity (Figs. S16 and S17 and Supplementary Note 4: Raw Diversity Patterns) would both be connected to environmental aridification, which could accentuate increases in the subsequent Oligocene in total diversity and rock volume that are apparent in our datasets. However, this would not immediately explain the differences in richness trajectories in the different clades. Moreover, the previously postulated turnover from Perissodactyla-dominated to Rodentia-dominated faunas has been attributed to Rodentia being better adapted to a drying environment^5^. Within our dataset, however, Perissodactyla are consistently found in localities that are reconstructed as more arid when compared to localities with Glires (statistically significantly lower modeled AI at locations where Perissodactyla fossils are recovered: Table S3 and Figs. S18 and S19. See also Supplementary Note 5: Aridity Index (AI) Distributions for Perissodactyla, Rodentia and Glires). This pattern suggests a more complex relationship than has previously been suggested, and the potential for a stronger arid adaptation of extant Glires compared to extinct ‘archaic’ clades would be worth investigating in the future.

**Table 1:** The number of fossil occurrences, unique genera and species, and localities for each Asian Land Mammal Age (ALMA) across the mammal occurrence datasets

			Genus-level Dataset			Species-level Dataset		
**ALMA Name**	**ALMA Lower Boundary**	**ALMA Upper Boundary**	**Fossil Abundance**	**Raw Richness**	**Localities**	**Fossil Abundance**	**Raw Richness**	**Localities**
**Shanghuan**	66.00	62.20	22	19	13	40	23	23
**Nongshanian**	62.20	59.05	45	22	30	23	22	12
**Gashatan**	59.05	56.00	124	49	25	109	57	21
**Bumbanian**	56.00	51.98	60	43	8	53	45	8
**Arshantan**	51.98	45.40	167	93	49	155	109	47
**Irdinmanhan**	45.40	41.90	50	43	17	23	21	11
**Sharamurunian**	41.90	39.65	75	40	22	74	52	22
**Ulangochuian**	39.65	36.55	28	19	7	24	20	7
**Ergilian**	36.55	33.10	126	61	31	114	72	30
**Hsandagolian**	33.10	25.80	722	129	134	716	136	120
**Tabenbulakian**	25.80	23.00	468	65	101	541	106	100

Although both Rodentia and Perissodactyla exhibit a similar decline in sampling-corrected number of genera across the EOT, Rodentia may indeed have become a larger component of mammal faunas during the Oligocene. However, this proportional change does not appear to have been the result of a decrease in Perissodactyla coupled with a concurrent increase in Rodentia and Lagomorpha, but rather of a much larger decline in other mammal clades compared to the declines seen in Rodentia and Perissodactyla (Fig. 3 and Fig. S9). Therefore, we do not find a strong pattern of turnover from more Perissodactyla-dominated to more Rodentia-dominated mammal faunas at the EOT boundary, which has previously been the defining factor of the Mongolian Remodeling^5^. If indeed there is such a turnover during the Paleogene of Central Asia, it may have occurred during the mid-Eocene, concurrent with the onset of regional Asian aridification (Fig. 4)^35^, although this is not a particularly strong pattern. These results mirror a previously recovered decline in raw (uncorrected) species richness in Perissodactyla millions of years prior to the EOT boundary during the Ulangochuian, named the Ulan Gochu Decline^54^. When the Glires clade is analyzed as a whole and compared with Perissodactyla in number of genera, Glires are always more diverse than Perissodactyla throughout the Paleogene (Fig. S10A, B). However, this pattern is expected based on a normal body size frequency distribution for mammals, as faunal assemblages should, in general, hold more small mammals than large mammals^50,59^. Nevertheless, we do observe turnover in mammal assemblages across the Eocene/Oligocene boundary in the sampling-corrected results (Fig. 2B and Fig. S3A–C), which could indicate an adaptation of mammalian assemblages to the colder and possibly drier coolhouse conditions of the Oligocene, as reflected by the spread of conifers in the pollen record of Central Asia^39^.

Our results re-examine the timing and magnitude of mammal faunal changes associated with the Mongolian Remodeling in Central Asia, pushing back fundamental clade turnover dynamics by >6 million years in line with an earlier aridification of the region, supported by geological records^35,37^ and climate model simulations. These findings add to a growing wealth of evidence that global as well as regional climate strongly influence mammal diversity, trait and assemblage dynamics throughout geological history and biogeographic regions. Crucially, we show that continental-scale changes in landscape and topography driving regional environmental change were likely at least as important for faunal diversity and composition as perturbations of atmospheric CO_2_ driving global warming or cooling.

Our results indicate that local and regional responses of diversity to ongoing and future climate change might not be easily predictable from global warming trends alone. Instead, identifying key threats to mammalian diversity will likely have to take regional or local changes in environmental conditions into account, including water availability, atmospheric moisture transport and biome shifts. Our research brings together analyses of geological and modeled past environmental change with estimates of mammal diversity, turnover and trait evolution, and serves to inform the formulation of more robust, testable hypotheses for mammal (or even tetrapod) assemblage-level responses to future climate and environmental change.

## Methods

### Fossil mammal occurrence data

Occurrence data were collected for the Paleocene to Oligocene of Central Asia (Mongolia and China above 30° North; see Fig. 1A) from the Paleobiology Database (PBDB; https://paleobiodb.org on 10.11.2022) and the NOW Database of Fossil Mammals (https://nowdatabase.org on 12.04.2022) and were supplemented with additional data collected from the primary literature (see Supplementary Data 2 for a bibliography of this primary literature). Localities were unified across the PBDB, NOW and primary literature datasets to ensure they were not duplicated. Fossil occurrence ages were updated to reflect the latest Stage or Asian Land Mammal Age (ALMA) time interval boundaries. Species and/or genera with uncertain determination were removed from their respective datasets. The resulting cleaned total genus-level dataset had 1887 occurrences, 393 unique genera and 426 unique localities, and the species-level dataset had 1872 occurrences, 537 unique species and 365 unique localities (Supplementary Data 3; see Fig. S1 and Supplementary Note 6: Sampling-standardized Richness of Species for species level results, and Table 1 for number of fossil occurrences, unique taxa and unique localities per ALMA across both datasets). All fossil mammal dataset cleaning and analyses were conducted in R version 4.3.1 (Supplementary Code 1).

### Sampling-standardized taxonomic richness of mammal assemblages

Raw genus- and species- level occurrence data were first divided into Asian Land Mammal Ages (ALMAs; see Table 1 and Supplementary Data 4 for time bins used). All richness analyses were run separately on the genus- and species-level occurrence datasets. As fossil occurrences are often dated imprecisely, we used a randomization technique to assign a single time interval within a fossil’s given age range to the individual fossil for analyses, where a numerical age was drawn at random from each fossil’s age distribution multiple times. This was done for each occurrence and then sampling-standardized richness was calculated for each ALMA. The age randomization and richness calculations were repeated 500 times to capture the variation in age estimates, and error bars were produced using 95% confidence intervals from the 500 richness results. Sampling-standardized richness was calculated using coverage-based rarefaction and extrapolation using the estimateD() function in the R package iNext^60^ (Supplementary Data 5). Data were either observed, subsampled or extrapolated to target coverage dependent on the underlying completeness of the sample (Supplementary Data 6). If data were extrapolated to more than twice the underlying sample size, these data were not used as they are likely to be unreliable^60^. The coverage-based rarefaction and extrapolation method samples time bins to an equal coverage (or quorum) of the underlying taxon pool, based on the taxon frequency distribution^46,47,61^. While no method is capable of perfectly reconstructing true richness, this approach provides one of the best options for estimating taxonomic richness in the fossil record to date^61^. Coverage-based rarefaction and extrapolation was performed for each ALMA at quorums 0.4−0.7 for total Mammalia (Fig. 2A), Rodentia, Perissodactyla and ‘other Mammalia’ (all other mammal clades excluding the Rodentia and Perissodactyla; Fig. 3; additional quorums in Fig. S6B, D and F), and additionally for Glires, ungulates and Artiodactyla (see Fig. S6A, C and E).

Additional sensitivity analyses were performed to test the robustness of our results using subsets of the original data. Sensitivity analyses included calculating sampling-corrected richness using four subsets of our full genus-level dataset as follows: (i) a subset of the total Mammalia dataset that additionally removed any occurrences which had an age estimate that spanned three bins, resulting in a more conservative dataset including only those occurrences with better age constraints (Fig. S2A), (ii) a subset of the total Mammalia dataset that additionally removed all occurrences east of 115° East, as this most easterly area of our study region may not have been as arid as the rest of the region (Fig. S2B), (iii) a subset of the total Mammalia dataset that included only Mongolia and Inner Mongolia (all occurrences east of 125° East and/or south of 37° North), making this subset a more direct replicate of the study of Meng & McKenna^5^ (Fig. S2C) and (iv) subsets of the Glires, Rodentia, Perissodactyla, and ‘other Mammalia’ datasets that included only Mongolia and Inner Mongolia, again to better assess differences between our results and those from Meng & McKenna^5^ (Figs. S9 and S10B).

Good’s *u*, a sample coverage estimator^62^, was calculated to assess the level of sampling coverage for each ALMA (Supplementary Data 7). We used the ‘Chao’ modification to the Good’s *u* calculation, which uses singletons and doubletons (species that appear in only one or two locations, respectively) to estimate coverage^62,63^.

Previous research has uncovered a correlation between sampling-standardized richness and the geographic extent of the localities included within a given time interval^64^. We tested for a correlation between sampling-corrected richness (quorum 0.4 for total Mammalia and 0.5 for subclades) and geographic extent for each time bin and across all genus-level datasets. Geographic extent was calculated using minimum spanning tree (MST^65^) length among spatial locations using the function MSTDist() in the package GeoRange^66^ in R. We recovered no such correlation in any of our datasets (see Table S2, Figs. S11 and S12 and Supplementary Note 3: Geographic Area and Sampling-standardized Taxonomic Richness for non-significant results of correlation tests across total Mammalia and all clades). We therefore concluded that geographic extent is likely not responsible for the richness patterns presented here and therefore did not further standardize our richness estimates to a consistent MST length. It is likely that differences in geographic extent of our data across time bins was mitigated by the regionality of our study, and that this effect has more impact on richness results on larger spatial scales.

### Sampling-standardized turnover of mammal assemblages

Interval-to-interval turnover in mammalian genera was calculated with the genus-level occurrence dataset for adjacent ALMAs (Supplementary Data 8). In addition, we calculated turnover among adjacent time bins for four larger intervals, which were chosen based on major changes in mammal assemblages (as indicated by turnover among ALMAs) and known periods of environmental change (based on the other results in this study; defined as follows: Paleocene, early and middle Eocene until the end of the Irdinmanhan, middle and late Eocene from the Sharamurunian, and Oligocene; see Supplementary Data 4). These larger time bins allowed for a bigger sample size in each bin and were more robust for analyses of turnover. When analyzing turnover, incomplete sampling (as is expected in the fossil record) artificially decreases similarity between two faunas, and therefore would increase turnover estimates between bins^67^. To correct for uneven and incomplete sampling, we used a modification of the relative abundance corrected (RAC) method^67^. Originally developed for estimates of beta-diversity (*β*) using true taxonomic abundance data between faunas of the same age, we used number of sites (equivalent to PBDB ‘collections’) per time bin with occurrences of taxa across our entire study region as our input ‘abundance data’ (as was the case for our coverage-based rarefaction and extrapolation analyses). We then performed pairwise comparisons between bins using quorums 0.4−0.7 for larger bins (smaller bins were also tested and are presented in Fig. S3A, B), and using both a modified Forbes distance matrix^68^ (Fig. 2B and Fig. S3A) to account for nestedness between bins (by returning a value of 0 if species were only gained or lost due to diversification, migration or extinction, but the original species were not ‘replaced’), and a Sørensen distance matrix^69^ (Fig. S3B, C). Using RAC, a raw comparison of the differences between the samples (in our case, two adjacent time intervals) was calculated. The abundance distributions of these samples are assessed, and a new sample matching these abundance distributions is simulated using the entire dataset (e.g., using species from both time intervals). Here, the true overlap between these new simulated datasets is 100%, and any differences between the samples are a product of sampling bias. The difference between these two simulated samples was then calculated using the same distance metric. This process was repeated 100 times and a mean and 95% confidence intervals were calculated from these technical bootstrap replicates. Finally, the result from the simulated datasets was subtracted from the results obtained from the real fossil samples^67^ (see also Supplementary Note 7: Sampling-standardized Turnover).

### Mammal measurement data and body mass estimates

Lower first molar (m1) length and width were collected for 389 of the 537 species in our total species dataset (72.4%; across all tested clades the lowest percentage coverage for body size data was for Artiodactyla at 60.7% and the highest was for Lagomorpha at 95.2%; see Table S1 and Supplementary Note 2: Body Size Patterns Through Time for additional clades). These measurements were obtained from the PBDB and collected from the primary literature (see Supplementary Data 2 for a bibliography of this primary literature). Body mass estimates were obtained using clade-specific regressions based on the relationship between m1 length or m1 length × width (occlusional area) and body mass in modern mammals^50,70–73^. For Artiodactyla and Perissodactyla, m1 length alone was used, and body mass calculated using the regression ln(mass_m1_) = 1.24 + (3.11 × (ln(length_m1_)))^71^. For all other mammals (excluding Artiodactyla and Perissodactyla), m1 occlusional area was used to calculate body mass using ln(mass_m1_) = 1.81 + (1.827 × (ln(lengthm1 × width_m1_)))^70^. For Multituberculata, mass calculated based on m1 area was then used to calculate a new mass estimate based on skull length with the regression ln(mass_skull length_) = 0.87 + (0.79 × (ln(length_m1_)))^73^. Body mass was then plotted across ALMAs, using species occurrence midpoint age (Fig. 2C). In an attempt to mitigate sampling bias across time bins, Good’s *u* was calculated for each time bin, based only on the species with body size. Using these Good’s *u* estimates of coverage, unique species with body size per time bin were randomly sampled to the lowest coverage level across all bins to standardize sampling coverage to an extent. This standardization was conducted 1000 times for each clade, and mean, minimum and maximum values were calculated (10 example iterations are shown in Fig. S4 and the mean of 1000 iterations is shown as box plots in Fig. S5). For total Mammalia and all clade datasets, as well as one example bootstrap replica of the body size standardization for total Mammalia, Welch’s two sample *t* tests were performed between adjacent bins to determine whether changes in body mass through time were statistically significant (see Supplementary Data 1).

### Climatic model simulations

Precipitation and potential evaporation output was extracted from published climate model simulations (available at https://www.paleo.bristol.ac.uk/ummodel/scripts/papers/Valdes_et_al_2021.html) for 56 Ma, 52 Ma, 45 Ma, 30 Ma, 36 Ma, 31 Ma and 26 Ma^42^. The simulations were run with a coupled atmosphere-ocean-vegetation model (HadCM3BL-M2.1aD) with a horizontal resolution of 3.75 × 2.5 (longitude x latitude) degrees ( ~ 300 km x 275 km for the mid-latitudes)^42^ which is a variant of the Hadley Center model HadCM3^74^. Pre-industrial simulations of this model have been extensively tested by comparing them with observational data and other climate model simulations^74^. The model performance is as good or better than that of the other CMIP5 models, especially for the region studied. In order to make the model suitable for palaeosimulations, some modifications were made to the model and solar constant, atmospheric CO_2_ and palaeogeographic reconstructions were adjusted for each simulated time, as described in detail in Valdes et al.^42^. To represent the uncertainty in reconstructing atmospheric *p*CO_2_, the LOESS fit of Foster et al.^43^ (‘CO_2_ Scenario 1’ in Fig. 4B and Fig. S14) was used in addition to a smoother CO_2_ curve based on previous successful palaeosimulations as defined in Valdes et al. ^42^ (‘CO_2_ Scenario 2’ in Fig. 4B). The simulations were evaluated in a bounding box in Central Asia expanding from 30° N – 55° N and 80° E – 120° E, approximating the mammal fossil record (Fig. 1A). Herein, we use the aridity index as defined in the World Atlas of Desertification from the United Nations Environment Programme^75^: AI = prec/*E*_p_^44,75^. Both values for prec (precipitation) and for *E*_p_ (potential evaporation) per grid cell are taken directly from the model output. Potential evaporation is the amount of water which could be evaporated, if not limited by water supply, associated with a certain climate. The aridity index defines drylands as regions with AI < 0.65, including specifically dry-subhumid regions (0.5 ≤AI < 0.65), semi-arid regions (0.2 ≤AI < 0.5), arid regions (0.05 ≤ AI < 0.2), and hyper-arid regions (AI < 0.05). The climate model output was analyzed using Python version 3.13.5 and the geoutils package.

### Aridity Index (AI) distributions for Perissodactyla, Rodentia and Glires

To assess whether Perissodactyla and Rodentia (or Glires) may have occupied different climate niches during the Paleocene, Eocene and Oligocene in our study region, we compared the aridity index range for the three clades, respectively. Occurrence-level data for Perissodactyla, Rodentia and Glires were used, and the modern latitude and longitude of each occurrence locality was recorded. The climate model data was available on a different time interval scheme than our occurrence data, because climate models were run for points in time^42^. Within our study interval these were 66 Ma, 61 Ma, 56 Ma, 52 Ma, 45 Ma, 40 Ma, 36 Ma, 31 Ma and 26 Ma. Occurrence ages were matched to climate estimates by creating ‘climate time bins’ whereby the midpoint of each consecutive bin was the point age of the climate model estimate. The midpoint age of the fossil occurrence was then used to assign occurrences to these ‘climate time intervals’. For all occurrences in each clade (Fig. S18), as well as across all time intervals (Fig. S19), the spread of aridity index (AI) values (using both the Foster [‘CO_2_ Scenario 1’^43^] and Smooth [‘CO_2_ Scenario 2’^42^] curves) for Perissodactyla was compared to both Rodentia (Figs. S18A, B and S19A, B) and Glires (Figs. S18C, D and S19C, D). We then performed Welch’s two sample *t* tests between results for Perissodactyla and each of the other two clades (Table S3).

### Reporting summary

Further information on research design is available in the Nature Portfolio Reporting Summary linked to this article.

## Supplementary information

Supplementary Information

Description of Additional Supplementary Files

Supplementary Data 1

Supplementary Data 2

Supplementary Data 3

Supplementary Data 4

Supplementary Data 5

Supplementary Data 6

Supplementary Data 7

Supplementary Data 8

Supplementary Code 1

Reporting Summary

Transparent Peer Review file

## Data Availability

The data generated and used in this study are provided in the Supplementary Information. Fossil mammal occurrence data was sourced from the Palaeobiology Database (PBDB; https://paleobiodb.org retrieved on 10.11.2022)) and the NOW Database (The NOW Community [2026]. New and Old Worlds Database of Fossil Mammals [NOW] and collected from the primary literature. Licensed under CC BY 4.0. Retrieved 12.04.2022 from https://nowdatabase.org/now/database/ [https://doi.org/10.5281/zenodo.4268068]). Mammal measurement data was sourced from the PBDB and collected from the primary literature. Climate model simulations are from Valdes et al.^42^ (accessed via https://www.paleo.bristol.ac.uk/ummodel/scripts/papers/Valdes_et_al_2021.html). Palaeoenvironmental proxy data is from Westerhold et al.^17^ (global benthic foraminifera δ^18^O data), Fang et al.^37^ (weathering intensity in the Qaidam and Xining Basins) and Meijer et al.^35^ (pollen ecotypes through time in the Xining Basin).

## Code availability

The code generated and used in this study is provided in Supplementary Code 1.
