# Supplementary material for: Global and regional climate change impacted Paleogene mammal faunas in Central Asia
Source: Nat Commun. 2026 Sep 8;17:9606. doi: 10.1038/s41467-026-77374-7 (PMC13554191; doi:10.1038/s41467-026-77374-7)
Supplement: Supplementary file 1 [file 41467_2026_77374_MOESM12_ESM.pdf]

nature portfolio

Corresponding author(s): Gemma Louise Benevento

Last updated by author(s): Dec 23, 2025

Reporting Summary

Nature Portfolio wishes to improve the reproducibility of the work that we publish. This form provides structure for consistency and transparency in reporting. For further information on Nature Portfolio policies, see our Editorial Policies and the Editorial Policy Checklist.

Statistics

For all statistical analyses, confirm that the following items are present in the figure legend, table legend, main text, or Methods section.

n/a	Confirmed	
☐	☒	The exact sample size (*n*) for each experimental group/condition, given as a discrete number and unit of measurement
☐	☒	A statement on whether measurements were taken from distinct samples or whether the same sample was measured repeatedly
☐	☒	The statistical test(s) used AND whether they are one- or two-sided<br>*Only common tests should be described solely by name; describe more complex techniques in the Methods section.*
☒	☐	A description of all covariates tested
☒	☐	A description of any assumptions or corrections, such as tests of normality and adjustment for multiple comparisons
☐	☒	A full description of the statistical parameters including central tendency (e.g. means) or other basic estimates (e.g. regression coefficient) AND variation (e.g. standard deviation) or associated estimates of uncertainty (e.g. confidence intervals)
☐	☒	For null hypothesis testing, the test statistic (e.g. *F*, *t*, *r*) with confidence intervals, effect sizes, degrees of freedom and *P* value noted<br>*Give P values as exact values whenever suitable.*
☒	☐	For Bayesian analysis, information on the choice of priors and Markov chain Monte Carlo settings
☒	☐	For hierarchical and complex designs, identification of the appropriate level for tests and full reporting of outcomes
☒	☐	Estimates of effect sizes (e.g. Cohen's *d*, Pearson's *r*), indicating how they were calculated

Our web collection on statistics for biologists contains articles on many of the points above.

Software and code

Policy information about availability of computer code

Data collection	n/a
Data analysis	Analyses were conducted in R (version 4.4.2) and Python (version 3.13.5). Code allowing for the replication of these analyses is provided in the Supplementary Materials. Mammal richness analyses were conducted using R package iNext.

For manuscripts utilizing custom algorithms or software that are central to the research but not yet described in published literature, software must be made available to editors and reviewers. We strongly encourage code deposition in a community repository (e.g. GitHub). See the Nature Portfolio guidelines for submitting code & software for further information.

Data

Policy information about availability of data

All manuscripts must include a data availability statement. This statement should provide the following information, where applicable:

- Accession codes, unique identifiers, or web links for publicly available datasets
- A description of any restrictions on data availability
- For clinical datasets or third party data, please ensure that the statement adheres to our policy

Data Availability. The data generated and used in this study are provided in the Supplementary Information. A large proportion of the data used in this study can be accessed via the Palaeobiology Database (PBDB; https://paleobiodb.org) and the NOW Database [The NOW Community [2026]. New and Old Worlds Database of

Fossil Mammals (NOW). Licensed under CC BY 4.0. Retrieved [download date] from https://nowdatabase.org/now/database/ (doi:10.5281/zenodo.4268068)].
Code Availability: The code generated and used in this study are is provided in the Supplementary Information.

Research involving human participants, their data, or biological material

Policy information about studies with human participants or human data. See also policy information about sex, gender (identity/presentation), and sexual orientation and race, ethnicity and racism.

Reporting on sex and gender	n/a
Reporting on race, ethnicity, or other socially relevant groupings	n/a
Population characteristics	n/a
Recruitment	n/a
Ethics oversight	n/a

Note that full information on the approval of the study protocol must also be provided in the manuscript.

Field-specific reporting

Please select the one below that is the best fit for your research. If you are not sure, read the appropriate sections before making your selection.

☐ Life sciences ☐ Behavioural & social sciences ☒ Ecological, evolutionary & environmental sciences

For a reference copy of the document with all sections, see nature.com/documents/nr-reporting-summary-flat.pdf

Ecological, evolutionary & environmental sciences study design

All studies must disclose on these points even when the disclosure is negative.

Study description	We reconstruct sampling-corrected taxonomic richness, faunal turnover, and body size change for all mammal clades throughout the Paleogene of Central Asia. We combine this data with climate models and investigate whether changing climate may have influenced changes in mammal assemblages through time.
Research sample	Mammalia from the Paleocene, Eocene and Oligocene of Mongolia and Northern China (above 30 degrees North). Chosen because we were interested in patterns of taxonomic diversity, turnover, and body mass from this region during this time period.
Sampling strategy	Data was sampled exhaustively for our time period/region - to the best of our ability.
Data collection	Mammal occurrence data from 66-23 Ma was downloaded for our study region primarily from the PBDB as well as from the NOW Fossil Mammal Database. Datasets were merged and cleaned by hand (duplicates removed, ages checked, etc). Missing data was added to the dataset by the first author, via a literature search. All occurrence data was recorded in excel spreadsheets. Fossil body mass data was also collected where possible for species in our occurrence dataset. All body mass data was recorded in an excel spreadsheet.
Timing and spatial scale	Deep time study. Data spans from 66 million years ago to 23 million years ago.
Data exclusions	Fossil occurrence data was excluded if 1. the geological age could not be dated to a small enough window to make the data usable in this study, or 2. if fossil occurrences were unable to be identified to the species or genus level (dependant on the analysis). For analyses of body mass, any species without a reliable estimate of body mass was excluded.
Reproducibility	All data and code are provided in the Supplementary Materials. The Methods section details analyses performed.
Randomization	n/a - no data was collected or analysed in a way that would require randomization.
Blinding	n/a - no data was collected or analysed in a way that would require blinding.

Did the study involve field work? ☐ Yes ☒ No

Reporting for specific materials, systems and methods

We require information from authors about some types of materials, experimental systems and methods used in many studies. Here, indicate whether each material, system or method listed is relevant to your study. If you are not sure if a list item applies to your research, read the appropriate section before selecting a response.

Materials & experimental systems

n/a	Involved in the study
☒	☐ Antibodies
☒	☐ Eukaryotic cell lines
☐	☒ Palaeontology and archaeology
☒	☐ Animals and other organisms
☒	☐ Clinical data
☒	☐ Dual use research of concern
☒	☐ Plants

Methods

n/a	Involved in the study
☒	☐ ChIP-seq
☒	☐ Flow cytometry
☒	☐ MRI-based neuroimaging

Palaeontology and Archaeology

Specimen provenance: n/a - occurrence datasets created from already published peer-reviewed articles on said fossils.

Specimen deposition: n/a - all fossils included in our occurrence list are already published in peer-reviewed articles.

Dating methods: n/a - all fossils included in our occurrence list have been dated in published peer-reviewed articles.

☐ Tick this box to confirm that the raw and calibrated dates are available in the paper or in Supplementary Information.

Ethics oversight: n/a - no ethical approval or guidance was required. All data used in this study is already published in peer-reviewed articles.

Note that full information on the approval of the study protocol must also be provided in the manuscript.

Plants

Seed stocks: n/a

Novel plant genotypes: n/a

Authentication: n/a
