# Supplementary material for: Global and regional climate change impacted Paleogene mammal faunas in Central Asia
Source: Nat Commun. 2026 Sep 8;17:9606. doi: 10.1038/s41467-026-77374-7 (PMC13554191; doi:10.1038/s41467-026-77374-7)
Supplement: Supplementary file 2 [file 41467_2026_77374_MOESM13_ESM.pdf]

nature portfolio

Peer Review File

Global and regional climate change impacted Paleogene mammal faunas in Central Asia

Corresponding Author: Dr Gemma Benevento

This file contains all reviewer reports in order by version, followed by all author rebuttals in order by version.

Version 0:

Reviewer comments:

Reviewer #1

(Remarks to the Author)
Comments on "Global regional climate change impacted 1 Paleogene mammal faunas in Central Asia" by Benevento et al.
The study identifies three major faunal shifts in Central Asia during the Paleogene: at the Paleocene–Eocene boundary, in the mid-Eocene, at the Eocene–Oligocene Transition (EOT). A key finding is that the mid-Eocene aridification event had a profound impact on mammal faunas, comparable in magnitude to the global PETM EOT events. The work challenges the traditional view of the "Mongolian Remodelling" as a sudden EOT-driven replacement of perissodactyls by rodents, instead attributing much of this pattern to mid-Eocene turnover sampling biases. The integration of sampling-standardized diversity analyses, climate model re-analysis, body-size evolution is a strength, producing a robust, multi-angle reconstruction of faunal dynamics.
In general, I think the work advances our understanding of regional vs. global drivers of faunal change, showing that local aridification events were as important as global temperature shifts. It refines partly overturns long-standing interpretations in the literature (e.g., Meng & McKenna 1998 on the Mongolian Remodelling). The study is based on robust dataset available to my knowledge original in its combined use of sampling-corrected data climate modeling, making it more rigorous than earlier raw-data–based analyses.
The claims about mid-Eocene aridification driving mammal turnover are convincingly backed by both fossil diversity trends climate simulations, the reinterpretation of the Mongolian Remodelling is well-argued, though readers might appreciate a fuller discussion of alternative explanations for the faunal shifts.
Additional fossil sampling in the poorly represented Irdinmanhan ALMA would strengthen temporal resolution, but this is a limitation of the record, not of the analysis. The main deficiency of the work to me is that the authors missed at last two relevant papers the conclusions made therein:
1. Sun, J., Ni, X., Bi, S., Wu, W., Ye, J., Meng, Windley, B.F., 2014. Synchronous turnover of flora, fauna climate at the Eocene–Oligocene Boundary in Asia. Scientific reports, 4(1), p.7463.
2. Bai, B., Meng, J., Janis, C.M., Zhang, Z.Q. Wang, Y.Q., 2020. Perissodactyl diversities responses to climate changes as reflected by dental homogeneity during the Cenozoic in Asia. Ecology Evolution, 10(13), pp.6333-6355.
The first paper was based on data of magnetostratigraphy, pollen climatic proxies from a single section, perhaps the best in the Asian interior. The second paper reached a conclusion that the most conspicuous decrease of perissodactyl diversity that occurred in the latest middle Eocene rather than at the Eocene–Oligocene transition in Asia, as conventionally, so that the authors postulated the hypothesis of the "Ulan Gochu Decline", which was thought to be likely comparable to the contemporaneous post-Uintan decline of the North American land fauna is therefore consistent with the conclusion reached by the authors of the current manuscript. It seems to me unavoidable to incorporate the two papers (and their conclusions) in the discussion of the study by Benevento et al.
The use of Shareholder Quorum Subsampling (SQS) RAC methods is appropriate robust, with multiple sensitivity tests. The dataset is extensive, carefully curated, includes cross-checks with PBDB NOW. Statistical corrections for sampling biases are appropriate represent best practice in paleodiversity analysis. Climate model use (HadCM3) is justified appropriately re-analyzed. The study meets even exceeds expected standards for macroevolutionary paleoclimate integration.
The conclusion that rodents were not necessarily better adapted to arid conditions could be discussed more cautiously, since ecological adaptations are complex not easily inferred from proportional diversity. The narrative (line 319): "Therefore, we do not find a pattern of replacement of perissodactyls by rodents" appears a bit stretching the evidence. I'd suggest the authors rephrase this statement because it is inconsistent with the narrative (lines 320-324): "a turnover from more perissodactyl-dominated to more rodent-dominated mammal faunas", the latter was what Meng & MeKenna (1998) proposed.

The authors occasionally generalize beyond the data (e.g., implications for global faunal evolution). These sections could be tempered. But none of these issues prohibit publication but warrant minor to moderate revision.
The work is strong publication-worthy. The methods are described in detail, with supplementary information covering data cleaning, SQS, RAC, body-mass estimation.
In addition to what I said above, revisions are needed mainly to tighten interpretations refine some sections of English. Some sentences are overly long complex (e.g., Introduction lines 65–75, Discussion lines 228–245). Shortening would improve readability. Minor grammatical issues: inconsistent tense use, occasional missing articles ("the PETM was…", "in Central Asia the fauna…"). Phrasing such as "our results suggest that…" seems overused; varying expression would strengthen style.

(Remarks on code availability)
N/A

Reviewer #2

(Remarks to the Author)
The authors examine mammalian diversity in response to environmental changes during the Paleogene of central Asia. The authors capture mammalian diversity through sampling-corrected taxonomic richness, faunal turnover, body size change. The authors identify a major mammalian faunal turnover during the Eocene of central Asia, in addition to those at the PETM EOT. This turnover involved a decrease in mammal diversity, linked to continental drying cooling, rather than global climate change as in the PETM EOT faunal transitions.

Noteworthy results include the findings that:
-the Mongolian Remodeling event, when considered from sampling-corrected generic richness, is less dramatic than when considering raw richness only, may exist as a result of sampling biases.
-There is no evidence of replacement of perissodactyls by rodents during the Mongolian Remodeling.

This manuscript is generally well written, presents a unique take on a question that many readers interested in Cenozoic mammalian paleobiology will find interesting relevant. The authors use innovative methods to accomplish their aims. As far as I am aware, this work represents a novel contribution. The figures are well-conceived.

I have several concerns about this manuscript that I will detail below. More in-depth explanation is provided in the line edits in the in-text notes below.

1.The names of taxonomic orders need to be capitalized when referring to them as a proper noun. Other small grammatical or stylistic changes are noted in the text.
2.The number of figures, supplemental figures, data frames, supplementary materials make this manuscript difficult to read follow. Some figures included in the supplementary should rather be included in the main text, as the manuscript is difficult to parse without them. Figure S1, for example, should be included in the main text (or a justification should be provided for why generic richness is more relevant to these analyses than species richness). The authors may want to consider narrowing down to only a few key supplementary figures which are needed to understand the main text.
3.Some analyses their usage need to be justified, see in-text edits. Some statements need to be supported by evidence

Summary of major in-text notes:
-Glires is a proper noun should be capitalized throughout. My understanding is that Glires cannot be used in the singular (ex., the authors use "glire" throughout).
-The plural form of "genus" is "generic", genus richness/turnover/etc. should be changed to generic throughout.
-There are small grammatical mistakes throughout which should be addressed.
- https://www.nature.com/ncomms/submit/article The journal guidelines for articles states that the Discussion should be succinct may not contain subheadings. There are currently subheadings included in the Discussion of this manuscript.
-Use more precision in references to "diversity" or "richness", each should be accompanied by either "generic" or "species" because your article deals with both.
-Please justify the comparison of body mass estimates which were calculated using different regression equations. Each equation introduces a varying amount of error can likely not be compared across different regressions.
-Expand on how sampling-standardized richness was calculated justify the use of this method.
-Justify the randomization technique used to assign a single time interval to each fossil.

In closing, my recommendation is that revisions are required for this manuscript.

(Remarks on code availability)

Reviewer #3

(Remarks to the Author)
In this manuscript, Benevento et al. evaluate mammalian turnover patterns in central Asia through the Paleogene. They specifically reevaluate the "Mongolian Remodeling" of previous authors who showed that perissodactyls dominated early

Paleogene faunas rodents dominated late Paleogene faunas. The authors analyze sampling-standardized richness body-mass estimates to reconstruct turnover body-size changes in mammalian assemblages through the Paleogene. In addition, they model the aridity index (Ai) of the region to see when drying occurred in central Asia whether turnover or body mass changed in relation to drying cooling climate.

The climate modeling results show that arid conditions expanded across the study area throughout the Paleogene was not confined to the Eocene–Oligocene transition (EOT). Significant turnover in mammalian clades occurs in the early Eocene is driven by the loss of archaic clades groups other than perissodactyls glires. While glires constitute greater proportions of faunas following the EOT, perissodactyl abundance richness do not decline significantly, showing that turnover patterns proposed by the classic Mongolian Remodeling is not supported by this analysis.

I think these analyses are well done involve an important data set, given the tectonic, geographic, climate setting of the Paleogene in central Asia. I think this work is appropriate for the journal, with minor revisions to improve clarity to more fully discuss some of the features of the central Asian fossil record.
The authors do their statistical due diligence are careful to describe the statistical analyses applied to the taxonomic data in this work, especially given the high variability in number of occurrences through the study interval. These analyses appear very thorough justified. I would, however, appreciate more explanation of how the climate models/aridity index output were produced, including how the representative Ai results shown in Fig. 1 were produced.

One aspect of the fossil record of central Asia that I find interesting is a significant decrease in occurrences during the mid Eocene. The authors treat this well in their statistical analyses, but their only comment on the occurrence data is that it may be the result of “sampling bias.” I encourage the authors to view this as the record that drives patterns of occurrence, which may relate to the large-scale changes in climate geography in central Asia during the Paleogene. I think that more thought discussion about the influence of common-causal mechanisms on turnover patterns would help flesh out the reasons for observed turnover gap in the record of the mid Eocene. Including more discussion of this topic could also increase the reach of this manuscript beyond those working on the Asian fossil record.

Specific comments

Line 123 elsewhere: Perhaps “richness” is a better term than diversity if the number of taxa are used in the analyses, rather than a different “diversity” metric.
Beginning line 212: Move the climate model analysis section earlier in the Results. These results are shown in Fig. 1, which is cited early in the paper, but they are not discussed until after the results of the faunal analyses shown in later figures. Moving the section earlier will preserve the flow of the results as shown by the series of figures.

Line 220: Is an Ai value of 0.2 the cut off for “desert-like conditions”? Is this cutoff based on modern climate? Consider adding this information to the caption for Fig. 1.

Line 221: What does “greener” mean in this sentence? Were plants less susceptible to dry conditions because of higher water-use efficiency, therefore able to support larger leaves than modern dry-adapted plants? Or were temperate plants able to inhabit otherwise drier environments? Would the implication that Eocene biomes don’t map onto modern aridity indices affect the interpretation of “arid” conditions in the climate models?

Line 305: Is it “sampling bias,” or is there very little available to sample? This would imply a larger-scale influence of the rock record on patterns in the fossil record during the mid Eocene (see more below).

Lines 305–306: “more extensive record of sedimentary rocks . . .” Here, "extensive" implies greater areal coverage of fossiliferous formations rather than total number of fossiliferous formations, which is what is shown in Fig. S21. I recommend rephrasing this as "increased number of fossiliferous formations" or similar.
I think this point deserves more discussion than one parenthetical. It could well be that some formations localities are screen washed for small mammals more extensively than others, which would increase the richness of glires from those sites. If formations from this interval are underexplored for fossils, then it could be worth calling it to attention to inspire more work on the mid Eocene. If formations from this interval have been explored very few yield fossils, then that indicates that larger-scale mechanisms could be affecting fossil preservation the patterns of occurrence.

Lines 322–323: The middle Eocene shows a drastic decrease in number of fossiliferous formations associated decrease in generic richness; this coincides with the loss of other mammal clades, which drives the turnover pattern. The authors may include this in the "sampling bias" they mention in line 305 earlier, but it is worth specifically mentioning the influence of the loss of the record on the data analyses.

Very few fossil localities occur during the interval when it is thought that increased weathering of the Himalaya drew down CO2 led to mid–late Eocene cooling cooling at the EOT. Could the coincidence of changes in weathering, climate, the deposition of fossil-bearing formations point to a common causal mechanism that shapes the rock fossil records (Peters et al., 2022)? This would not preclude true turnover of clades during this interval, but the loss of the record (from nondeposition or erosion) accentuates the turnover patterns. I think this is an interesting important part of the data shown here deserves more discussion.

Line 340: Are the geological records mentioned here the weathering intensity proxies shown in Fig. 4? If so, cite the reference here.

Line 359: Specify total latitudinal range, since no occurrences from Russia were analyzed. So, 30–50° to match Fig. 1a?

Line 362: Cite the primary literature from which additional occurrences were obtained, or include reference list in supplemental material.

Lines 363–365: Mention the number of localities as well as taxon occurrences genera. It would also be useful to know the number of localities per ALMA, perhaps shown as a table in the supplemental material.

Lines 369–371: Was the age bootstrapping done for each fossil occurrence or each taxon occurrence? Perhaps specify "taxon" instead of "fossil" in lines 370 371, as "fossil" to me means each element recovered from a locality.

Line 416: Cite the primary literature from which body-mass data was collected, or include reference list in supplemental material.

Line 431: Simulations for these time slices do not seem to be published in the Valdes et al. reference given. I did not thoroughly read this reference, but it appears to be more of a newsletter than a research article. The article in Valdes et al. that relates to Eocene climate does contain links to paleo-climate data sources, but more information is needed about where these published simulations occur. The authors should check this citation or provide more information about where these simulations were published or how the simulations were accessed.

Lines 439–442: Do both CO2 scenarios come from Foster et al.? This reference should be cited earlier as the source of the two CO2 curves. Also, what are the criteria for a "successful" simulation? Did the model closely match climate proxy data, or did it simply produce output? Add additional information to increase clarity.

Line 629: How were the "representative" Ai maps produced? Does this output represent a combination or average of the two CO2 scenarios? I don't recall seeing any information in the Methods or supplemental material about how the representative models were produced.

Line 675: Cite the original source(s) of the CO2 scenarios.

Tables figures

The figures are all well designed clearly show the results.

Figure 4: In b) is the dotted line at Ai=0.2 meant to show the cutoff for desert-like conditions? If so, please add a line in the figure caption to explain.

Supplemental material

I know that the formatting design of information figures in supplemental information does not require the same degree of scrutiny as the manuscript text figures; however, I feel that some minor changes would improve clarity for readers who seek out the supplemental files.

Page 7: The description of geographic area sampling-standardized diversity ends mid-sentence.

Table S2: Consider reducing the significant digits of the percentage of taxa with body-mass data. This will increase legibility, this degree of precision is not necessary.

Figure S18: It seems that Fig. S15 is incorrectly referenced here. Is the caption meant to refer to Fig. S17?

Table S21: Consider using dashed lines instead of dotted lines. Dotted lines are unusual in line graphs because dots could be mistaken for data points.

References
Peters, S.E., Quinn, D.P., Husson, J.M., Gaines, R.R., 2022, Insights into cyclic secular evolution of the Earth-life system: Annual Reviews in Earth Planetary Science, v. 50, 419–449.

(Remarks on code availability)
The code files appear to be the raw inputs to R as used by the authors. Many data file names file locations are retained, it is not always clear how these files translate to the data files (.xlsx) provided as supplemental info (and some do not seem to be provided). Some light cleaning of the code could improve useability, or a README file could help translate between the appropriate data file the code implementation.

Version 1:

Reviewer comments:

Reviewer #1

(Remarks to the Author)
I appreciate the authors have provided a thorough constructive response to my comments have implemented substantial revisions that strengthen the manuscript. They express appreciation for the reviewers' comments show a genuine commitment to improving clarity, rigor, balance of their manuscript. The revised version reflects significant textual edits, structural improvements, methodological enhancements.
I'm glad the authors carefully addressed substantive scientific critiques/comments. They incorporated previously omitted but highly relevant studies by Sun et al. (2014) Bai et al. (2020), at least in my view, integrating their conclusions into the discussion clarifying points of agreement methodological differences. Interpretations were appropriately tempered where requested, including the discussion of ecological adaptation in lagomorphs rodents, claims regarding faunal turnover at the Eocene–Oligocene Transition were refined for precision consistency with prior work. Overgeneralized statements about global implications were removed, language throughout the manuscript was tightened to improve readability, reduce redundancy, correct grammatical inconsistencies.
Overall, the revisions are comprehensive, scientifically sound, responsive. The authors engaged constructively with both conceptual stylistic critiques, resulting in a clearer, more balanced, methodologically robust manuscript that meets high professional standards. I have no additional comment look forward to seeing the published work.

(Remarks on code availability)

Reviewer #2

(Remarks to the Author)
I thank the authors for making the suggested revisions, for offering explanations for why some revisions were not made. I am satisfied that the concerns raised in my first review have been met. I have included some small in-text edits that I would recommend addressing, but besides this, I recommend publication.

(Remarks on code availability)

Reviewer #3

(Remarks to the Author)
This is a revised submission from Benevento et al. on the turnover of mammalian clades in central Asia during the Paleogene. The authors have done a good thorough job responding to the reviewer comments suggestions. The manuscript thoughtfully deals with an important fossil record from an important interval of central Asian history, I think it is ready for acceptance.

The authors have adequately addressed my comments on the original submission. My comments on the original submission largely pertained to clarifying how the aridity model was produced where the climate data came from. The authors have addressed this comment in the text by adding additional reference information in the supplemental files. I also commented that the poor fossil record from the Irdinmanhan LMA could be related to larger climatic or tectonic drivers merited more direct discussion (although I recognize that this is not the main aim of the manuscript). The authors now explicitly address this part of the record how it may affect their results, which I think helps to round out the discussion of the large-scale turnover patterns.

I also suggested that the authors modify their code files to be easier to follow link to the appropriate datasets. They have supplied an example of the revised code files (revising code is time-consuming, I do not fault them for not including all files at this stage). This file is much easier to read use, I take this as a good model for the additional files in the finalized submission.

I have only minor typographical comments on the revision, listed below.

Best,
Katharine Loughney

Specific comments

Lines 45 49: Use EM dashes to separate the clauses, not EN dashes.

Line 70: Need a comma at the end of the phrase beginning "a key period in mammal evolution . . ."

Lines 97 98: Use EM dashes to set off the clause after "Lagomorpha," not hyphens.

Line 679: A typo—Asian LMA, not American.

Line 688: Can omit "(Ai)" since it appears at line 682.

Supplemental material

Figure S14: Consider adding a sentence to the caption explaining the dashed lines in the figure.

(Remarks on code availability)
I have reviewed the example revised code, it is much more user-friendly than the original files. I thank the authors for taking the time to modify their code.

Open Access This Peer Review File is licensed under a Creative Commons Attribution 4.0 International License, which permits use, sharing, adaptation, distribution and reproduction in any medium or format, as long as you give appropriate credit to the original author(s) and the source, provide a link to the Creative Commons license, and indicate if changes were made.
In cases where reviewers are anonymous, credit should be given to 'Anonymous Referee' and the source.
The images or other third party material in this Peer Review File are included in the article's Creative Commons license, unless indicated otherwise in a credit line to the material. If material is not included in the article's Creative Commons license and your intended use is not permitted by statutory regulation or exceeds the permitted use, you will need to obtain permission directly from the copyright holder.
To view a copy of this license, visit https://creativecommons.org/licenses/by/4.0/

Dear Reviewers,

Thank you very much for your fair and considered reviews of our research article. We have made an effort to incorporate or address all your suggestions and concerns and detail these additions below. We sincerely feel that our manuscript is strengthened as a result of these changes, and very much appreciate your time and thoughtful comments.

In summary, we have (i) made significant edits and additions to the text and to specific wording in line with all reviewer comments (and in some cases in line with NC guidelines – e.g. shortening the abstract), (ii) moved some information from the supplement to the main text (e.g. Table 1), (iii) performed an additional statistical test to test whether different body mass estimates could be changing results, (iv) cited new literature, (v) overhauled the supplementary figures to reduce the number of panels, and streamlined and edited Appendix S1 to make it more reader-friendly – including adding a table of contents, (vi) added a bibliography of primary literature to Appendix S1, and (vii) made the code more user-friendly.

We have implemented almost all reviewer suggestions, and for the very few instances where we may not have done exactly as suggested, we have either found an alternative option or given a clear explanation of why we may not be able to implement the change.

Due to the high volume of changes to the text, we have provided both a track-changes and a clean version of the manuscript and supplement. Line numbers refer to the clean version of the manuscript.

Reviewer #1

The study identifies three major faunal shifts in Central Asia during the Paleogene: at the Paleocene–Eocene boundary, in the mid-Eocene, and at the Eocene–Oligocene Transition (EOT). A key finding is that the mid-Eocene aridification event had a profound impact on mammal faunas, comparable in magnitude to the global PETM and EOT events. The work challenges the traditional view of the “Mongolian Remodelling” as a sudden EOT-driven replacement of perissodactyls by rodents, instead attributing much of this pattern to mid-Eocene turnover and sampling biases. The integration of sampling-standardized diversity analyses, climate model re-analysis, and body-size evolution is a strength, producing a robust, multi-angle reconstruction of faunal dynamics.

In general, I think the work advances our understanding of regional vs. global drivers of faunal change, showing that local aridification events were as important as global temperature shifts. It refines and partly overturns long-standing interpretations in the literature (e.g., Meng & McKenna 1998 on the Mongolian Remodelling). The study is based on robust dataset available to my knowledge and original in its combined use of sampling-corrected data and climate modeling, making it more rigorous than earlier raw-data–based analyses.

The claims about mid-Eocene aridification driving mammal turnover are convincingly backed by both fossil diversity trends and climate simulations, and the reinterpretation of the Mongolian Remodelling is well-argued, though readers might appreciate a fuller discussion of alternative explanations for the faunal shifts. Additional fossil sampling in the poorly represented Irdinmanhan ALMA would strengthen temporal resolution, but this is a limitation of the record, not of the analysis.

Author response: Thank you for the praise of our work and insights! We have extended the discussion slightly in scope to incorporate more alternative explanations, also in response to reviewer 3 (see below).

The main deficiency of the work to me is that the authors missed at last two relevant papers and the conclusions made therein:

1. Sun, J., Ni, X., Bi, S., Wu, W., Ye, J., Meng, J. and Windley, B.F., 2014. Synchronous turnover of flora, fauna and climate at the Eocene–Oligocene Boundary in Asia. Scientific reports, 4(1), p.7463.

2. Bai, B., Meng, J., Janis, C.M., Zhang, Z.Q. and Wang, Y.Q., 2020. Perissodactyl diversities and responses to climate changes as reflected by dental homogeneity during the Cenozoic in Asia. Ecology and Evolution, 10(13), pp.6333-6355.

The first paper was based on data of magnetostratigraphy, pollen and climatic proxies from a single section, perhaps the best in the Asian interior. The second paper reached a conclusion that the most conspicuous decrease of perissodactyl diversity that occurred in the latest middle Eocene rather than at the Eocene–Oligocene transition in Asia, as conventionally, so that the authors postulated the hypothesis of the “Ulan Gochu Decline”, which was thought to be likely comparable to the contemporaneous post-Uintan decline of the North American land fauna and is therefore consistent with the conclusion reached by the authors of the current manuscript. It seems to me unavoidable to incorporate the two papers (and their conclusions) in the discussion of the study by Benevento et al.

Author response: Thank you for mentioning these papers. We agree that we should have cited them and now do so.

For the first (Sun et al. 2014), we now reference this multiple times when discussing the EOT and perissodactyl/rodent turnover (e.g. lines 94-95 & 101-102).

The second paper is indeed very relevant. In fact, we had originally included their conclusions in our discussion, but over time across the versions it appears to have been completely removed. We found it difficult to marry our results with theirs because they are using the raw fossil record, and it is difficult to know how this should be interpreted, but we should have tried harder to include their conclusions, as they do mirror ours to some extent. We have now included the following sentences:

Lines 284-287: *"Previous analyses of Perissodactyla in this region found both the first occurrence of mesodont species and a decrease in raw (uncorrected) species richness immediately following this time interval and prior to the EOT, mirroring our results for total Mammalia species richness and further suggesting a protracted period of recovery [54]."*

Lines 341-343: *"These results mirror a previously recovered decline in raw (uncorrected) species richness in Perissodactyla millions of years prior to the EOT boundary during the Ulangochuian, named the Ulan Gochu Decline [54]."*

The use of Shareholder Quorum Subsampling (SQS) and RAC methods is appropriate and robust, with multiple sensitivity tests. The dataset is extensive, carefully curated, and includes cross-checks with PBDB and NOW. Statistical corrections for sampling biases are appropriate and represent best practice in paleodiversity analysis. Climate model use (HadCM3) is justified and appropriately re-analyzed. The study meets and even exceeds expected standards for macroevolutionary and paleoclimate integration.

Author response: Thank you!

The conclusion that lagomorphs were not necessarily better adapted to arid conditions could be discussed more cautiously, since ecological adaptations are complex and not easily inferred from proportional diversity.

Author response: Yes, this is very true. We tempered our sentence by editing this paragraph as follows (lines 324-331): *"Moreover, the previously postulated turnover from Perissodactyla-dominated to Rodentia-dominated faunas has been attributed to Rodentia being better adapted to a drying environment [5]. Within our dataset, however, Perissodactyla are consistently found in localities that are reconstructed as more arid when compared to localities with Glires (statistically significantly lower modelled A_I at locations where Perissodactyla fossils are recovered: Table S3 in Appendix S1; Figs S18 & S19). This pattern suggests a more complex relationship than has previously*

been suggested, and the potential for a stronger arid adaptation of extant Glires compared to extinct 'archaic' clades would be worth investigating in the future."

The narrative (line 319): "Therefore, we do not find a pattern of replacement of perissodactyls by rodents" appears a bit stretching the evidence. I'd suggest the authors rephrase this statement because it is inconsistent with the narrative (lines 320-324): "a turnover from more perissodactyl-dominated to more rodent-dominated mammal faunas", the latter was what Meng & MeKenna (1998) proposed.

Author response: Thank you for noticing this inconsistency. Here, we were referring specifically to a turnover at the EOT. The line (337-339) now reads "*Therefore, we do not find a strong pattern of turnover from more Perissodactyla-dominated to more Rodentia-dominated mammal faunas at the EOT boundary, which has previously been the defining factor of the Mongolian Remodelling [5].*"

The authors occasionally generalize beyond the data (e.g., implications for global faunal evolution). These sections could be tempered. But none of these issues prohibit publication but warrant minor to moderate revision.

Author response: Yes, thank you for your feedback. We have now removed this section completely because not all authors agreed with the addition of this paragraph.

The work is strong and publication-worthy. The methods are described in detail, with supplementary information covering data cleaning, SQS, RAC, and body-mass estimation.

Author response: Thank you!

In addition to what I said above, revisions are needed mainly to tighten interpretations and refine some sections of English. Some sentences are overly long and complex (e.g., Introduction lines 65–75, Discussion lines 228–245). Shortening would improve readability. Minor grammatical issues: inconsistent tense use, occasional missing articles ("the PETM was…", "in Central Asia the fauna…"). Phrasing such as "our results suggest that…" seems overused; varying expression would strengthen style.

Author response: Thank you for pointing this out to us, we amended these throughout.

Reviewer #2

The authors examine mammalian diversity in response to environmental changes during the Paleogene of central Asia. The authors capture mammalian diversity through sampling-corrected taxonomic richness, faunal turnover, and body size change. The authors identify a major mammalian faunal turnover during the Eocene of central Asia, in addition to those at the PETM and EOT. This turnover involved a decrease in mammal diversity, linked to continental drying and cooling, rather than global climate change as in the PETM and EOT faunal transitions.

Noteworthy results include the findings that:
-the Mongolian Remodeling event, when considered from sampling-corrected generic richness, is less dramatic than when considering raw richness only, and may exist as a result of sampling biases.
-There is no evidence of replacement of perissodactyls by rodents during the Mongolian Remodeling.

This manuscript is generally well written, and presents a unique take on a question that many readers interested in Cenozoic mammalian paleobiology will find interesting and relevant. The authors use innovative methods to accomplish their aims. As far as I am aware, this work represents a novel contribution. The figures are well-conceived.

Author response: Thank you!

I have several concerns about this manuscript that I will detail below. More in-depth explanation is provided in the line edits and in the in-text notes below.

1.The names of taxonomic orders need to be capitalized when referring to them as a proper noun. Other small grammatical or stylistic changes are noted in the text.

Author response: Yes, thank you. We have thoroughly checked our manuscript and have implemented all your suggested grammatical changes from here and your in-line comments.

2.The number of figures, supplemental figures, data frames, and supplementary materials make this manuscript difficult to read and follow. Some figures included in the supplementary should rather be included in the main text, as the manuscript is difficult to parse without them. Figure S1, for example, should be included in the main text (or a justification should be provided for why generic richness is more relevant to these

analyses than species richness). The authors may want to consider narrowing down to only a few key supplementary figures which are needed to understand the main text.

Author response: Thank you for your feedback on this. While we really appreciate this and have tried to improve clarity where possible, we would prefer to keep all our supplementary material, as we feel all figures and tables support our main analyses and conclusions. We have combined some figures in the supplement to improve accessibility, and added a table of contents and table and figure lists at the start of the document. We have also included one additional table in the main manuscript (Table 1).

In reference to Figure S1, the species richness curve, we opted to include the number of genera in place of number of species because the number of genera is usually significantly more robust to poor sampling, and much more commonly reported in studies of the mammal fossil record. The decreased robustness is also evident here, because the species richness curve has fewer time intervals (ALMAs) with a coverage-based richness estimate at higher quorums, even resulting in some additional intervals not being estimated at any quorum. Additionally, the turnover results were presented at the genus level (for the same reasons) and therefore are better able to be interpreted together with the genus-level richness results.

Even though the editor advised us that the journal allows a few more graphic elements than we currently include, we have decided to reduce complexity of the main manuscript as much as possible, only presenting the main analyses which we feel are needed by readers to follow the highlighted principal results. The supplementary figures represent mostly results of sensitivity analyses and supplementary information for readers who are experts in the mammal fossil record or in climate model simulations. We felt that given the broad scope of the journal, the majority of journal readership would appreciate a more concisely illustrated main text.

3.Some analyses and their usage need to be justified, see in-text edits. Some statements need to be supported by evidence

Author response: We have reviewed the in-line comments and have added justification where needed/indicated. Thank you.

Summary of major in-text notes:
-Glires is a proper noun and should be capitalized throughout. My understanding is that Glires cannot be used in the singular (ex., the authors use “glire” throughout).

Author response: Thank you for catching this. This has been done throughout.

-The plural form of “genus” is “generic”, and genus richness/turnover/etc. should be changed to generic throughout.

Author response: Although we have opted not to implement this exact phrasing in the text, because “generic” has a different meaning in normal English usage (as with “specific” for species), we have instead corrected the term to “richness in genera” or “number of genera”. Thank you for your feedback.

-There are small grammatical mistakes throughout which should be addressed.

Author response: Thank you for pointing this out. We have thoroughly checked the manuscript and hope that we have caught all grammatical inconsistencies.

- https://www.nature.com/ncomms/submit/article The journal guidelines for articles states that the Discussion should be succinct and may not contain subheadings. There are currently subheadings included in the Discussion of this manuscript.

Author response: Thank you. We have now removed all Discussion subheadings.

-Use more precision in references to “diversity” or “richness”, each should be accompanied by either “generic” or “species” because your article deals with both.

Author response: Implemented throughout, as noted above. Thank you for the suggestion!

-Please justify the comparison of body mass estimates which were calculated using different regression equations. Each equation introduces a varying amount of error and can likely not be compared across different regressions.

Author response: As you say, we use different regression equations for different clades. It is true that these equations have different errors associated with them, because some clades have a tighter fit to their regression line than others. While this is the case, each regression will fit its respective clade better than any generic m1 equation for all mammals. Therefore, using a generic regression for all mammals would introduce even more error, and would be less comparable across clades. It is possible that equations for some clades are more prone to over- or underestimation compared to others. However, because we are dealing with multiple clades with a range of body sizes which span multiple orders of magnitude, this is unlikely to significantly skew our data. Any individual clade is unlikely to span the entire range of mammalian body sizes (see Benevento et al. 2023. Palaeontology, Sup Mat Figure S6).

The same reason applies for not using raw m1 length/width measurements. While m1 appears to have a tight relationship to body mass across mammal clades, the precise relationship differs, and therefore the clade-specific regressions are needed to account for these scaling differences across clades.

In addition, we re-ran the pairwise t-tests without the 7 body mass values which were estimated using methods other than m1 regressions. The results remained the same, including the statistically significant body mass changes between bins (see Dataframe S7, sheet "All Mammalia m1 only t-test").

-Expand on how sampling-standardized richness was calculated and justify the use of this method.

Author response: Thank you for the feedback. The methods section on sampling standardization now includes the following (Lines 392-397): "*Sampling-standardized richness was calculated using coverage-based rarefaction and extrapolation in the R package iNext [60,61] (Dataframes S3 & S4). The coverage-based rarefaction and extrapolation method samples time bins to an equal coverage (or quorum) of the underlying taxon pool, based on the taxon frequency distribution[46,47,62]. While no method is capable of perfectly reconstructing true richness, this approach provides one of the best options for estimating taxonomic richness in the fossil record to date [62].*"

-Justify the randomization technique used to assign a single time interval to each fossil.

Author response: Each fossil occurrence or fossil site is given an age range in the literature and/or the databases used herein. These age ranges are estimated using various fossil dating methods, and can be of varying lengths. The longer the estimated age range, the more error is introduced when trying to date a fossil occurrence. This is because we know that any given fossil occurrence represents an organism that lived at only one point in time. This dating error becomes more troublesome when the age range spans more than one of the time intervals that we are using in our analysis. We a-priori removed any fossil occurrences whose age estimate spanned more than 3 time intervals (and additionally more than 2 time intervals for the sensitivity analysis in the supplement). There are then two options to deal with the remaining occurrences which contain an age estimate error that spans more than one of our time intervals. The first is taking the midpoint of this estimated age range and assigning it to the occurrence. This is a valid approach, and is used by many researchers. However, for example, if you have two time intervals, the first, Time Bin A, is 60 – 55 Ma and the second, Time Bin B, is 55 – 35 Ma, and you have a number of occurrences that are dated as Time Bin A or B, the midpoint of this occurrence age estimate will always fall within Time Bin B.

By using a randomization technique, we choose an age at random from the range, and assign the occurrence to that time interval. We repeat this 500 times, and analyse the mean result from these bootstrap replicates. This has the advantage, in our opinion, in allowing the occurrence to sometimes fall within the shorter time interval (Time Bin A). Additionally, the number of times that this occurrence then falls within one of the two bins should be roughly correlated to the amount of the occurrence range that each time interval overlaps with.

This method was established and used in Benevento et al. 2023 (Palaeontology).

We have clarified the text regarding this procedure in the main methods section (Lines 387-392): "*As fossil occurrences are often dated imprecisely, we used a randomization technique to assign a single time interval within a fossil's given age range to the individual fossil for analyses, where a numerical age was drawn at random from each fossil's age distribution multiple times. This was done for each occurrence and then sampling-standardized richness was calculated for each ALMA. The age randomization and richness calculations were repeated 500 times to capture the variation in age estimates, and error bars were produced using 95% CIs from the 500 richness results.*"

Reviewer #3

In this manuscript, Benevento et al. evaluate mammalian turnover patterns in central Asia through the Paleogene. They specifically reevaluate the "Mongolian Remodeling" of previous authors who showed that perissodactyls dominated early Paleogene faunas and rodents dominated late Paleogene faunas. The authors analyze sampling-standardized richness and body-mass estimates to reconstruct turnover and body-size changes in mammalian assemblages through the Paleogene. In addition, they model the aridity index (Ai) of the region to see when drying occurred in central Asia and whether turnover or body mass changed in relation to drying and cooling climate.

The climate modeling results show that arid conditions expanded across the study area throughout the Paleogene and was not confined to the Eocene–Oligocene transition (EOT). Significant turnover in mammalian clades occurs in the early Eocene and is driven by the loss of archaic clades and groups other than perissodactyls and glires. While glires constitute greater proportions of faunas following the EOT, perissodactyl abundance and richness do not decline significantly, showing that turnover patterns proposed by the classic Mongolian Remodeling is not supported by this analysis.

I think these analyses are well done and involve an important data set, given the tectonic, geographic, and climate setting of the Paleogene in central Asia. I think this work is appropriate for the journal, with minor revisions to improve clarity and to more fully discuss some of the features of the central Asian fossil record.

Author response: Thank you!

The authors do their statistical due diligence and are careful to describe the statistical analyses applied to the taxonomic data in this work, especially given the high variability in number of occurrences through the study interval. These analyses appear very thorough and justified. I would, however, appreciate more explanation of how the climate models/aridity index output were produced, including how the representative Ai results shown in Fig. 1 were produced.

Author response: Thank you for your feedback! We now included a more detailed explanation of the aridity index, of the model variables used to calculated it, and of the categories classified by the aridity index in the Methods section and refer to this in the main text.

Lines 157-159: "*Based on the climate model output with a spatial resolution of 3.75° longitude and 2.5° latitude (see Fig. 1b), we calculated the aridity index (A_I) as the quotient of precipitation and potential evaporation (see Methods for a detailed definition).*"

Lines 469-475: "*Herein we use the aridity index as defined in the World atlas of desertification from the United Nations Environment Programme [74]: $A_I = prec/E_p$ [44,74]. Both values for prec (precipitation) and for E_p (potential evaporation) per grid cell are taken directly from the model output. Potential evaporation is the amount of water which could be evaporated, if not limited by water supply, driven by a certain climate. The aridity index defines drylands as regions with $A_I < 0.65$, including specifically dry-subhumid regions ($0.5 < A_I < 0.65$), semi-arid regions ($0.2 < A_I < 0.5$), arid regions ($0.05 < A_I < 0.2$), and hyper-arid regions ($A_I < 0.05$).*"

One aspect of the fossil record of central Asia that I find interesting is a significant decrease in occurrences during the mid Eocene. The authors treat this well in their statistical analyses, but their only comment on the occurrence data is that it may be the result of "sampling bias." I encourage the authors to view this as the record that drives patterns of occurrence, which may relate to the large-scale changes in climate and geography in central Asia during the Paleogene. I think that more thought and discussion about the influence of common-causal mechanisms on turnover patterns would help flesh out the reasons for observed turnover and gap in the record of the mid

Eocene. Including more discussion of this topic could also increase the reach of this manuscript beyond those working on the Asian fossil record.

Author response: We appreciate your point, and have addressed this by expanding our discussion (see more details below in response to your comments on lines 305 and following).

Line 123 and elsewhere: Perhaps “richness” is a better term than diversity if the number of taxa are used in the analyses, rather than a different “diversity” metric.

Author response: Yes, absolutely. Done throughout, thank you.

Beginning line 212: Move the climate model analysis section earlier in the Results. These results are shown in Fig. 1, which is cited early in the paper, but they are not discussed until after the results of the faunal analyses shown in later figures. Moving the section earlier will preserve the flow of the results as shown by the series of figures.

Author response: Done, thank you. These results now appear after our first overarching paragraph in the methods, beginning in line 149, which led to slight further restructuring of the results section and the first paragraph in the discussion.

Line 220: Is an Ai value of 0.2 the cut off for “desert-like conditions”? Is this cutoff based on modern climate? Consider adding this information to the caption for Fig. 1.

Author response: Thank you for the sensible suggestion. We have added the following to Figure 1 figure caption (lines 684-686): “*For reference, an A_I of < 0.65 indicates drylands. More specifically, an A_I of 0.65 to 0.5 indicates dry-subhumid regions, an A_I of 0.5 to 0.2 semi-arid, an A_I of 0.2 to 0.05 arid, and an A_I of < 0.05 hyper-arid regions.*”

Line 221: What does “greener” mean in this sentence? Were plants less susceptible to dry conditions because of higher water-use efficiency, and therefore able to support larger leaves than modern dry-adapted plants? Or were temperate plants able to inhabit otherwise drier environments? Would the implication that Eocene biomes don’t map onto modern aridity indices affect the interpretation of “arid” conditions in the climate models?

Author response: Thank you for this comment. “Greener” was indeed not very clear. We have re-formulated this sentence as “*However, we note that vegetation productivity and cover in a high-CO_2 world, such as the Eocene, may have been somewhat higher than today for a given aridity index, potentially affecting the common interpretation of the*

aridity index as applied here, because plants have a higher water-use efficiency at high atmospheric pCO_2 *[45].*" (lines 164-167).

We would not expect larger leaves, but increased productivity, resulting in larger vegetation cover. Indeed, this affects the interpretation of 'arid' in climate models. We have included the sentence to make this clear. The magnitude of these plant-physiological CO2 effects, however, is highly debated (Walker AP, De Kauwe MG, Bastos A, Belmecheri S, Georgiou K, Keeling RF, et al. Integrating the evidence for a terrestrial carbon sink caused by increasing atmospheric CO2. New Phytologist. 2021;229(5):2413-45.)

Line 305: Is it "sampling bias," or is there very little available to sample? This would imply a larger-scale influence of the rock record on patterns in the fossil record during the mid Eocene (see more below).

Author response: We were using "sampling bias" in this place to denote biases in the fossil record more generally. We changed the phrasing here accordingly, and took care to discuss these ideas more clearly (see also the two points below).

Lines 305–306: "more extensive record of sedimentary rocks . . ." Here, "extensive" implies greater areal coverage of fossiliferous formations rather than total number of fossiliferous formations, which is what is shown in Fig. S21. I recommend rephrasing this as "increased number of fossiliferous formations" or similar.

Author response: Done, thank you for the clarification.

I think this point deserves more discussion than one parenthetical. It could well be that some formations and localities are screen washed for small mammals more extensively than others, which would increase the richness of glires from those sites. If formations from this interval are underexplored for fossils, then it could be worth calling it to attention to inspire more work on the mid Eocene. If formations from this interval have been explored and very few yield fossils, then that indicates that larger-scale mechanisms could be affecting fossil preservation and the patterns of occurrence.

Author response: We wholeheartedly agree, and unfortunately, we do not have the answer. This would indeed be very important to know moving forward. Often more extensive collection efforts are focused on the periods just before or just after a major boundary too, depending on the research question, which could skew our understanding of true biodiversity patterns at this boundary, or could mean that it both sides of the boundary have been well-sampled.

In any case, we have added some sentences trying to incorporate these ideas here (lines 315-324): "*Our results suggest that the purported extreme increase in Rodentia and Lagomorpha fossil abundance after the EOT can at least partially be attributed to biases in the fossil record, such as an increased number of fossiliferous formations, and subsequently fossils, in the Oligocene compared to the late Eocene [Fig. S17], or possibly more intensive sampling of smaller species via collection techniques such as sieving. Under the common-cause hypothesis mentioned above [56], a lower rock record volume in Asia during the mid-Eocene (which could be suggested by the observed lower number of fossiliferous locations in our dataset; see Table 1) and lower raw fossil diversity (Figs S16 & S17) would both be driven by environmental aridification, which could accentuate increases in the subsequent Oligocene in total diversity and rock volume that are apparent in our datasets. However, this would not immediately explain the differences in richness trajectories in the different clades.*"

Lines 322–323: The middle Eocene shows a drastic decrease in number of fossiliferous formations and associated decrease in generic richness; this coincides with the loss of other mammal clades, which drives the turnover pattern. The authors may include this in the "sampling bias" they mention in line 305 and earlier, but it is worth specifically mentioning the influence of the loss of the record on the data analyses.

Very few fossil localities occur during the interval when it is thought that increased weathering of the Himalaya drew down CO2 and led to mid–late Eocene cooling and cooling at the EOT. Could the coincidence of changes in weathering, climate, and the deposition of fossil-bearing formations point to a common causal mechanism that shapes the rock and fossil records (Peters et al., 2022)? This would not preclude true turnover of clades during this interval, but the loss of the record (from nondeposition or erosion) accentuates the turnover patterns. I think this is an interesting and important part of the data shown here and deserves more discussion.

Author response: Yes, this may well be true, and is accepted by the first author as a plausible hypothesis, if not previously explicitly written in our manuscript because this is very difficult to disentangle.

We have added the following sentences. Lines 289-292: "*Alternatively or additionally, this aridification may have resulted in both the reduction of geological sections (e.g. the Irdinmanhan – Ulangochuian; ~45 – 37 Ma) and the contemporary richness decline and turnover of taxa, indicating environmental aridification as a common cause for rock record volume and fossil diversity [55,56].*"

Lines 319-324: "*Under the common-cause hypothesis mentioned above [56], a lower rock record volume in Asia during the mid-Eocene (which could be suggested by the observed lower number of fossiliferous locations in our dataset; see Table 1) and lower*

raw fossil diversity (Figs S16 & S17) would both be driven by environmental aridification, which could accentuate increases in the subsequent Oligocene in total diversity and rock volume that are apparent in our datasets. However, this would not immediately explain the differences in richness trajectories in the different clades."

In addition, we have added a paragraph to the supplement under the "Supplementary Results & Discussion Raw Diversity Patterns" heading: "*Although bias of the fossil record could explain this pattern, it could also be a real signal that indicates a common cause for rock availability and fossil diversity (Peters et al., 2022). In either case, it is important to statistically account for these apparent correlations when estimating fossil diversity, to ensure comparability of diversity estimates across time intervals.*"

Line 340: Are the geological records mentioned here the weathering intensity proxies shown in Fig. 4? If so, cite the reference here.

Author response: Yes, done. Thank you!

Line 359: Specify total latitudinal range, since no occurrences from Russia were analyzed. So, 30–50° to match Fig. 1a?

Author response: Our analyses include only modern-day Mongolia and China above 30° N. Therefore, the upper latitudinal range is the most northerly Mongolian locality included in our dataset. This differs very slightly from our palaeomap latitudinal range, because this has been projected back to the Eocene. For this reason, it may be better to leave the explanation of our study area as "Mongolia and China above 30° N?

Line 362: Cite the primary literature from which additional occurrences were obtained, or include reference list in supplemental material.

Author response: Thank you for the suggestion. We now include a full bibliography of primary literature (sources where we collected fossil occurrences and measurements relating to body mass for this paper) at the end of the supplement.

Lines 363–365: Mention the number of localities as well as taxon occurrences and genera. It would also be useful to know the number of localities per ALMA, perhaps shown as a table in the supplemental material.

Author response: Thank you for your suggestion. A table has been added to the main text reflecting the number of occurrences, unique taxa and unique localities across the two datasets (Table 1). We have also added the total number of unique localities to the main text as follows (lines 376-381): "*After the dataset was cleaned (see Appendix S1 for additional information on data cleaning and filtering), the resulting total genus-level dataset had 1887 occurrences, 393 unique genera and 426 unique localities, and the*

species-level dataset had 1872 occurrences, 537 unique species and 365 unique localities (Dataframe S1; see Fig. S1 for species level results. See Table 1 for number of fossil occurrences, unique taxa and unique localities per midpoint-based Asian Land Mammal Age across both datasets)."

Lines 369–371: Was the age bootstrapping done for each fossil occurrence or each taxon occurrence? Perhaps specify "taxon" instead of "fossil" in lines 370 and 371, as "fossil" to me means each element recovered from a locality.

Author response: Age bootstrapping was done for each fossil occurrence in a time bin, so I have left this as "fossil" (please see our response to Reviewer #2 above RE how age bootstrapping was implemented).

Line 416: Cite the primary literature from which body-mass data was collected, or include reference list in supplemental material.

Author response: Thank you for the suggestion. We now include a full bibliography of primary literature (sources where we collected fossil occurrences and measurements relating to body mass for this paper) at the end of the supplement.

Line 431: Simulations for these time slices do not seem to be published in the Valdes et al. reference given. I did not thoroughly read this reference, but it appears to be more of a newsletter than a research article. The article in Valdes et al. that relates to Eocene climate does contain links to paleo-climate data sources, but more information is needed about where these published simulations occur. The authors should check this citation or provide more information about where these simulations were published or how the simulations were accessed.

Author response: We can only apologise, there seems to have been an error on our side when we added the Valdes 2021 reference to our manuscript using a reference management software. The wrong Valdes et al. 2021 has been cited here and in other places. We meant to cite "Valdes, P. J., Scotese, C. R. & Lunt, D. J. Deep ocean temperatures through time. Climate of the Past 17, 1483–1506 (2021).". This has now been corrected throughout. We are really grateful that you caught this and are embarrassed that we made the mistake. Thank you for being so thorough in your review.

In addition, we added information on where exactly these models can be accessed. This section now reads (lines 454-460): "*Precipitation and potential evaporation output was extracted from published climate model simulations (available at https://www.paleo.bristol.ac.uk/ummodel/scripts/papers/Valdes_et_al_2021.html) for 56 Ma, 52 Ma, 45 Ma, 30 Ma, 36 Ma, 31 Ma and 26 Ma [41]. The simulations were run with a*

coupled atmosphere-ocean-vegetation model (HadCM3BL-M2.1aD) with a horizontal resolution of 3.75 x 2.5 (longitude x latitude) degrees (~300km x 275km for the mid-latitudes) [41] which is a variant of the Hadley Centre model HadCM3 [70]."

Lines 439–442: Do both CO2 scenarios come from Foster et al.? This reference should be cited earlier as the source of the two CO2 curves. Also, what are the criteria for a "successful" simulation? Did the model closely match climate proxy data, or did it simply produce output? Add additional information to increase clarity.

Author response: The Foster curve is a LOESS fit to CO_2 proxy data. The additional curve (smooth) is developed by Valdes *et al.* 2021 (now cited in the aforementioned paragraph) by using simulations of the last 440 Ma for various CO_2 concentrations and finding the CO_2 concentrations which best match with proxy data. These CO_2 values were linearly interpolated to get CO_2 values for the stages in Valdes *et al.* 2021.

We have added this citation to the first time we discuss the CO_2 curves in the Results (Line 157) and to the Methods, thank you for the suggestion. The sentence mentioning "successful simulations" in the methods was further clarified as well.

Line 629: How were the "representative" Ai maps produced? Does this output represent a combination or average of the two CO2 scenarios? I don't recall seeing any information in the Methods or supplemental material about how the representative models were produced.

Author response: Thank you for noticing this. The maps in Figure 1 represent CO_2 scenario 1, and this information has now been added to the figure legend. We have also removed "representative" for the benefit of "selected", as we meant the selected maps are representative of other time periods.

Line 675: Cite the original source(s) of the CO2 scenarios.

Author response: Done here and in the Figure 1 caption as well.

Figure 4: In b) is the dotted line at Ai=0.2 meant to show the cutoff for desert-like conditions? If so, please add a line in the figure caption to explain.

Author response: Thank you for the suggestion. We have added the following to the Figure 4 figure legend, under b) (Lines 731-732): "*The horizontal dashed grey line represents an A_I of 0.2, with environments below that classified as arid.*"

I know that the formatting and design of information and figures in supplemental information does not require the same degree of scrutiny as the manuscript text and figures; however, I feel that some minor changes would improve clarity for readers who seek out the supplemental files.

We appreciate your time looking through our supplement in depth as well. To improve readability we also now include a table of contents and have made multiple edits to language to improve clarity.

Page 7: The description of geographic area and sampling-standardized diversity ends mid-sentence.

Author response: Thanks so much for catching that! This section now describes the method and results.

Table S2: Consider reducing the significant digits of the percentage of taxa with body-mass data. This will increase legibility, and this degree of precision is not necessary.

Author response: Done. Thank you!

Figure S18: It seems that Fig. S15 is incorrectly referenced here. Is the caption meant to refer to Fig. S17?

Author response: You are correct, implemented.

Figure S21: Consider using dashed lines instead of dotted lines. Dotted lines are unusual in line graphs because dots could be mistaken for data points.

Author response: Changed, thank you.

Peters, S.E., Quinn, D.P., Husson, J.M., and Gaines, R.R., 2022, Insights into cyclic and secular evolution of the Earth-life system: Annual Reviews in Earth and Planetary Science, v. 50, p. 419–449.

Author response: Reference added as a result of adding a short piece to the discussion about potential common cause mechanisms, thank you for the suggestion.

The code files appear to be the raw inputs to R as used by the authors. Many data file names and file locations are retained, and it is not always clear how these files translate to the data files (.xlsx) provided as supplemental info (and some do not seem to be provided). Some light cleaning of the code could improve useability, or a README file could help translate between the appropriate data file and the code implementation.

Author response: Yes, the code that was submitted previously was difficult to follow, we apologise. All code will be cleaned, working directories deleted, and unused datasets and objects removed prior to uploading the code for use. We will also prepare readme files which link the supplementary dataframes to files called in R. We have uploaded an

example of our code cleaning; “Asia Mammal Genus Code Clean.R”.

Specific comments

Lines 45 and 49: Use EM dashes to separate the clauses, not EN dashes.
Done, thanks.

Line 70: Need a comma at the end of the phrase beginning “a key period in mammal evolution . . .”
Done.

Lines 97 and 98: Use EM dashes to set off the clause after “Lagomorpha,” not hyphens.
Done.

Line 679: A typo—Asian LMA, not American.
Done. Thanks so much for catching that! A consequence of having worked with NALMAs for many years previously!

Line 688: Can omit “(Ai)” since it appears at line 682.
Done.

Supplemental material

Figure S14: Consider adding a sentence to the caption explaining the dashed lines in the figure.
The following has been added to the legend of Figure S14.
The horizontal dashed line at 0.2 on the y-axis (A_I) denotes the transition between arid (below) and semi-arid climate conditions (above). The horizontal dashed line at 0.65 on the y-axis (A_I) denotes dryland climate conditions below this line.

Reviewer #3 (Remarks on code availability):

I have reviewed the example revised code, and it is much more user-friendly than the original files. I thank the authors for taking the time to modify their code.
