# Supplementary material for: Global and regional climate change impacted Paleogene mammal faunas in Central Asia
Source: Nat Commun. 2026 Sep 8;17:9606. doi: 10.1038/s41467-026-77374-7 (PMC13554191; doi:10.1038/s41467-026-77374-7)
Supplement: Supplementary file 3 [file 41467_2026_77374_MOESM1_ESM.pdf]

Supplementary Information for Benevento *et al.* **Global and regional climate change impacted Paleogene mammal faunas in Central Asia.**

Supplementary Results & Discussion **pp. 2-7**

Supplementary Note 1: Sensitivity Analyses *pp. 2*

Supplementary Note 2: Body Size Patterns Through Time *pp. 2-3*

Table S1 *pp. 2*

Supplementary Note 3: Geographic Area and Sampling-standardized Taxonomic Richness *pp.3*

Table S2 *pp. 4*

Supplementary Note 4: Raw Diversity Patterns *pp. 4-5*

Supplementary Note 5: Aridity Index (AI) Distributions for Perissodactyla, Rodentia and Glires *pp.5*

Table S3 *pp. 6*

Supplementary Note 6: Sampling-standardized Richness of Species *pp. 6*

Supplementary Note 7: Sampling-standardized Turnover *pp. 7*

Supplementary Figures **pp. 12-30**

Figure S1 *pp. 8*

Figure S2 *pp. 9*

Figure S3 *pp. 10*

Figure S4 *pp. 11-12*

Figure S5 *pp. 13*

Figure S6 *pp. 14*

Figure S7 *pp. 15-16*

Figure S8 *pp. 17-18*

Figure S9 *pp. 19*

Figure S10 *pp. 20*

Figure S11 *pp. 21*

Figure S12 *pp. 22*

Figure S13 *pp. 23-24*

Figure S14 *pp. 25*

Figure S15 *pp. 26*

Figure S16 *pp. 27*

Figure S17 *pp. 28*

Figure S18 *pp. 29*

Figure S19 *pp. 30*

Supplementary References **pp. 31**

Supplementary Results & Discussion

Supplementary Note 1: Sensitivity Analyses

In each of the sensitivity analyses for total Mammalia (Figs S2a-c), the overall pattern of Paleocene/Eocene richness increases, mid-Eocene richness decreases, and Eocene/Oligocene richness decreases persisted. Crucially, the Sharamurunian ALMA (the bin following the proposed onset of Central Asian aridity) showed the lowest reconstructed number of genera of all Eocene bins in all analyses, suggesting that our main finding that mammals suffered diversity losses during intense regional aridification is robust to geographic sampling.

Supplementary Note 2: Body Size Patterns Through Time

Body size data were collected for species across different mammal clades. Percentages of species with data on body size was above 60% in each clade, with the vast majority of clades having > 70% (Table S1).

	Total Species in Cleaned Dataset	Species with Body Size Data	Percentage
**Mammalia**	537	389	72.44
**Rodentia**	162	125	77.16
**Glires**	234	178	76.07
**Lagomorpha**	21	20	95.24
**Other Glires**	51	36	70.59
**Perissodactyla**	84	60	71.43
**Artiodactyla**	28	17	60.71
**Ungulates**	118	79	66.95
**Other Mammalia**	184	133	72.28

Table S1: Table summarizing the total number of species in our dataset for each clade, as well as the number and percentage of these species with body size data.

After sample-standardization, some patterns of body size change through time were generally preserved across different sampling replicates (Figs S4 & S5). Firstly, the Paleocene (particularly the first two time intervals) showed a reduced range of body sizes compared to later time bins. Minimum body size then appears to

have decreased across the Paleocene/Eocene boundary. Body size median and maximum values showed a significant reduction during the middle Eocene (Sharamurian) and increases during the last two intervals of the Eocene. In every sampling replicate, median body size decreased into and throughout the Oligocene.

Individual clades showed variation in their body size patterns through time (Fig. S7). In general, ungulates (including Perissodactyla) showed a pattern of increasing minimum and maximum body size through time (Fig. S7e, f). Lagomorpha (Fig. S7c) showed a significant body size decline across the Eocene/Oligocene boundary. For some clades (Glires, Rodentia and 'other Mammalia' excluding Glires and ungulates; Fig. S7a, b, h), at least median, and sometimes maximum and/or minimum, body size appears to have decreased during the Sharamurunian (mid-Eocene). Only the 'other Mammalia' showed a clear pattern of body size (median, maximum and minimum) decrease after the Eocene/Oligocene boundary (Fig. S7h).

Sampling standardization showed very similar patterns of clade-level body size evolution through time (Fig. S8); therefore, we feel confident that the general patterns of body size evolution for each clade are relatively robust to sampling.

Supplementary Note 3: Geographic Area and Sampling-standardized Taxonomic Richness

In order to test whether sampling-standardized richness estimates were controlled by the amount of area covered by fossil localities in each time bin, we ran correlation tests between the minimum spanning tree distance between all localities in a given time interval and the sampling-corrected richness estimate for this bin (Figs S11 & S12). We found no statistically significant correlation between geographic minimum spanning tree distance and sampling-corrected genus richness (Table S2).

	cor	p-value	t	df	Upper 95% Confidence Interval	Lower 95% Confidence Interval
**Total Mammalia**	0.075	0.836	0.214	8	0.673	-0.582
**Rodentia**	-0.274	0.511	-0.699	6	0.533	-0.820
**Glires**	0.039	0.910	0.116	9	0.624	-0.575
**Perissodactyla**	-0.038	0.936	-0.084	5	0.736	-0.769
**Artiodactyla**	-0.334	0.783	-0.355	1	NA	NA
**Ungulates**	0.052	0.912	0.116	5	0.775	-0.730
**Other Mammalia**	0.296	0.439	0.820	7	0.802	-0.458
**Total Mammals and All Clades Combined**	0.198	0.148	1.467	53	0.440	-0.071

Table S2: Two-sided Pearsons correlation test results for geographic area and sampling-standardized richness estimates for mammalian genera, repeated across all clade datasets.

Supplementary Note 4: Raw Diversity Patterns

It is now well-established that patterns from the raw fossil record can be severely affected by different biases, including geological bias and researcher sampling bias. Many of the patterns proposed in earlier works, such as a turnover from Perissodactyla to Rodentia and Lagomorpha [1], stem from the raw record. At the time of some of these early papers on the subject, many of the sampling correction methods were either not developed, or in their infancy. Meng and McKenna [1] wrote '*Taphonomy, palaeoecology, and methods of collecting are common factors that may bias fossil assemblage data … but these factors are unlikely to result in such consistent proportions of faunal composition and size structure for the faunas that came from different sediments of the same or different ages*'; pp. 365. We disagree that sampling could not produce a consistent temporal pattern, because rock availability and researcher efforts can be biased toward one time interval compared to others. In fact, our data show a correlation between many of the peaks and troughs seen in the number of geological formations per time bin with raw genus diversity (Fig. S15a) and raw fossil abundance (Fig. S15b) through time. In particular, this suggests that the early-mid Oligocene of Central Asia had higher rock

availability as well as more fossil occurrences, which could explain the apparent increase in diversity across the Eocene/Oligocene boundary seen in the raw fossil record (Fig. S14). Although bias of the fossil record could explain this pattern, it could also be a real signal that indicates a common cause for rock availability and fossil diversity [2]. In either case, it is important to statistically account for these apparent correlations when estimating fossil diversity, to ensure comparability of diversity estimates across time intervals.

Supplementary Note 5: Aridity Index (AI) Distributions for Perissodactyla, Rodentia and Glires

To assess whether Perissodactyla and Rodentia (or Glires) may have occupied different climate niches during the Paleocene, Eocene and Oligocene in our study region, we compared the aridity index range for the three clades, respectively (Figs S18 & S19. We then performed Welch's two sample t-tests between results for Perissodactyla and each of the other two clades (Table S3).

	t	p-value	df	Upper 95% Confidence Interval	Lower 95% Confidence Interval
**Perissodactyla and Rodentia Foster Curve**	-5.241	5.382E-07	148.807	-0.020	-0.044
**Perissodactyla and Rodentia Smooth Curve**	-5.778	3.810E-08	160.721	-0.029	-0.058
**Perissodactyla and Glires Foster Curve**	-5.442	2.176E-07	145.671	-0.021	-0.045
**Perissodactyla and Glires Smooth Curve**	-5.912	2.058E-08	155.950	-0.029	-0.059

Table S3: T-test results for the reconstructed distributions of aridity index (AI) across localities containing Perissodactyla vs. Rodentia (rows 1-2) and Perissodactyla vs. Glires (rows 3-4), using both the ‘CO_2 Scenario 1’ ([3]; rows 1 and 3) and ‘CO_2 Scenario 2’ ([4]; rows 2 and 4) curves.

Supplementary Note 6: Sampling-standardized Richness of Species

Species-level richness curves (Fig. S1) appeared broadly similar to the genus-level curves. There was also an approximately three-fold increase in richness across the Paleocene/Eocene boundary at the species level. Species richness is lowest in the mid- to late-Eocene (Sharamurunian and Ulangochuian). Species-level patterns may differ slightly from genera in that species richness stayed low or perhaps even declined into the Ulangochuian. Species richness decreased across the Eocene/Oligocene boundary and again across the Hsandagolian/Tabenbulakian boundary, mirroring the patterns recovered for richness among genera.

Supplementary Note 7: Sampling-standardized Turnover

For both RAC distance metrics used (modified Forbes, Fig. S3a and Sorenson, Fig. S3b), estimated turnover in mammalian genera between ALMA bins showed a pattern of highest turnover throughout the Paleocene, across the Paleocene/Eocene boundary, during the middle Eocene and across the Eocene/Oligocene boundary. The early and late Eocene showed markedly lower turnover. Larger bins (Paleocene/Eocene, early Eocene/late Eocene and late Eocene/Oligocene) showed relatively high turnover between all tested bins (Fig. S3c) using the Sorenson distance metric.

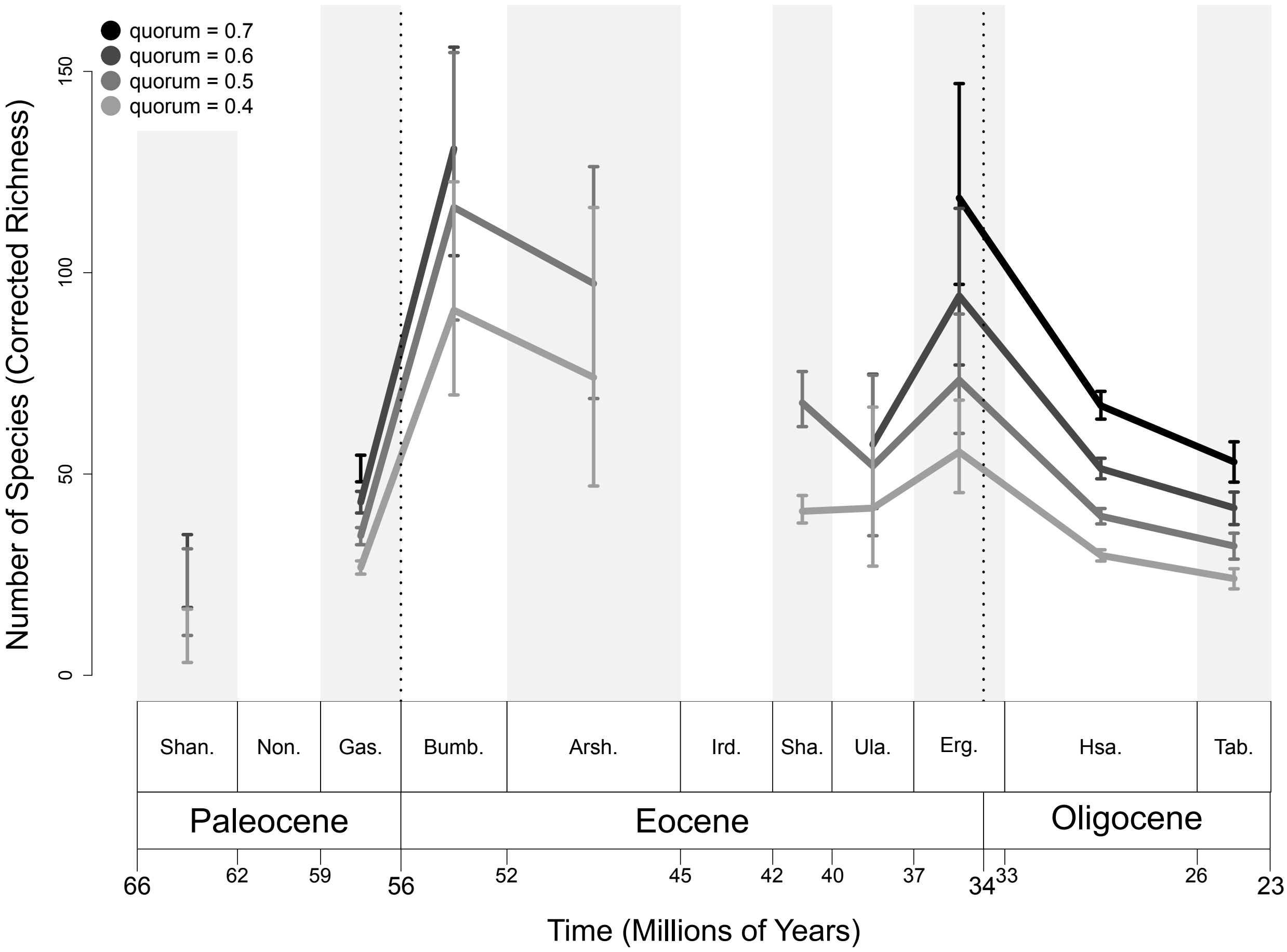

Figure S1. Sampling-corrected number of speices through time for total Mammalia for each Paleogene Aisan Land Mammal Age (ALMA) at quorums 0.4 - 0.7 (coverage-based rarefaction and extrapolation). Mean values are plotted as the centre point derived from 500 technical bootstrap replicates. Error bars show 95% (percentile) confidence intervals also derived from 500 technical bootstrap replicates to assign fossils that span more than one ALMA to a randomized single ALMA within their age range. The absence of sampling-standardised richness results presented for the Irdinmanhan (Ird.) time interval (and for higher quorum values in a few other intervals) reflects a lack of reliably sampled data. ALMA abbreviations are as follows: Shan. = Shanghuan; Non. = Nongshanian; Gas. = Gashatan; Bumb. = Bumbanian; Arsh. = Arshantan; Ird. = Irdinmanhan; Sha. = Sharamurunian; Ula. = Ulangochuian; Erg. = Ergilian; Hsa. = Hsandagolian; Tab. = Tabenbulakian. ALMA time interval abbreviations are the same across all figures and supplementary figures.

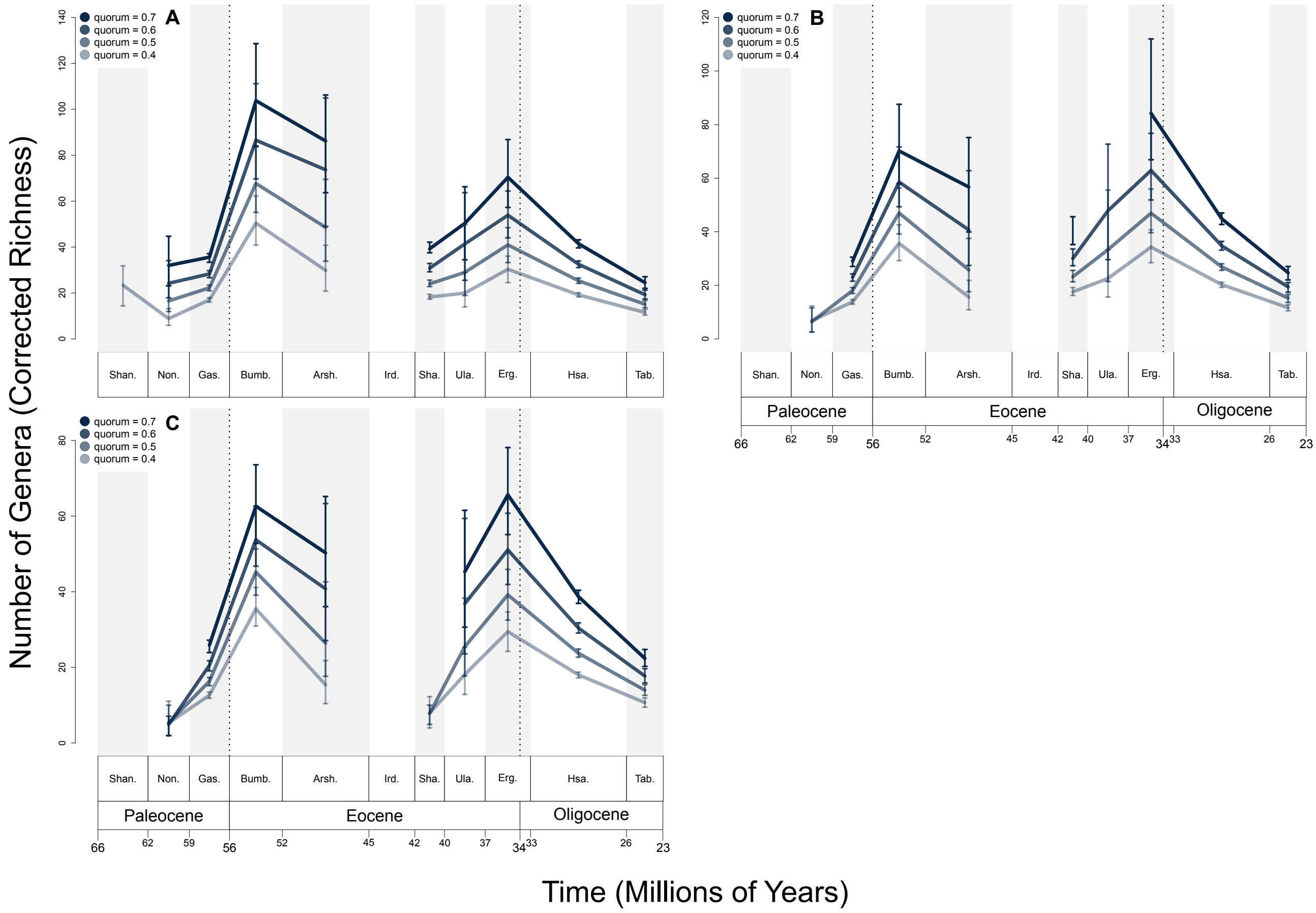

Figure S2. Sensitivity analyses for sampling-corrected richness in mammal genera for each ALMA at quorums 0.4 - 0.7 (using coverage-based rarefaction and extrapolation). a) Sampling-corrected richness in mammal genera using a dataset version with all occurrences with age uncertainties spanning more than two ALMAs removed. b) Sampling-corrected richness in mammal genera using a dataset with a more restricted geographical range, with occurrences to the East of 115° E removed. This was done because some previous work suggests that the region East of 115° E may not have been as dry as the rest of this geographic region. c) Sampling-corrected richness in mammal genera focusing on a geographic range which is more comparable to the geographic range of the data in Meng & McKenna [1], which focused only on Mongolia and Inner Mongolia. All occurrences above 125° E or below 37° N were removed. Mean values are plotted as the centre point derived from 500 technical bootstrap replicates. Error bars show 95% (percentile) confidence intervals also derived from 500 technical bootstrap replicates to assign fossils that span more than one ALMA to a randomized single ALMA within their age range. The absence of sampling-standardised richness results presented for the Irdinmanhan (Ird.) time interval (and for higher quorum values in a few other intervals) reflects a lack of reliably sampled data. . ALMA time interval abbreviations are explained in Fig. S1.

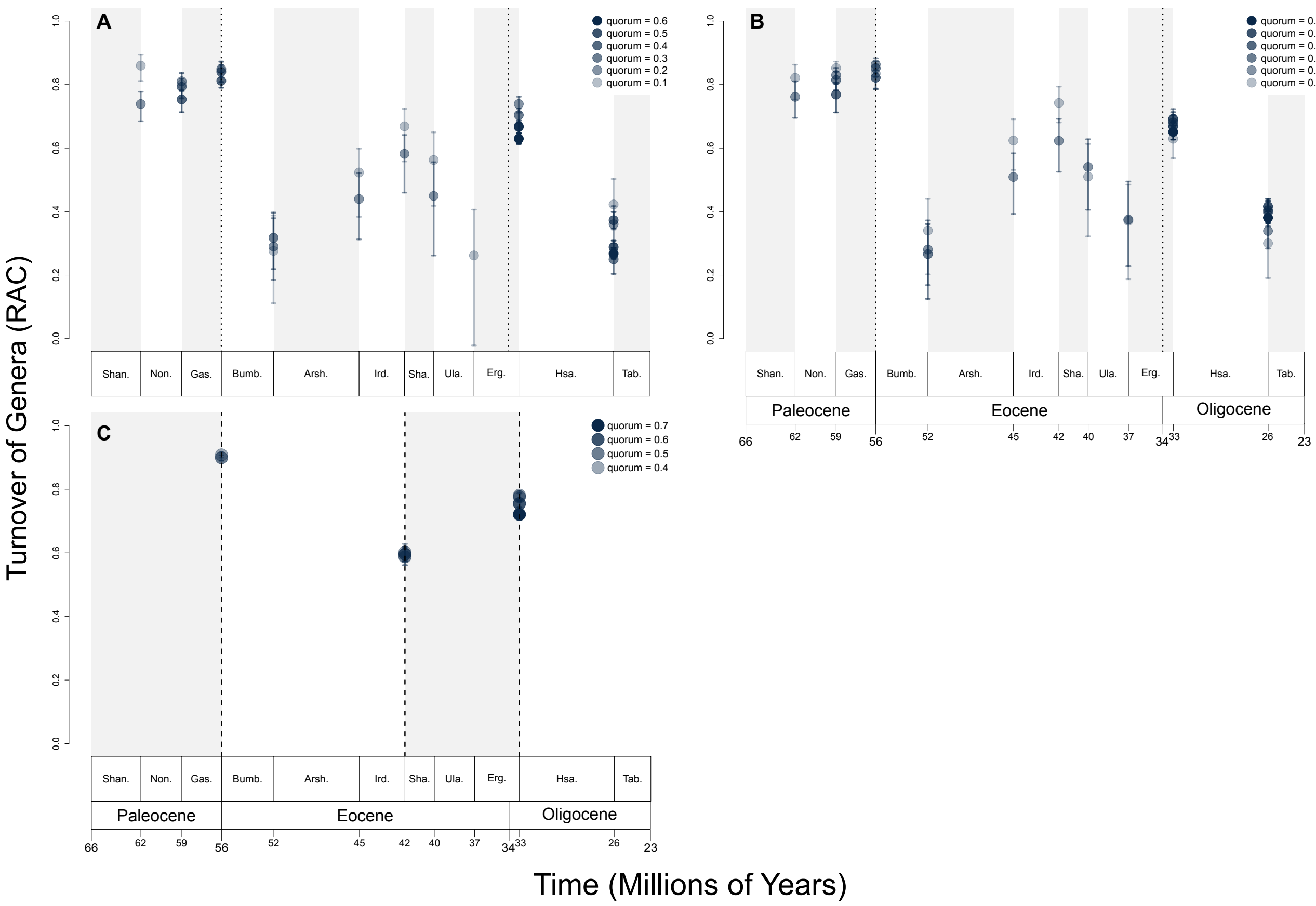

Figure S3. Sensitivity analyses for sampling-corrected turnover of genera for total Mammalia (relative abundance corrected, or RAC). a) Sampling-corrected turnover of genera for total Mammalia for each Paleogene ALMAs, calculated using the modified Forbes distance matrix. This method returns an RAC turnover value of 0 (no turnover) if one dataset is a direct subset of another. b) Sampling-corrected turnover of genera for total Mammalia for each Paleogene ALMAs, calculated using the Sorenson distance matrix. c) Sampling-corrected turnover of genera for total Mammalia for the Paleocene, early Eocene, late Eocene and Oligocene, calculated using the Sorenson distance matrix. Panels a and b use quorums 0.1 - 0.6, and panel c uses quorums 0.4 - 0.7. We acknowledge that quorums 0.1 - 0.3 are extremely low and therefore not particularly reliable, but some bins are not able to be calculated at higher quorums. We include them because, when higher quorums are also able to be calculated for given time bins, the results at these very low quorums appear to be in general accordance with the higher quorums. Centre points represent a mean and error bars represent 5% and 95% percentile (quantile) confidence intervals calculated from 100 replicate data simulations. ALMA time interval abbreviations are explained in Fig. S1.

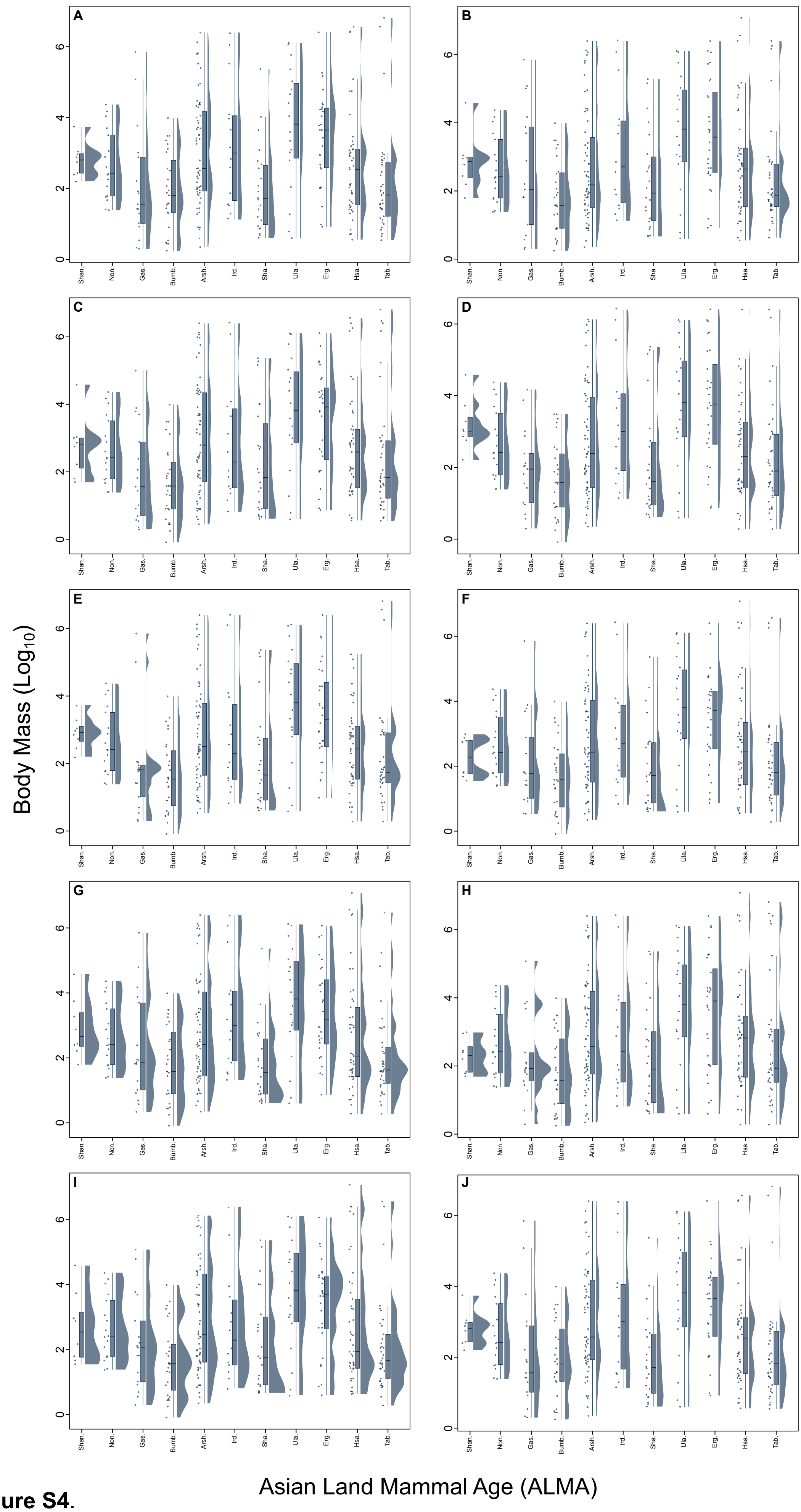

A
B
C
D
E
F
G
H
I
J
Body Mass (Log10)
Shan.
Non.
Gas.
Bumb.
Arsh.
Ird
Sha.
Ula.
Erg
Hsa.
Tab.
Asian Land Mammal Age (ALMA)

Figure S4.

Figure S4. Sampling-corrected species-level body mass (log_{10}-transformed) for total Mammalia, plotted across ALMA time bins. Each time bin is rarefied to different levels based on time bin sample coverage (see supplementary methods). This rarefication process was repeated 1000 times, and each panel represents a different replicate (10 shown in total). a) Rarefied replicate 100, b) rarefied replicate 200, c) rarefied replicate 300, d) rarefied replicate 400, e) rarefied replicate 500, f) rarefied replicate 600, g) rarefied replicate 700, h) rarefied replicate 800, i) rarefied replicate 900 and j) rarefied replicate 1000. Each raincloud plot shows individual species data points (left), box and whisker plots with a centre showing the median, the bounds of the box showing the interquartile range and the whiskers showing the interval containing 90% of the data (middle), and a bisected violin plot illustrating the approximated frequency distribution of individual values (right). ALMA time interval abbreviations are explained in Fig. S1.

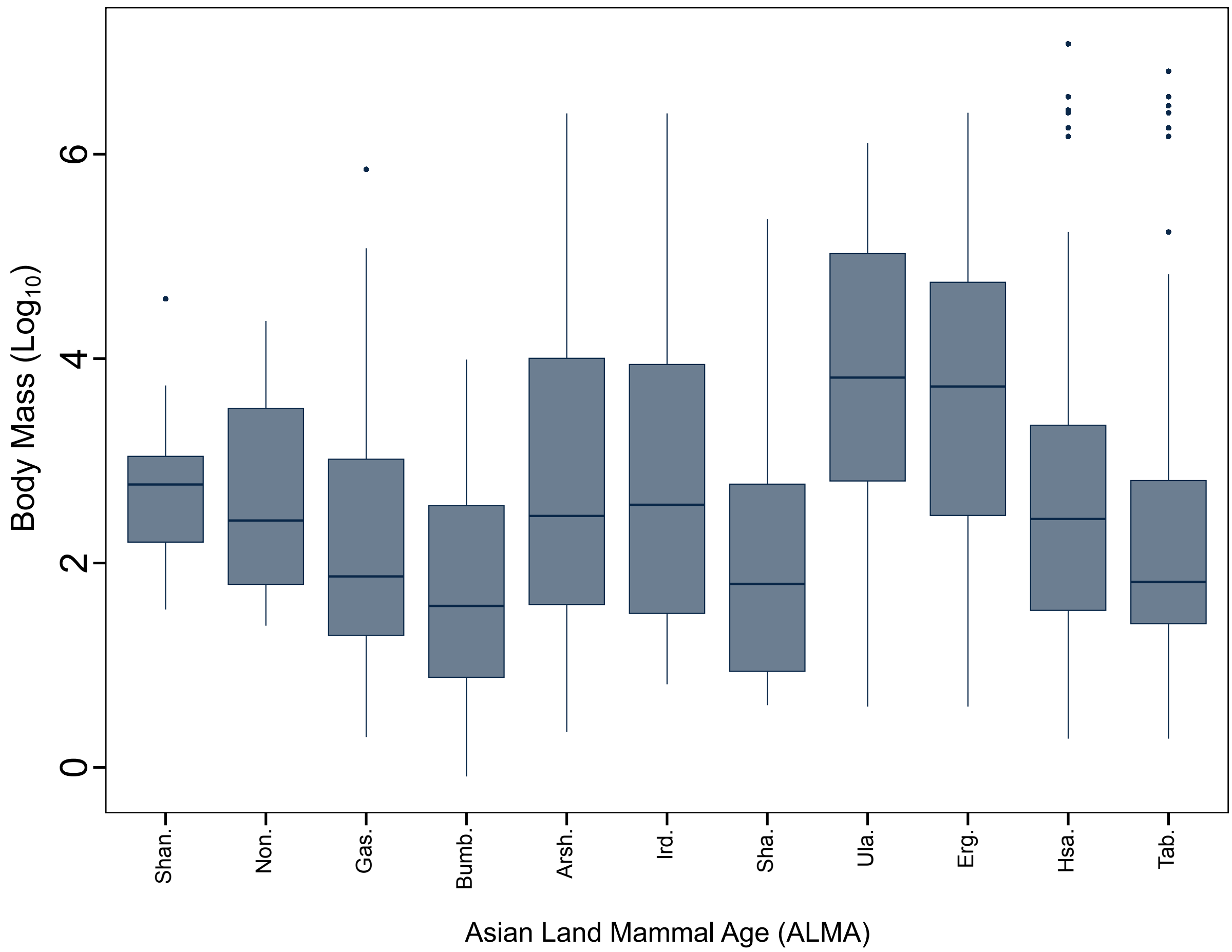

Figure S5. Sampling-corrected species-level body mass ($\log_{10}$-transformed) for total Mammalia, plotted across Asian Land Mammal Age time intervals (ALMAs). Each time interval is rarefied to different levels based on time interval sample coverage. This rarefaction process was repeated 1000 times and an average was taken across all 1000 technical replicates. Box and whisker plots with a centre showing the median, the bounds of the box showing the interquartile range and the whiskers showing the interval containing 90% of the data. ALMA time interval abbreviations are explained in Fig. S1.

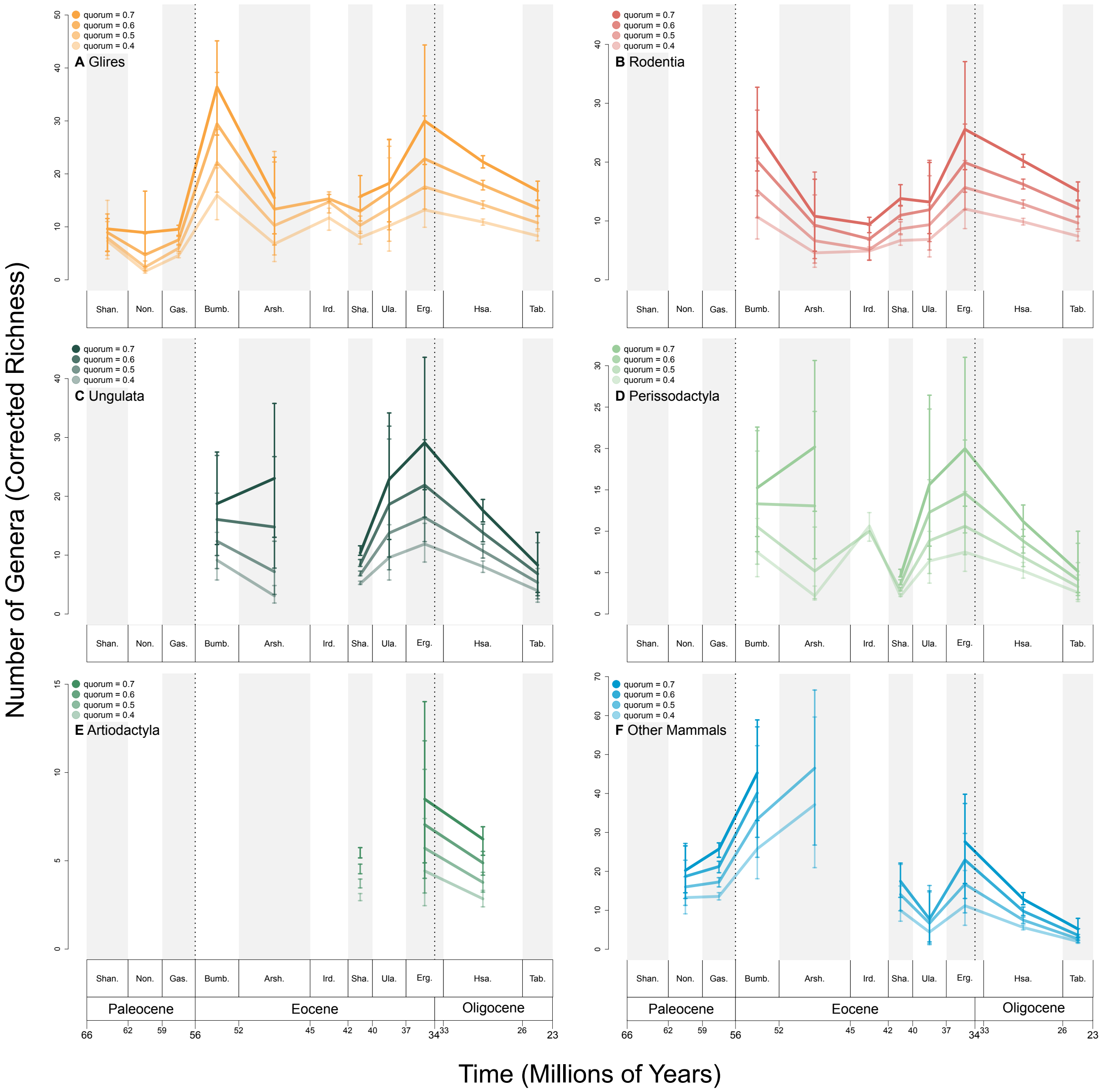

Figure S6. Sampling-corrected number of genera for different mammal clades, across Paleogene Asian Land Mammal Ages (ALMAs), at quorums 0.4 - 0.7 (coverage-based rarefaction and extrapolation). Analyzed mammal clades include a) Glires, b) Rodentia, c) Ungulata, d) Perissodactyla, e) Artiodactyla and f) 'other mammals' (all other mammal clades excluding all Glires and all Ungulata). Mean values are plotted as the centre point derived from 500 technical bootstrap replicates. Error bars show 95% (percentile) confidence intervals also derived from 500 technical bootstrap replicates to assign fossils that span more than one ALMA to a randomized single ALMA within their age range. ALMA time interval abbreviations are explained in Fig. S1.

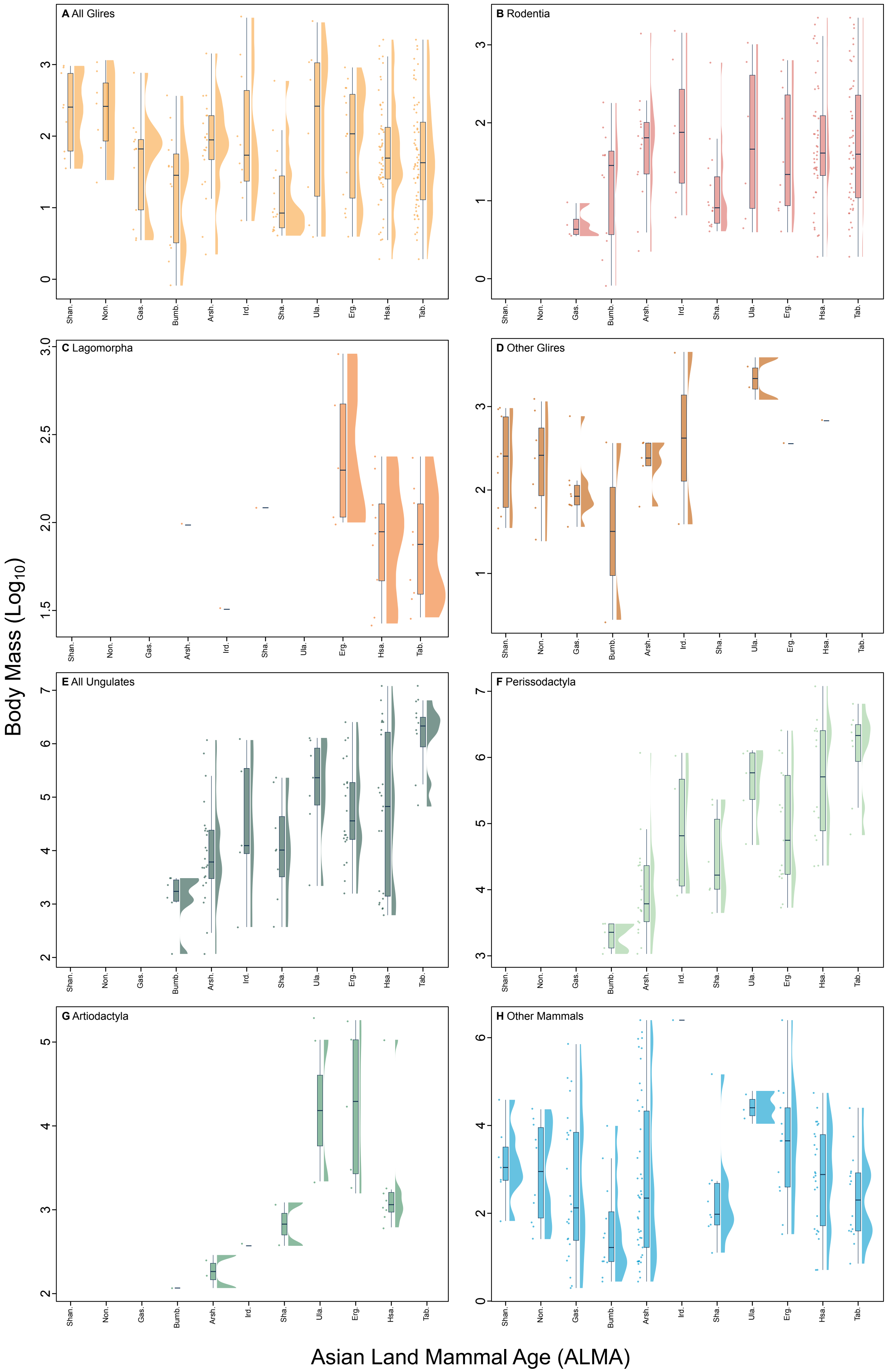
A All Glires
B Rodentia
C Lagomorpha
D Other Glires
E All Ungulates
F Perissodactyla
G Artiodactyla
H Other Mammals
Body Mass (Log10)
Shan.
Non.
Gas.
Bumb.
Arsh.
Ird.
Sha.
Ula.
Erg.
Hsa.
Tab.
Asian Land Mammal Age (ALMA)

Figure S7.

Figure S7. Species-level body mass (log_{10} -transformed) for different mammal clades, plotted across ALMA time bins. Analyzed mammal clades include a) all Glires, b) Rodentia, c) Lagomorpha, d) other Glires, e) all Ungulata, f) Perissodactyla, g) Artiodactyla, h) other Mammalia (all other mammal clades excluding all Glires and all Ungulata). Each raincloud plot shows individual species data points (left), box and whisker plots with a centre showing the median, the bounds of the box showing the interquartile range and the whiskers showing the interval containing 90% of the data (middle), and a bisected violin plot illustrating the approximated frequency distribution of individual values (right). ALMA time interval abbreviations are explained in Fig. S1.

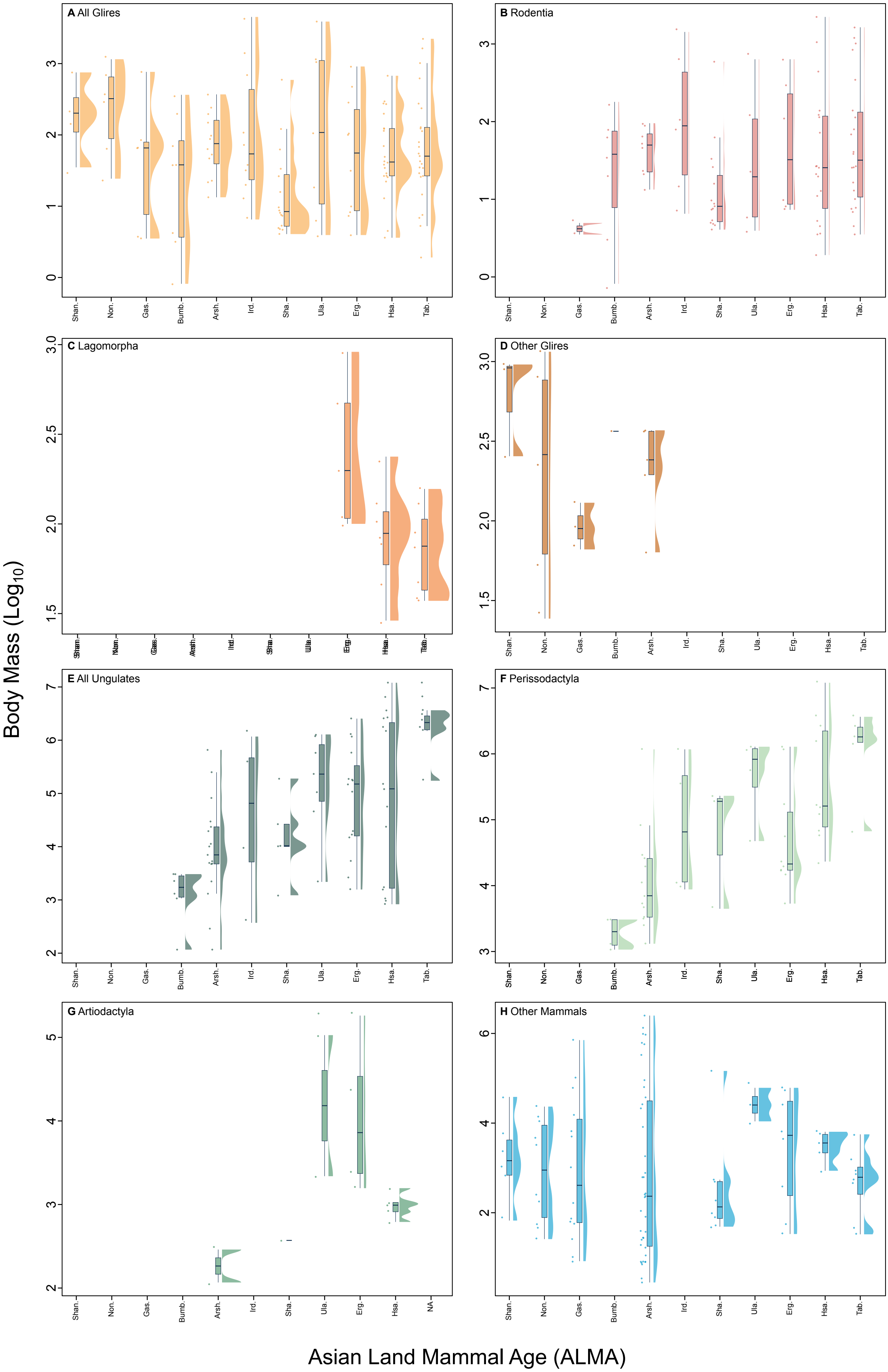

A All Glires
B Rodentia
C Lagomorpha
D Other Glires
E All Ungulates
F Perissodactyla
G Artiodactyla
H Other Mammals
Body Mass (Log10)
Shan.
Non.
Gas.
Bumb.
Arsh.
Ird.
Sha.
Ula.
Erg.
Hsa.
Tab.
NA
Asian Land Mammal Age (ALMA)

Figure S8.

Figure S8. Rarefied Sampling-corrected species-level body mass (log_{10} - transformed) for different mammal clades, plotted across ALMA time bins. Analyzed mammal clades include a) all Glires, b) Rodentia, c) Lagomorpha, d) other Glires, e) all Ungulata, f) Perissodactyla, g) Artiodactyla, h) other Mammalia (all other mammal clades excluding all Glires and all Ungulata). Each time bin is rarefied to different levels based on time bin sample coverage. This process was repeated 1000 times, and here we show run 500 as an example. Each raincloud plot shows individual species data points (left), box and whisker plots with a centre showing the median, the bounds of the box showing the interquartile range and the whiskers showing the interval containing 90% of the data (middle), and a bisected violin plot illustrating the approximated frequency distribution of individual values (right). ALMA time interval abbreviations are explained in Fig. S1.

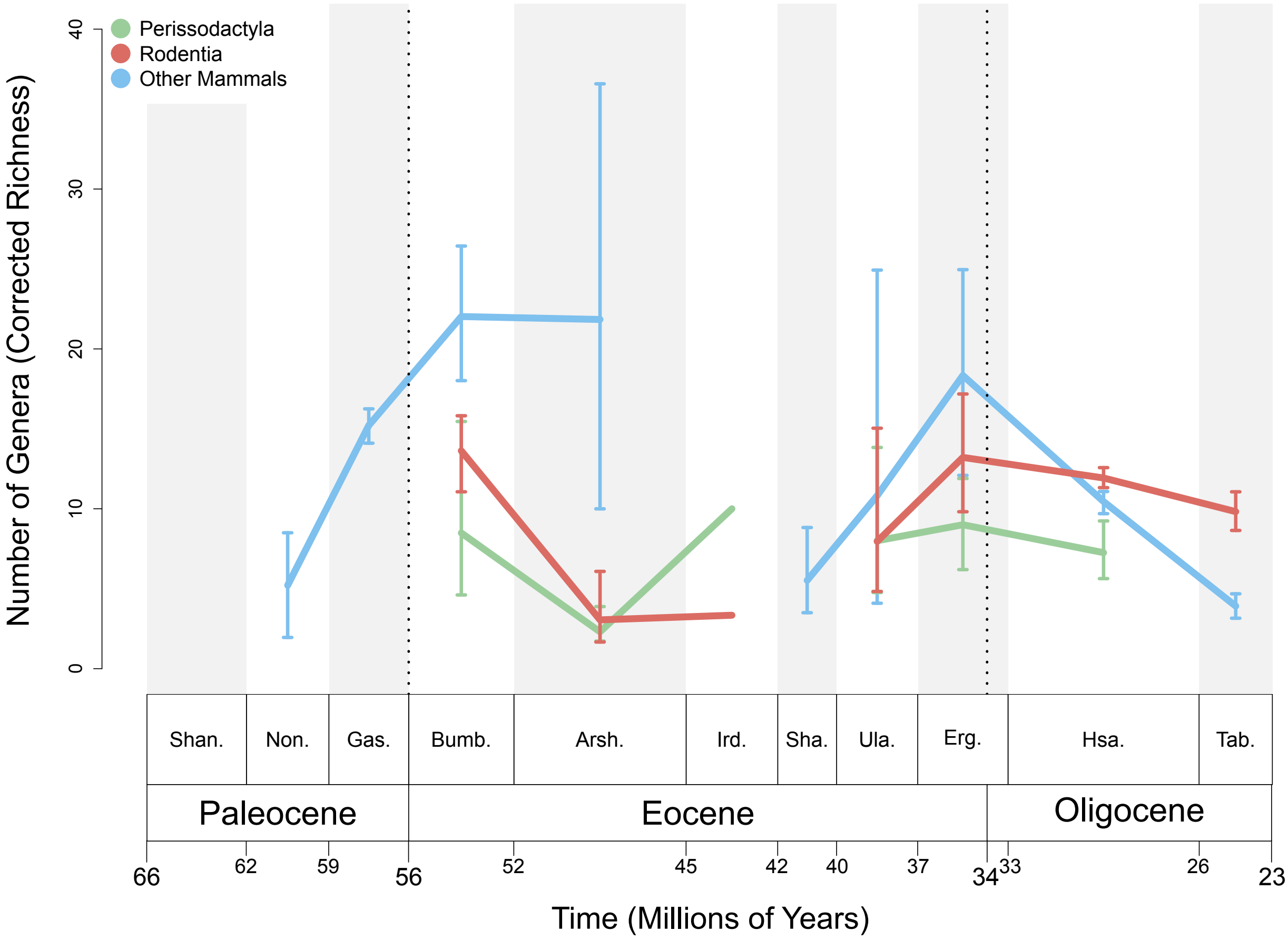

Figure S9. Sensitivity analysis for sampling-corrected number of genera for Rodentia (red), Perissodactyla (green) and other Mammalia (all other mammal clades excluding Rodentia and Perissodactyla (blue) at quorum 0.5 (coverage-based rarefaction and extrapolation) for Paleogene ALMAs (indicated by background shading). A subset of the the main dataset with a more restricted geographical range (which is more comparable to the geographic range of the data in Meng & McKenna [1], which focuses only on Mongolia and Inner Mongolia) was used. All occurrences East of 125° E and or South of 37° N were removed. Mean values are plotted as the centre point derived from 500 technical bootstrap replicates. Error bars show 95% (percentile) confidence intervals also derived from 500 technical bootstrap replicates to assign fossils that span more than one ALMA to a randomized single ALMA within their age range. ALMA time interval abbreviations are explained in Fig. S1.

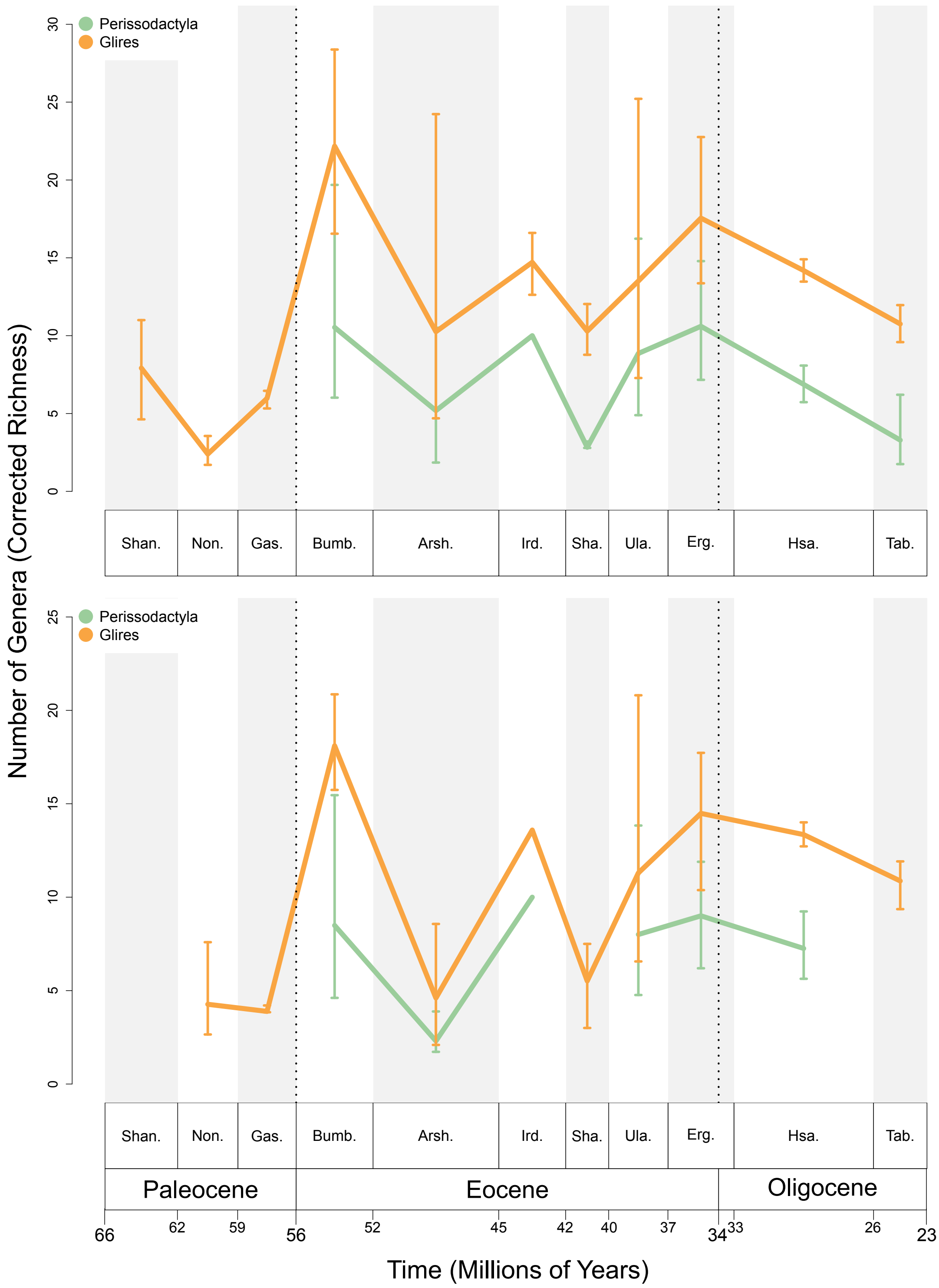

Figure S10. Sampling-corrected number of genera for total Glires (orange), and Perissodactyla (green) at quorum 0.5 (coverage-based rarefaction and extrapolation) for Paleogene ALMAs (indicated by background shading). a) Sampling-corrected number of genera calculated using the entire dataset. b) Sensitivity analysis for sampling corrected number of genera. A subset of the the main dataset with a more restricted geographical range (which is more comparable to the geographic range of the data in Meng & McKenna [1], which focuses only on Mongolia and Inner Mongolia) was used. All occurrences East of 125° E and or South of 37° N were removed. Mean values are plotted as the centre point derived from 500 technical bootstrap replicates. Error bars show 95% (percentile) confidence intervals also derived from 500 technical bootstrap replicates to assign fossils that span more than one ALMA to a randomized single ALMA within their age range. ALMA time interval abbreviations are explained in Fig. S1.

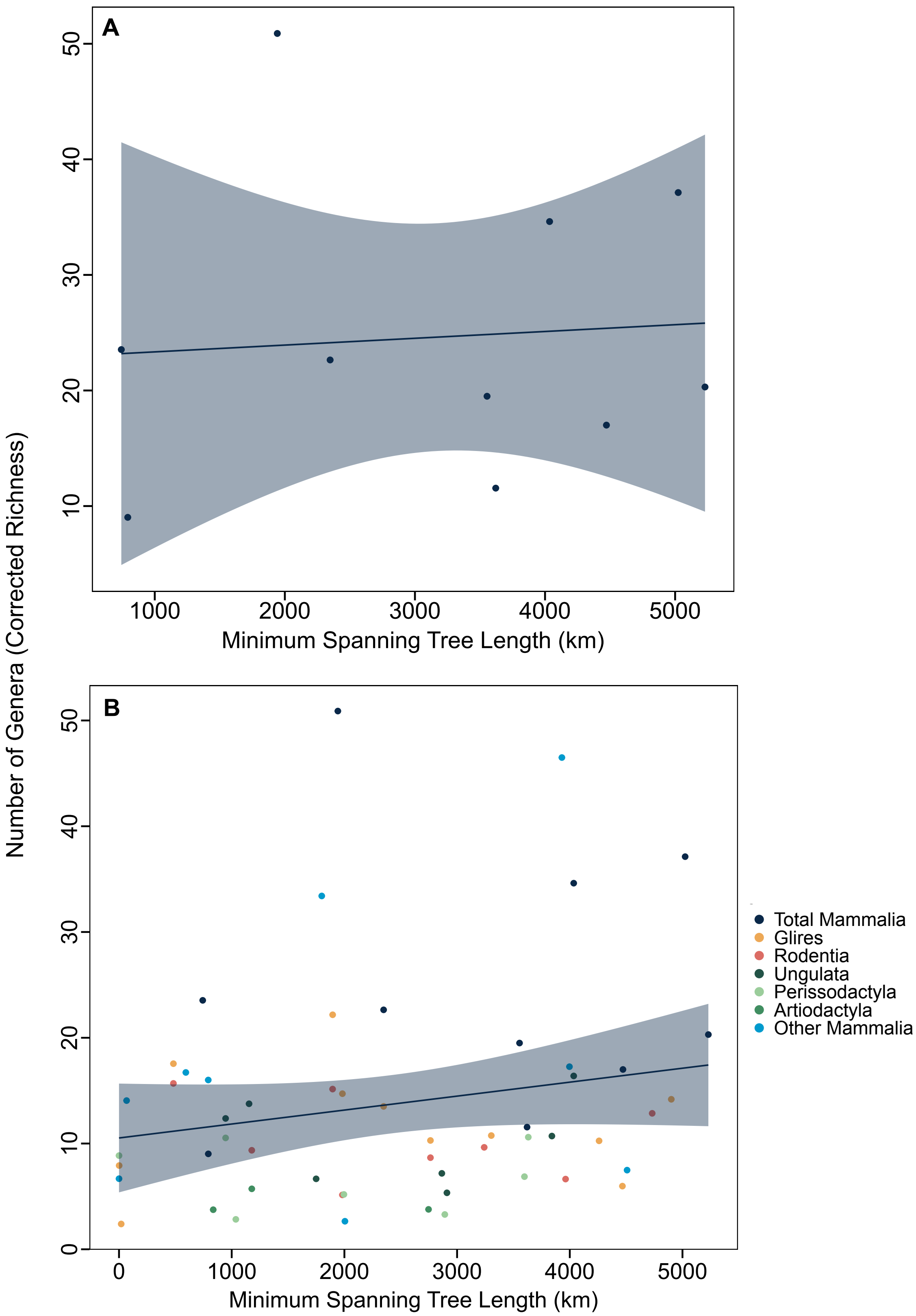

Figure S11. Sampling-corrected number of genera plotted against minimum spanning tree (MST) length of included localities. Points represent individual time intervals. a) Total Mammalia, with a linear best fit line fitted. b) Results for total Mammalia and all subclades included in this study plotted together, with a linear best fit line fitted to all points. Shading around linear best fit line represents 95% confidence interval.

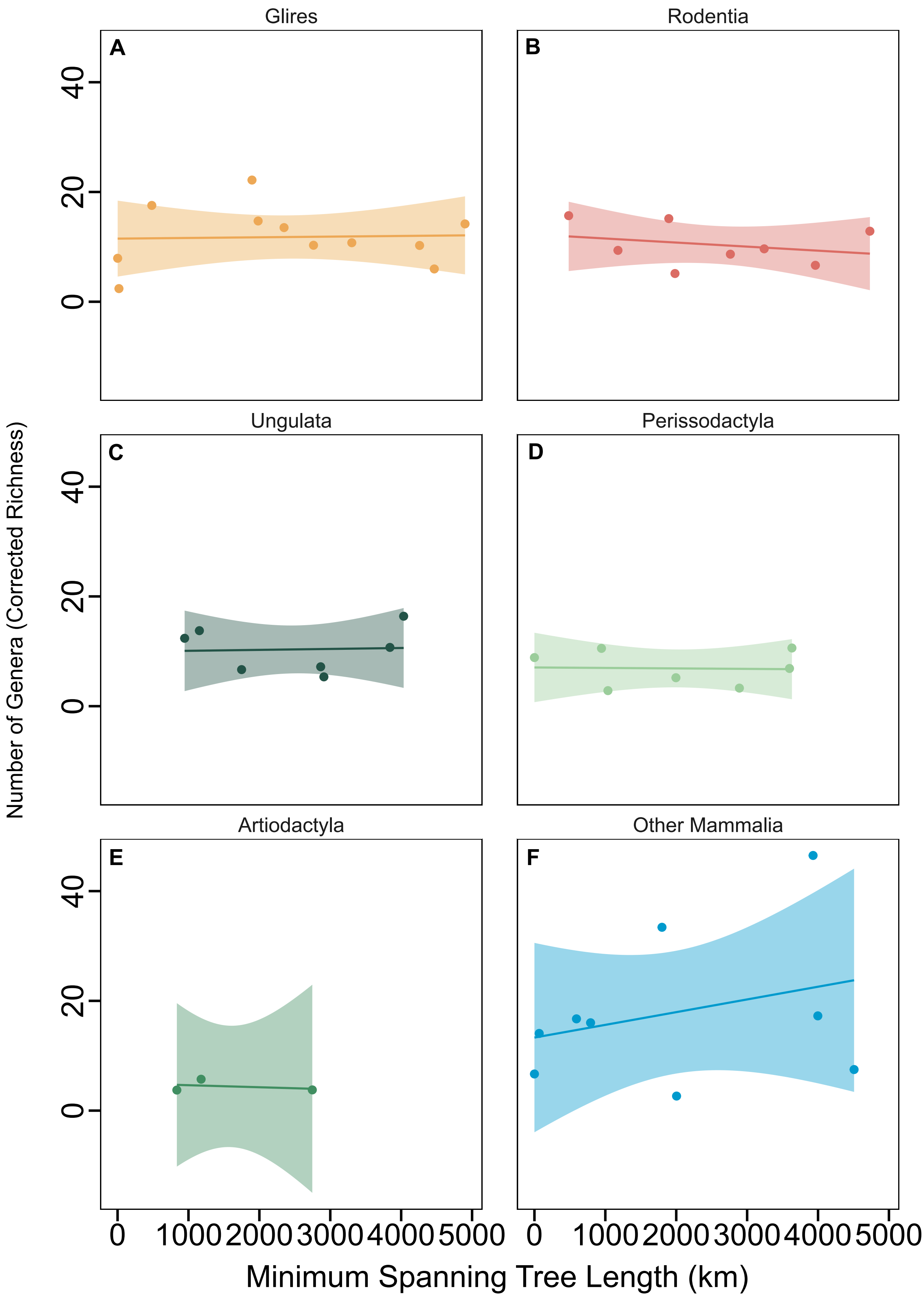

Figure S12. Sampling-corrected number of genera plotted against minimum spanning tree (MST) length of included localities for all subclades. Points represent individual time intervals. a) Glires, with a linear best fit line fitted, b) Rodentia, with a linear best fit line fitted, c) Ungulata, with a linear best fit line fitted, d) Perissodactyla, with a linear best fit line fitted, e) Artiodactyla, with a linear best fit line fitted, and f) other Mammalia (all other mammal clades excluding total Glires and total Ungulata), with a linear best fit line fitted. Shading around linear best fit line represents 95% confidence interval.

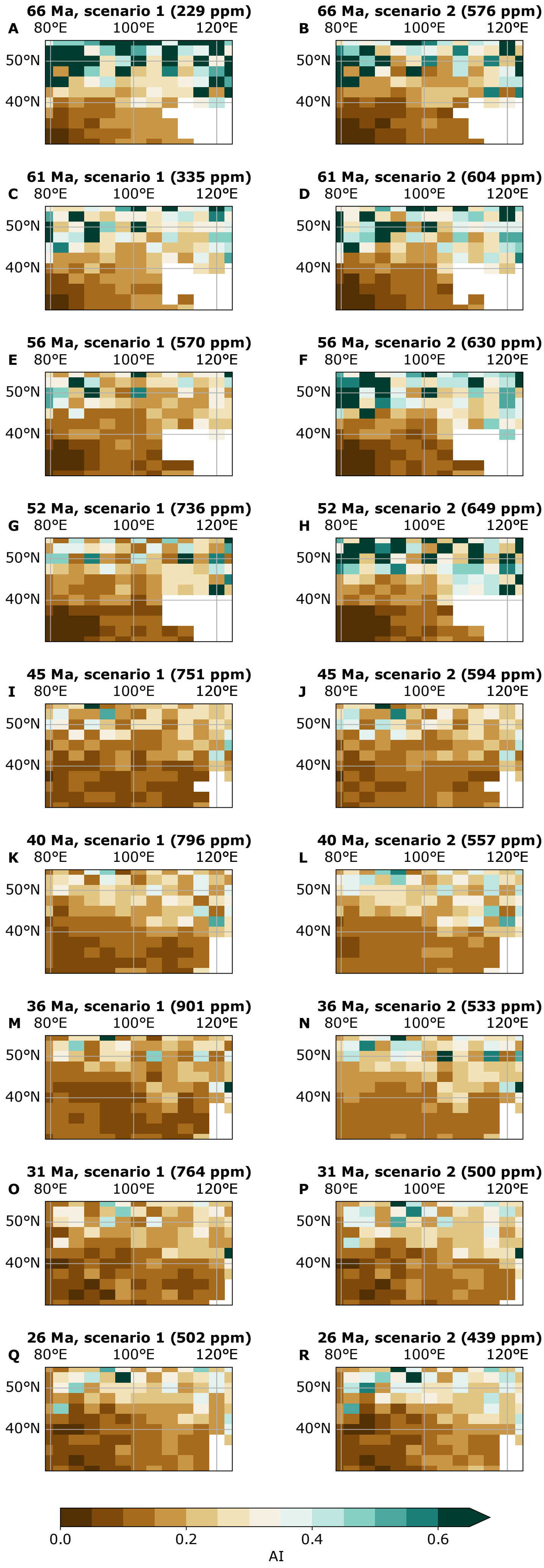
66 Ma, scenario 1 (229 ppm)
A
66 Ma, scenario 2 (576 ppm)
B
61 Ma, scenario 1 (335 ppm)
C
61 Ma, scenario 2 (604 ppm)
D
56 Ma, scenario 1 (570 ppm)
E
56 Ma, scenario 2 (630 ppm)
F
52 Ma, scenario 1 (736 ppm)
G
52 Ma, scenario 2 (649 ppm)
H
45 Ma, scenario 1 (751 ppm)
I
45 Ma, scenario 2 (594 ppm)
J
40 Ma, scenario 1 (796 ppm)
K
40 Ma, scenario 2 (557 ppm)
L
36 Ma, scenario 1 (901 ppm)
M
36 Ma, scenario 2 (533 ppm)
N
31 Ma, scenario 1 (764 ppm)
O
31 Ma, scenario 2 (500 ppm)
P
26 Ma, scenario 1 (502 ppm)
Q
26 Ma, scenario 2 (439 ppm)
R
80°E
100°E
120°E
50°N
40°N
0.0
0.2
0.4
0.6
AI

Figure S13.

Figure S13. Aridity index (AI = prec/E_p) depicted in a Central Asian box (30° N - 55° N and 80° E - 120° E) for two CO_2 scenarios and across nine time slices (66 Ma, 61 Ma, 56 Ma, 52 Ma, 45 Ma, 40 Ma, 36 Ma, 31 Ma and 26 Ma). For each CO_2 scenario, the northern section of this geographical range is wetter (higher AI value) than the southern section, however the transition to an arid climate in the early (‘CO_2 Scenario 1’) or middle Eocene (‘CO_2 Scenario 2’) is nonetheless visible in most grid cells. a) Aridity index (AI) for ‘CO_2 Scenario 1’ at 66 Ma, b) Aridity index (AI) for ‘CO_2 Scenario 2’ at 66 Ma, c) Aridity index (AI) for ‘CO_2 Scenario 1’ at 61 Ma, d) Aridity index (AI) for ‘CO_2 Scenario 2’ at 61 Ma, e) Aridity index (AI) for ‘CO_2 Scenario 1’ at 56 Ma, f) Aridity index (AI) for ‘CO_2 Scenario 2’ at 56 Ma, g) Aridity index (AI) for ‘CO_2 Scenario 1’ at 52 Ma, h) Aridity index (AI) for ‘CO_2 Scenario 2’ at 52 Ma, i) Aridity index (AI) for ‘CO_2 Scenario 1’ at 45 Ma, j) Aridity index (AI) for ‘CO_2 Scenario 2’ at 45 Ma, k) Aridity index (AI) for ‘CO_2 Scenario 1’ at 40 Ma, l) Aridity index (AI) for ‘CO_2 Scenario 2’ at 40 Ma, m) Aridity index (AI) for ‘CO_2 Scenario 1’ at 36 Ma, n) Aridity index (AI) for ‘CO_2 Scenario 2’ at 36 Ma, o) Aridity index (AI) for ‘CO_2 Scenario 1’ at 31 Ma, p) Aridity index (AI) for ‘CO_2 Scenario 2’ at 31 Ma, q) Aridity index (AI) for ‘CO_2 Scenario 1’ at 26 Ma, r) Aridity index (AI) for ‘CO_2 Scenario 2’ at 26 Ma.

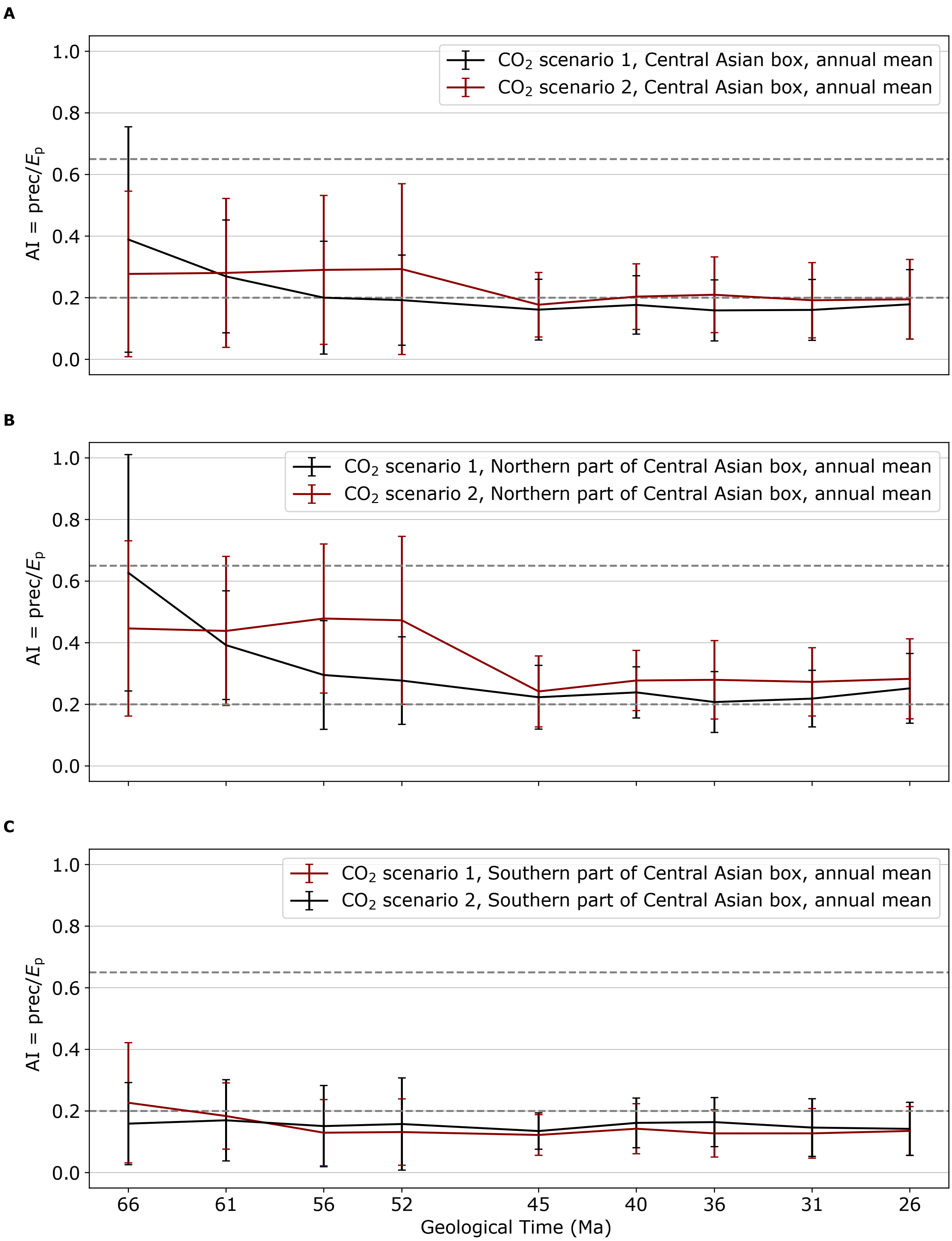

Figure S14. Aridity index AI = prec/E_p for both CO_2 scenarios through time, averaged over a) the entire Central Asian box (30° N - 55° N and 80° E - 120° E, as shown in Fig. S13), b) the northern part of the box only (45° N - 55° N and 80° E - 120° E), and c) the southern part of the box only (30° N - 45° N and 80° E - 120° E). The error bars show the standard error over the respective box regions. The horizontal dashed line at 0.2 on the y-axis (AI) denotes the transition between arid (below) and semi-arid climate conditions (above). The horizontal dashed line at 0.65 on the y-axis (AI) denotes dryland climate conditions below this line.

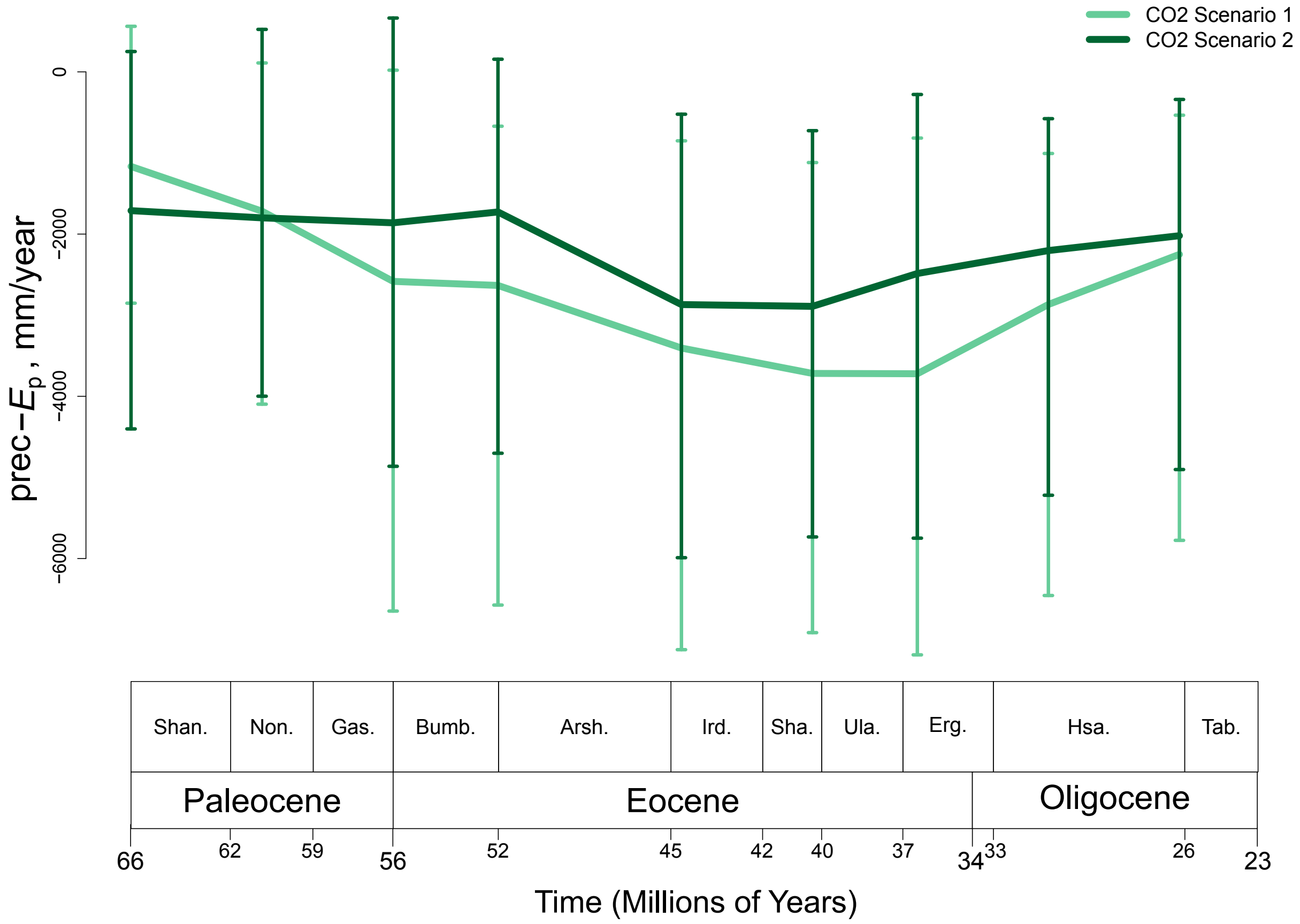

Figure S15. Water availability (prec-E_p the annual mean of precipitation minus potential evaporation) in Central Asia (averaged over 30° N - 55° N and 80° E - 120° E) simulated using the HadCM3 model (Valdes *et al.* [4]), calculated using two different pCO_2 estimates ('CO_2 Scenario 1', light green line and 'CO_2 Scenario 2', dark green line). ALMA abbreviations are explained in Fig. S1.

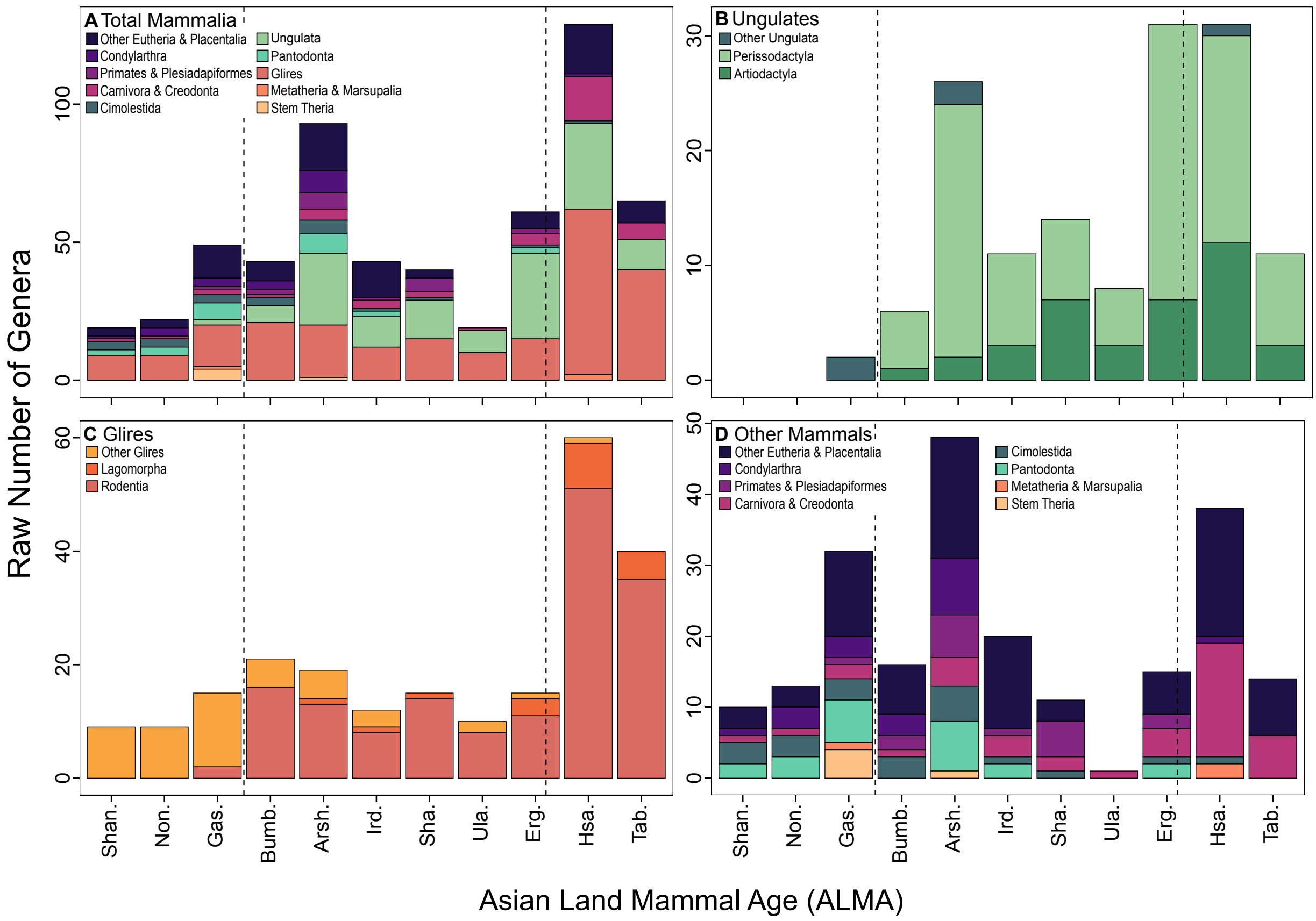

Figure S16. Raw number of genera per time bin (ALMAs, binned using midpoint age estimates) for a) total Mammalia, b) Ungulata, c) Glires and d) other Mammalia (all mammal clades except for ungulates and glires). Colours represent smaller clades, please refer to panel legends. ALMA time bin abbreviations are explained in Fig. S1.

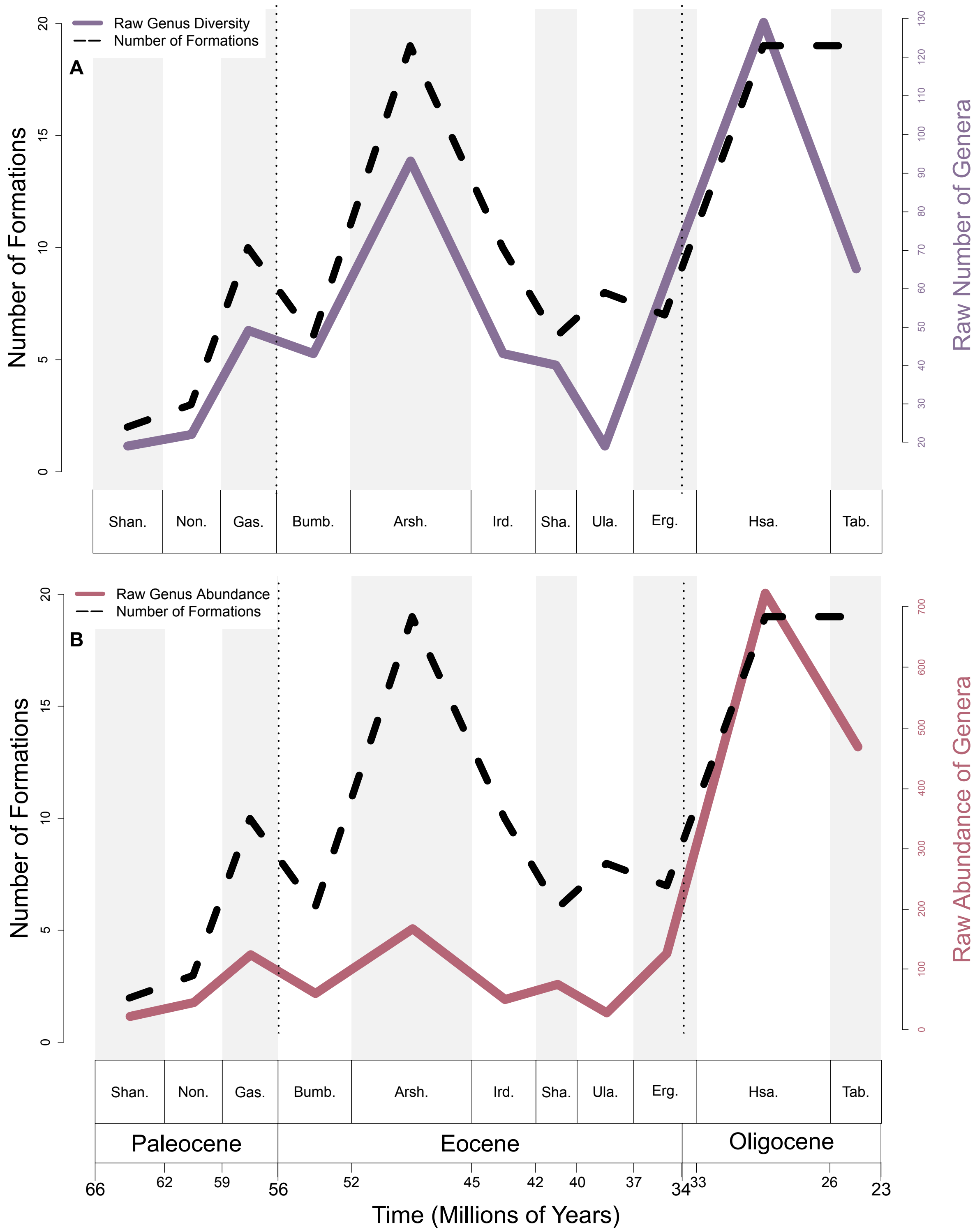

Figure S17. Raw number of mammal-bearing rock formations per time bin (ALMAs, binned using midpoint age estimates) plotted against a) raw number of unique mammalian genera per time bin and b) raw abundance of fossils identified to genus-level per time bin. ALMA time bin abbreviations are explained in Fig. S1.

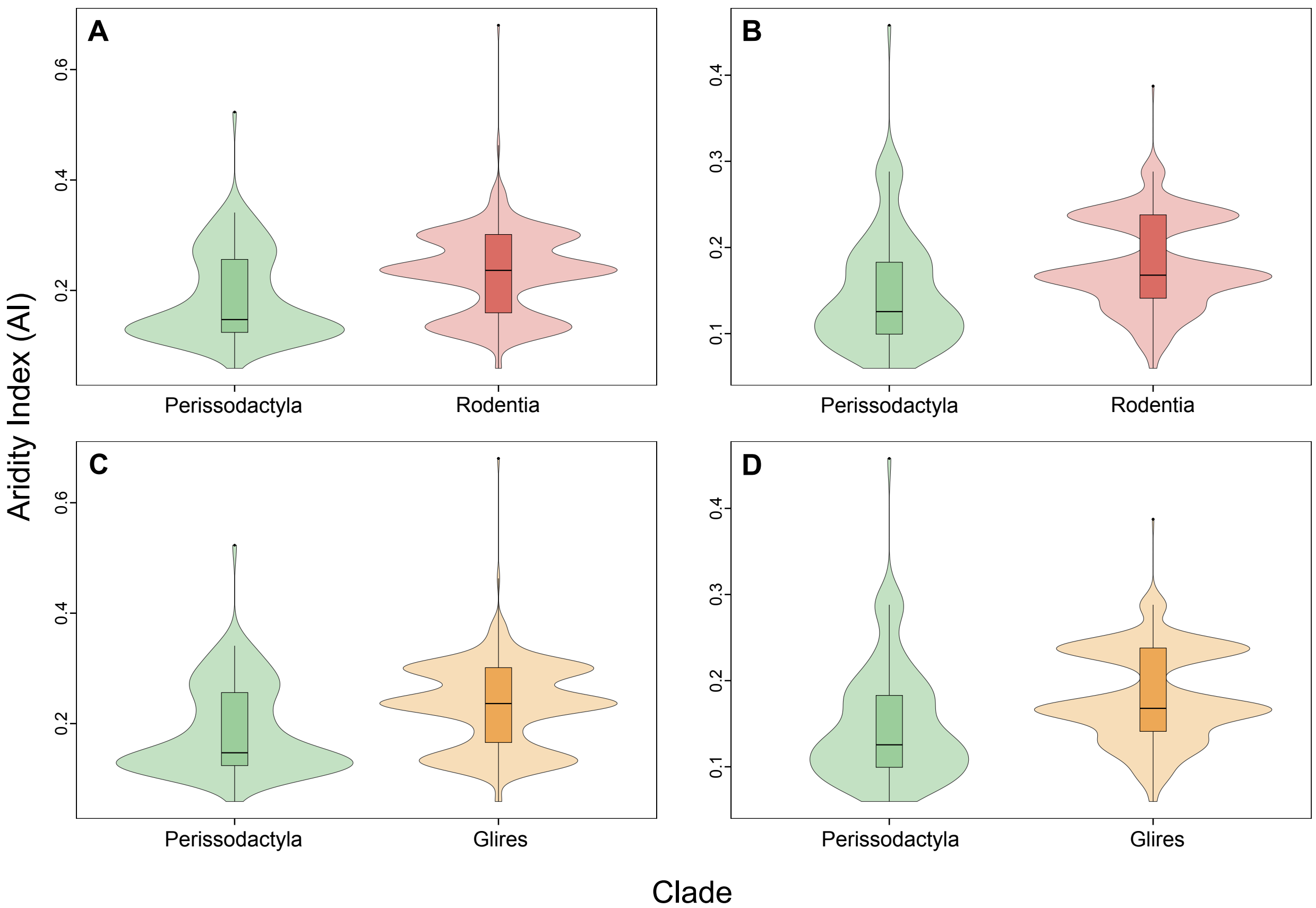

Figure S18. Violin and box plots showing the range and distribution of aridity index (AI) values for individual clades. a) Perissodactyla (green) and Rodentia (red), with AI estimated using 'CO_2 Scenario 2', b) Perissodactyla and Rodentia, with AI estimated using 'CO_2 Scenario 1', c) Perissodactyla and Glires (orange), with AI estimated using 'CO_2 Scenario 2', d) Perissodactyla and Glires, with AI estimated using 'CO_2 Scenario 1'. Box and whisker plots have a centre showing the median, bounds of the box showing the interquartile range and the whiskers showing the interval containing 90% of the data. Violin plot illustrating the approximated frequency distribution of individual values.

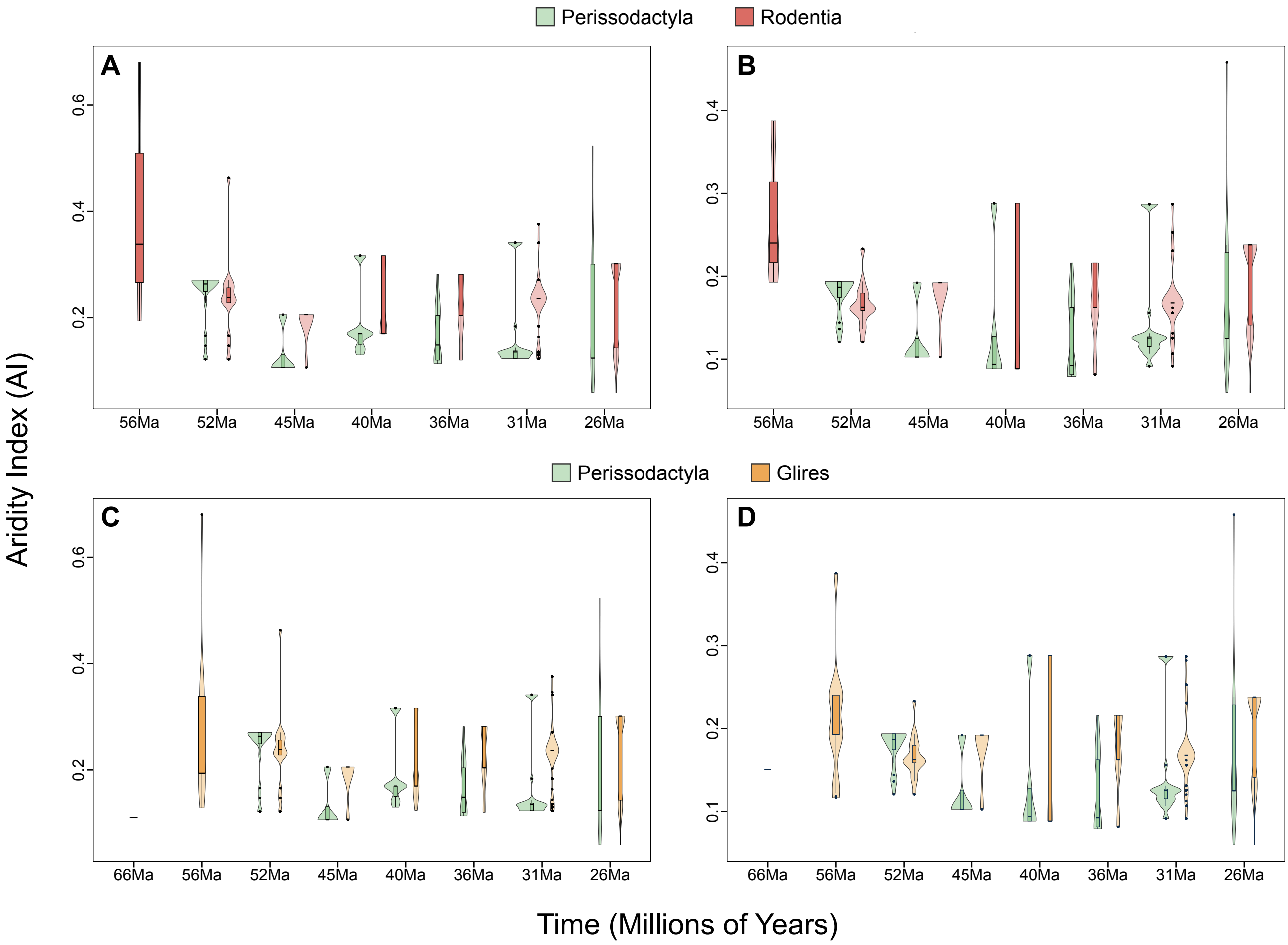

Figure S19. Violin and box plots showing the range and distribution of aridity index (AI) values for individual clades for each time interval. a) Perissodactyla (green) and Rodentia (red), with AI estimated using 'CO_2 Scenario 2', b) Perissodactyla and Rodentia, with AI estimated using 'CO_2 Scenario 1', c) Perissodactyla and Glires (orange), with AI estimated using 'CO_2 Scenario 2', d) Perissodactyla and Glires, with AI estimated using 'CO_2 Scenario 1'. Box and whisker plots have a centre showing the median, bounds of the box showing the interquartile range and the whiskers showing the interval containing 90% of the data. Violin plot illustrating the approximated frequency distribution of individual values.

References

1. Meng, J., McKenna, M.C., 1998. Faunal turnovers of Palaeogene mammals from the Mongolian Plateau. Nature 394, 364–367. https://doi.org/10.1038/28603

2. Peters, S.E., Quinn, D.P., Husson, J.M., Gaines, R.R., 2022. Macrostratigraphy: Insights into Cyclic and Secular Evolution of the Earth-Life System. Annu. Rev. Earth Planet. Sci. 50, 419–449. https://doi.org/10.1146/annurev-earth-032320-081427

3. Foster, G.L., Royer, D.L., Lunt, D.J., 2017. Future climate forcing potentially without precedent in the last 420 million years. Nat Commun 8, 14845. https://doi.org/10.1038/ncomms14845

4. Valdes, P.J., Scotese, C.R., Lunt, D.J., 2021. Deep ocean temperatures through time. Climate of the Past 17, 1483–1506. https://doi.org/10.5194/cp-17-1483-2021
