# Supplementary material for: Global and regional climate change impacted Paleogene mammal faunas in Central Asia
Source: Nat Commun. 2026 Sep 8;17:9606. doi: 10.1038/s41467-026-77374-7 (PMC13554191; doi:10.1038/s41467-026-77374-7)
Supplement: Supplementary file 4 [file 41467_2026_77374_MOESM2_ESM.pdf]

Description of Additional Supplementary Files

File Name - Supplementary Data 1.

Description - Body Size Through Time t-test Results. Multi-tab excel file containing the results of t tests performed on pairwise body mass distributions between neighboring bins, for Total Mammalia and all clades.

File Name - Supplementary Data 2.

Description - Bibliography of Primary Literature. Excel file containing references for all primary literature from which data was collected.

File Name - Supplementary Data 3.

Description - Clean Genus & Species Datasets. Multi-tab excel file containing the full, cleaned genus- and species-level datasets used in this study.

File Name - Supplementary Data 4.

Description - Time Interval Data frame. Multi-tab excel file containing the two-time binning schemes used in this study.

File Name - Supplementary Data 5.

Description - iNEXT Sampling Corrected Richness Results. Multi-tab excel file containing the iNEXT sampling corrected mammal richness through time results for all clades, versions of the datasets and quorums for each bootstrap replicate.

File Name - Supplementary Data 6.

Description - iNEXT Method Results. Multi-tab excel file containing the method used (observed, interpolated, extrapolated) to generate the iNEXT sampling corrected mammal richness through time results for all clades, versions of the datasets and quorums for each bootstrap replicate.

File Name - Supplementary Data 7.

Description - Goods u Results Multi-tab excel file containing the results of Good's u (coverage) for each time bin for all clades and versions of the datasets.

File Name - Supplementary Data 8.

Description - Turnover Results Multi-tab excel file containing the RAC sampling corrected bin-to-bin turnover results for all clades, versions of the datasets, quorums and methods used.

File Name Supplementary Code 1

Description - Zip file containing code for analyses.

*Benevento et al Asia Mammal Genus Richness. R Code that runs the genus richness analysis for total Mammalia. To run the species analysis instead use the species level dataset. To run all other clades, subset each dataset and run on a specific clade of interest.

*Benevento et al Asia Mammal Genus Turnover. R Code that runs genus richness analysis using the Sorenson statistics. To run modified Forbes, replace "Sorenson" with "Forbes".

*Benevento et al Asia Mammals Size Code. R Code that runs the body mass analyses for total Mammalia. To run all other clades, subset each dataset and run on a specific clade of interest.

*Benevento et al MST and Richness. R. Code that runs the MST and Richness analysis for total Mammalia to check for an area effect. To run all other clades, subset each dataset and run on a specific clade of interest.
